# Design, synthesis and antifungal activity of threoninamide carbamate derivatives via pharmacophore model

**DOI:** 10.1080/14756366.2020.1729144

**Published:** 2020-03-09

**Authors:** Xiu-Jiang Du, Xing-Jie Peng, Rui-Qi Zhao, Wei-Guang Zhao, Wei-Li Dong, Xing-Hai Liu

**Affiliations:** a State Key Laboratory of Elemental Organic Chemistry, Nankai University, Tianjin, China; b Tianjin Key Laboratory on Technologies Enabling Development of Clinical Therapeutics and Diagnostics (Theranostics), School of Pharmacy, Tianjin Medical University, Tianjin, China; c College of Chemical Engineering, Zhejiang University of Technology, Hangzhou, China

**Keywords:** Pharmacophore model, threoninamide carbamate derivatives, synthesis, antifungal activity, SAR

## Abstract

Thirty-six novel threoninamide carbamate derivatives were designed and synthesised using active fragment-based pharmacophore model. Antifungal activities of these compounds were tested against *Oomycete* fungi *Phytophthora capsici in vitro* and *in vivo.* Interestingly, compound **I-1, I-2, I-3, I-6** and **I-7** exhibited moderate control effect (>50%) against *Pseudoperonospora cubensis* in greenhouse at 6.25 μg/mL, which is better than that of control. Meanwhile most of these compounds exhibited significant inhibitory against *P. capsici*. The other nine fungi were also tested. More importantly, some compounds exhibited remarkably high activities against *Sclerotinia sclerotiorum*, *P. piricola* and *R. solan in vitro* with EC_50_ values of 3.74–9.76 μg/mL. It is possible that the model is reliabile and this method can be used to discover lead compounds for the development of fungicides.

## Introduction

Oomycete fungi can cause several destructive diseases in crops, vegetables and fruits, such as *Phytophthora infestans*, *Peronospora hyoscyami*, *Phytophthora capsici* and *Pseudoperonospora cubensis*^1^. The cell walls of the Oomycetes are different from other fungi, which contain cellulose, not chitin. So cellulose synthase represents a potential target for discovering new Oomycete inhibitors^2^, which can inhibit different stages in the life cycle of Oomycetes including mycelial growth, sporangium production, zoospore release and cystospore germination. Since dimethomorph^3^ was discovered by Shell company, seven carboxylic acid amide (CAA) fungicides^4^^,^^5^ including flumorph^6^, pyrimorph^7^, benthiavalicarb^8^, benthiavalicarb-isopropyl^9^, iprovalicarb^10^, valiphenal^11^, and mandipropamid^12^ were developed, which were divided as three different sub-classes by FRAC (www.frac.info) due to their common cross resistance pattern for the vast majority of Oomycetes. Since the dimethomorph was discovered as first CAA fungicide in 1988, only seven CAA fungicides are marketed until now.

However, it was still unknown that the structure of cellulose synthases in the *Oomycete* plant pathogen. Blum et al.^13^^,^^14^ reported that both mutations in the *PiCesA3* gene of *P. infestans* result in a change to the same amino acid (glycine-1105) in the protein and the mutations in *PiCesA3* were responsible for the mandipropamid insensitivity phenotype. The resistance mutants of some pathogens to CAA fungicides has been elucidated in recent reports^15^^,^^16^. However, purified protein of Oomycete of cellulose synthase is not available. In recent reference, her group^17^ built a modelling of the *P. capsici* cellulose synthase 3. In our previous work^18–35^, many bioactive compounds were designed and synthesised. In this paper, based on the structure of seven commercialised CAA fungicides, we found that they have similar structural fragments: amide bond, para-substituted phenyl, 3,4-dialkyloxy substituted phenyl. Only valinamide carbamates have two result fragments, so we established a pharmacophore model. The dialkyloxybenzene substructures were introduced into threoninamidecarbamates and designed the title compounds.

## Results and discussion

### Active-fragment-based pharmacophore model

**Figure 1.**
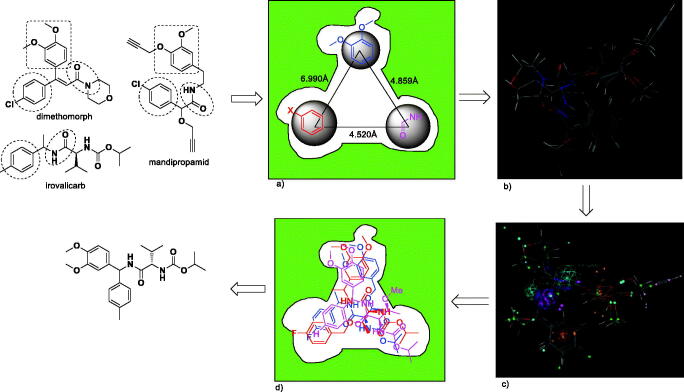
The pharmacophore model of three active fragments.

The key technical challenge for this approach was the detection of fragment hits. Traditionally, fragment hits were often found by conventional bioassay-based methods and biophysical methods (X-ray, NMR and surface plasmon resonance). However, in our previous work^36–39^, we find that three sub-classes fungicides have nearly identical structural fragments: including amide, halobenzene (or methylbenzene) and/or dialkoxyl benzene. The three fragments are exactly what we are looking for fragments with a good match with a target binding site, because any optimisation of the three fragments could lead to reduced antifungal activity. Furthermore, we noticed that valinamide carbamates only have two of three active structural fragments. In order to validate our idea, a new valinamide carbamate with three fragments was designed and synthesised (Figure 1). The compound was found to display higher *in vitro* antifungal activities against *P. capsici* (EC_50_ 0.15 μg/mL) than iprovalicarb (EC_90_ 0.27 μg/mL)^38^. This result prompted us to develop a new active-fragment-based drug discovery, which is especially suited if no purified protein and no structural information on the binding site are available.

After a careful analysis of the seven structures of CAA fungicides, it was found that the skeleton structure of cinnamic acid amides molecules is rigid, and the skeleton structure of the other two kind of molecules are flexible. So it is possible that the active fragments of valinamide carbamates and mandelic acid amides bind to the same pocket sites as that of cinnamic acid amides. From these fungicides, the distance between these active fragments is easily identified (Figure 1).

The three representative compounds (dimethomorph, iprovalicarb and mandipropamid) were performed using MOE. The 3D structures of the compounds were built by using the Builder option and geometry-optimized by using MMFF94x Forcefield and calculate forcefield partial charges. The three compounds were used successively for energy minimisation until the gradient value was smaller than 0.001 kcal/mol. The lowest-energy conformations of the three compounds were generated and the conformation of dimethomorph was served as templates in the study. Then the three compounds were aligned. The results are shown in Figure 1(b). Through three sub-types of molecular alignment, we built a pharmacophore model using SYBYL 6.9, which is shown in Figure 1(c). The results evaluated using pharmacophore scores. Threonine is an essential amino acid, which cannot be synthesised in humans. It’s structure is similar to valine. So a set of three threonine derivatives were designed and prepared using the above-described procedure for test case.

**Figure 2.**
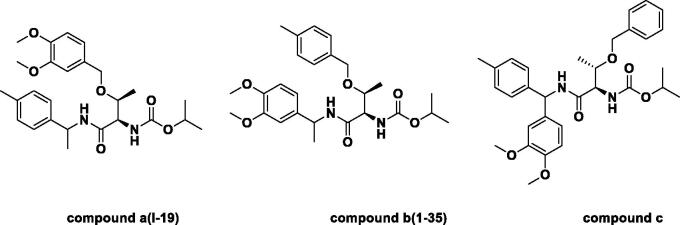
The three structures of designed threonine derivatives.

The compounds **a** and **b** showed good antifungal activity, their EC_50_ value were 3.49 and 3.10 μg/mL, respectively. Compound **c** bearing three benzene ring showed weaker (8.88 μg/mL) antifungal activity (Figure 2). From the structural analysis, it is possible that the compound **c** have three benzene rings, which make the crowded space and affect the activity. In order to find higher active compounds, another 33 threonine derivatives were synthesised and screened for antifungal against *P. capsici*, and their results are listed in Tables 2–4. The antifungal activity of compound **a**, **b** and **c** further indicated the model is reliable.

### Synthesis and spectra

**Scheme 1.**
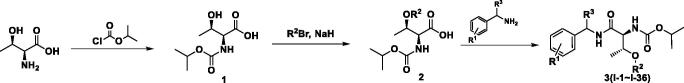
The synthetic route of threonine derivatives.

The synthesis procedures for compounds designed were shown in Scheme 1.

**Scheme 2.**
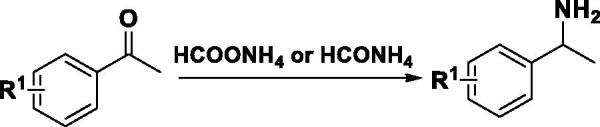
The general synthesis procedure of intermediate amine.

The intermediate amine was synthesised easily according to R. Lecchart reaction by using formamide and ketone as starting materials (Scheme 2). HCOONH_4_ was used as reactant under the solvent-free condition in the R. Lecchart reaction. When the reaction temperature heated to 160 °C, the reaction is violent and the yield is moderate (66%). In this article, the HCOONH_4_ was replaced by HCONH_4_ solution (5 quiv H_2_O), the reaction is mild and the yield increased (75%).

Isopropyl chloroformate is treated with L-threonine in aqueous sodium hydroxide solution to give isopropyloxycarbonyl-L-threonine **1**. The intermediate **1** was readily alkylated by sodium hydride in THF to alkoxy-substituted compounds **2**. And then, the carboxylic acid group of compounds **2** is activated by treating it with a equivalent of ethyl chloroformate under base conditions using tetrahydrofuran as a solvent. Finally, the mixed anhydride, which cannot be isolated, was treated with an auxiliary base and the amines intermediate in THF solution, as part of the same reaction step to form the title compounds **3** with the elimination of carbon dioxide and isopropanol in 60–88% yield. When R^3^ was methyl, designed compounds **3** could not be obtained by using the above-mentioned method. So L-threonine amides **4** were first synthesised by using the above-mentioned method. The free hydroxyl group of compounds **4** were then alkylated with iodomethane in the presence of silver oxide to obtain methyl-substituted compounds designed **3**.

### Antifungal activity and SAR

In our previous work^38^, valinamide carbamate compounds exhibited excellent antifungal activity against *P. capsici*, so the title threonine compounds were screened *in vitro* against *P. capsici* directly. The EC_50_ values for compounds were determined and the results are listed in Table 1. From Table 1, compounds **I-7, I-10, I-11, I-12, I-13, I-22, I-23, I-24, I-25, I-26** displayed excellent antifungal activity (EC_50_ < 1 μg/mL) against *P. capsici.* Especially compound **I-24**, the activity (EC_50_ = 0.14 μg/mL) is higher than that of control dimethomorph (EC_50_ = 0.37 μg/mL). The structure-activity relationship results showed that when R^3^ is Me group, R^1^ is *p*-Cl, *p*-Me, *p*-MeO, *p*-Pgoxy and 3-MeO-4-Pgoxy, R^2^ is Bn, *p*-FBn and *p*-ClBn, the compounds exhibited excellent activity, such as **I-3, I-7, I-10, I-22, I-23, I-24, I-25, I-26**. Among them, when R^1^ is *p*-Pgoxy or *p*-MeO and R^2^ is *p*-FBn, they exhibited highest antifungal activity, such as compound **I-10, I-23, I-24, I-25, I-26**. When the R^3^ of these compounds was replaced by phenyl group or substituted phenyl group, the activity decreased. These compounds exhibited weak antifungal activity, such as compound **I-13, I-14, I-15, I-16, I-17**.

**Table 1.** The EC_50_ values of threonine derivatives against *P. capsici*.

No.	Substituents			*y* = *a* + *bx*	*r*^2^	EC_50_ μg/mL
	R^1^	R^2^	R^3^			
**I-1**	H	Bn	Me	*y* = 4.3155 + 2.1632*x*	0.9428	2.07 ± 0.23
**I-2**	*p*-F	Bn	Me	*y* = 3.9699 + 2.1549*x*	0.9732	3.01 ± 0.26
**I-3**	*m*-F	Bn	Me	*y* = 4.6558 + 2.4035*x*	0.9324	1.39 ± 0.12
**I-4**	*o*-F	Bn	Me	*y* = 3.3251 + 1.9884*x*	0.9712	7.27 ± 0.55
**I-5**	*p*-Cl	Bn	Me	*y* = 3.6066 + 5.0563*x*	0.8993	1.24 ± 0.09
**I-6**	*p*-Me	Bn	Me	*y* = 4.9478 + 4.3944*x*	0.9642	2.05 ± 0.24
**I-7**	*p*-MeO	Bn	Me	*y* = 5.2613 + 2.7141*x*	0.9842	0.80 ± 0.05
**I-8**	*m*-MeO	Bn	Me	*y* = 3.1218 + 2.3592*x*	0.9701	6.26 ± 0.67
**I-9**	*o*-MeO	Bn	Me	*y* = 4.2385 + 1.9024*x*	0.9971	4.98 ± 0.88
**I-10**	*p*-Pgoxy	Bn	Me	*y* = 5.8080 + 2.4155*x*	0.9666	0.46 ± 0.03
**I-11**	3,4-diMeO	Bn	Me	*y* = 5.3971 + 1.5023*x*	0.6145	0.56 ± 0.03
**I-12**	3-MeO-4-Pgoxy	Bn	Me	*y* = 5.2252 + 1.5371*x*	0.9365	0.27 ± 0.16
**I-13**	3,4-diMeO	Bn	Ph	*y* = 3.6127 + 0.8547*x*	0.9941	42.06 ± 10.83
**I-14**	3,4-diMeO	Bn	*p*-FPh	*y* = 2.5553 + 1.5583*x*	0.9967	37.05 ± 5.44
**I-15**	3,4-diMeO	Bn	*p*-ClPh	*y* = 3.3650 + 1.0454*x*	0.9537	36.63 ± 7.32
**I-16**	3,4-diMeO	Bn	*p*-BrPh	*y* = 2.9715 + 1.4550*x*	0.9909	24.78 ± 2.99
**I-17**	3,4-diMeO	Bn	*p*-MePh	*y* = 0.2597 + 5.6567*x*	0.9286	8.88 ± 1.32
**I-18**	*p*-F	3,4-diMeOBn	Me	*y* = 4.3397 + 1.8195*x*	0.9477	2.31 ± 0.19
**I-19**	*p*-Me	3,4-diMeOBn	Me	*y* = 2.8108 + 4.2031*x*	0.9186	3.49 ± 0.55
**I-20**	*p*-Pgoxy	3,4-diMeOBn	Me	*y* = 4.3534 + 1.4515*x*	0.9868	2.80 ± 0.32
**I-21**	*p*-MeO	3,4-diMeOBn	Me	*y* = 4.0306 + 1.4599*x*	0.9478	4.63 ± 0.68
**I-22**	*p*-Me	*p*-FBn	Me	*y* = 6.0925 + 3.2941*x*	0.9436	0.48 ± 0.03
**I-23**	*p*-MeO	*p*-FBn	Me	*y* = 5.9086 + 2.9268*x*	0.9855	0.49 ± 0.03
**I-24**	*p*-Pgoxy	*p*-FBn	Me	*y* = 6.5167 + 1.6990*x*	0.7882	0.14 ± 0.02
**I-25**	3,4-diMeO	*p*-FBn	Me	*y* = 5.9157 + 7.1783*x*	0.9198	0.81 ± 0.03
**I-26**	3-MeO-4-Pgoxy	*p*-FBn	Me	*y* = 5.2526 + 2.3695*x*	0.9404	0.85 ± 0.05
**I-27**	3-MeO-4-Pgoxy	*p*-ClBn	Me	*y* = 4.5461 + 0.9908*x*	0.9826	2.64 ± 0.32
**I-28**	*p*-MeO	*p*-ClBn	Me	*y* = 4.1546 + 0.9741*x*	0.9975	7.93 ± 2.37
**I-29**	*p*-Pgoxy	*p*-ClBn	Me	*y* = 4.2866 + 0.8404*x*	0.9841	6.99 ± 1.30
**I-30**	*p*-MeO	*p*-MeOBn	Me	*y* = 4.6371 + 1.8188*x*	0.9913	1.59 ± 0.12
**I-31**	*p*-Pgoxy	*p*-MeOBn	Me	*y* = 4.8542 + 2.4195*x*	0.9967	1.26 ± 0.08
**I-32**	3-MeO-4-Pgoxy	*p*-MeOBn	Me	*y* = 3.9844 + 2.3635*x*	0.9867	1.77 ± 0.06
**I-33**	*p*-MeO	*p*-MeBn	Me	*y* = 3.9560 + 1.2618*x*	0.9974	6.72 ± 0.88
**I-34**	*p*-Pgoxy	*p*-MeBn	Me	*y* = 3.6684 + 1.4766*x*	0.9842	9.29 ± 1.06
**I-35**	3,4-diMeO	*p*-MeBn	Me	*y* = 1.7805 + 6.9131*x*	0.9695	3.10 ± 0.23
**I-36**	3-MeO-4-Pgoxy	*p*-MeBn	Me	*y* = 4.2233 + 1.7183*x*	0.9910	1.23 ± 0.30
dimethomorph				*y* = 6.5558 + 1.6550*x*	0.9930	0.37 ± 0.03

Based on results of the *in vitro* antifungal activity against *P. capsici*, compounds **I-1, I-2, I-3, I-6, I-7, I-10, I-18** and **I-23** were selected for further *in vivo* antifungal activity against *P. capsici* and *P. cubensis*. The results are listed in Table 2. From Table 2, compounds **I-1, I-3** and **I-6** exhibited good control effect (100%) against *P. capsici* at 50 μg/mL, which is the same as the control dimethomorph (100%). Unluckily, the compound **I-18** (65.2%) and **I-23** (83.6%) exhibited moderate *in vivo* antifungal activity, although the two compounds exhibited good *in vitro* antifungal activity. Interestingly, most of these compounds also exhibited good control effect (>80%) against *P. cubensis* at 50 μg/mL, which is better than the control dimethomorph (74.2%). Among them, only compound **I-23** (66.9%) exhibited moderate control effect against *P. cubensis* at 50 μg/mL. An extremely obvious phenomenon, the control effect decreased while the concentration dropped from 50 to 6.25 μg/mL. For example, most of these compounds exhibited moderate control effect against *P. capsici* at 25 μg/mL, except compound **I-1** (100%). But the control effect against *P. capsici* is lower than the control dimethomorph(100%) at 12.5 μg/mL, even at lower concentration. Notably, only compound **I-6** exhibited good control effect with the inhibitory of 66.0 ± 3.1 at 6.25 g/mL, which was weaker than that of the control dimethomorph (87.0%). But when the concentration was reduced to 3.125 μg/mL, the control effect is lower than that of control dimethomorph (62.5%). For the other fungal *P. cubensis*, most of tested compounds exhibited good control effect (>70%) at 50 μg/mL, which is the same as the control dimethomorph (74.2%). For instance, compounds **I-2** (96.7%), **I-6** (90.1%) displayed excellent control effect (>90%), which is much better than that of control dimethomorph (74.2%). The control effect of dimethomorph was 46.9% at 6.25 μg/mL, while the control effect of compounds **I-2, I-3** and **I-6** were all higher than 50%.

**Table 2.** The *in vivo* fungicidal activity against two fungi of some compounds.

No.	*P. capsici* (%, μg/mL)					*P. cubensis* (%, μg/mL)			
	50	25	12.5	6.25	3.125	50	25	12.5	6.25
**I-1**	100 ± 0	100 ± 0	47.8 ± 2.3	42.6 ± 1.6	30.4 ± 1.5	83.4 ± 1.5	63.5 ± 3.2	47.0 ± 2.3	40.3 ± 2.4
**I-2**	87.2 ± 1.3	53.2 ± 1.4	40.4 ± 1.6	36.2 ± 3.1	14.9 ± 2.1	96.7 ± 0.8	90.1 ± 1.5	73.5 ± 5.2	66.9 ± 3.3
**I-3**	100 ± 0	83.0 ± 0.9	66.0 ± 2.8	27.7 ± 2.5	14.9 ± 3.3	86.7 ± 3.2	66.9 ± 4.2	60.2 ± 4.4	60.2 ± 5.1
**I-6**	100 ± 0	61.7 ± 2.5	40.4 ± 3.4	66.0 ± 3.1	48.9 ± 5.2	90.1 ± 1.1	73.5 ± 3.3	66.9 ± 3.1	53.6 ± 2.1
**I-7**	83.0 ± 1.9	78.7 ± 3.6	74.5 ± 2.3	53.2 ± 1.6	nt	76.8 ± 4.2	66.9 ± 2.1	53.6 ± 6.8	47.0 ± 5.2
**I-10**	65.2 ± 4.6	65.2 ± 1.3	52.2 ± 3.9	30.4 ± 2.1	nt	73.9 ± 6.5	63.8 ± 1.5	52.7 ± 7.2	36.0 ± 1.8
**I-18**	69.6 ± 2.1	65.2 ± 0.5	30.4 ± 2.2	nt	nt	66.9 ± 2.2	58.2 ± 2.6	47.1 ± 2.5	39.7 ± 9.4
**I-23**	83.6 ± 3.2	68.2 ± 2.2	63.6 ± 1.5	45.5 ± 1.8	0	76.4 ± 1.3	62.8 ± 4.2	69.0 ± 4.2	55.0 ± 2.3
dimethomorph	100 ± 0	100 ± 0	100 ± 0	87.0 ± 2.1	62.5 ± 1.2	74.2 ± 2.5	69.0 ± 2.1	61.3 ± 2.5	46.9 ± 4.1

### *In vitro* other antifungal activity

The *in vitro* antifungal activity of title 16 compounds against *Fusarium oxysporum (FO), Cercospora arachidicola (CA), Physalospora piricola (PP),Alternaria solani (AS), Gibberella zeae (GZ), Phytophthora infestans (PI), Sclerotinia sclerotiorum (SS), Botrytis cinerea (BC)* and *Rhizoctonia solani (RS)* at 50 μg/mL was tested and listed in Table 3. Chlorothalonil and carbendazimwere used as the control.

**Table 3.** The *in vitro* fungicidal activity of compound at 50 μg/mL.

No.	FO	CA	PP	AS	GZ	PI	SS	BC	RS
I-1	18.2 ± 1.9	36.8 ± 2.2	81.4 ± 2.1	72.4 ± 1.6	nt	52.5 ± 2.6	87.0 ± 2.2	69.2 ± 2.5	90.8 ± 2.2
I-2	45.0 ± 2.1	45.5 ± 3.2	90.5 ± 0.9	62.5 ± 0.8	36.4 ± 1.6	53.3 ± 2.4	86.4 ± 1.6	66.7 ± 1.5	76.3 ± 3.1
I-3	13.6 ± 1.2	26.3 ± 1.7	43.0 ± 3.2	79.3 ± 1.8	nt	52.5 ± 1.7	82.6 ± 1.9	65.4 ± 4.1	81.5 ± 1.5
I-6	27.3 ± 1.7	52.6 ± 3.6	70.9 ± 2.4	62.1 ± 2.4	nt	52.5 ± 4.1	82.6 ± 2.8	71.2 ± 2.2	84.6 ± 2.2
I-7	18.2 ± 1.9	52.6 ± 2.7	76.7 ± 0.5	75.9 ± 1.5	nt	45.0 ± 3.3	87.0 ± 3.1	71.2 ± 1.4	90.8 ± 1.5
I-8	22.7 ± 0.3	47.4 ± 3.4	53.5 ± 1.4	75.9 ± 0.4	nt	50.0 ± 2.8	84.8 ± 2.1	67.3 ± 2.1	83.1 ± 1.7
I-9	18.2 ± 0.6	26.3 ± 2.3	69.8 ± 3.2	65.5 ± 3.2	nt	50.0 ± 1.9	89.1 ± 1.9	73.1 ± 5.1	89.2 ± 1.5
I-10	38.1 ± 3.1	57.9 ± 3.1	57.1 ± 4.1	57.1 ± 2.2	41.2 ± 1.7	52.6 ± 1.4	96.8 ± 3.2	80.4 ± 1.5	91.1 ± 2.1
I-14	40.0 ± 5.8	36.4 ± 2.5	66.7 ± 2.4	56.3 ± 1.7	27.3 ± 3.1	46.7 ± 3.5	86.4 ± 2.2	69.2 ± 2.4	65.8 ± 1.7
I-15	40.0 ± 3.6	27.3 ± 1.3	76.2 ± 3.1	43.8 ± 2.4	27.3 ± 2.8	33.3 ± 4.2	90.9 ± 1.5	69.2 ± 3.1	81.6 ± 2.1
I-16	45.0 ± 2.2	27.3 ± 1.0	52.4 ± 2.5	56.3 ± 3.1	31.8 ± 2.5	46.7 ± 4.4	90.9 ± 0.9	56.4 ± 0.9	72.4 ± 2.2
I-17	33.3 ± 1.7	29.4 ± 2.1	83.3 ± 1.3	52.6 ± 2.6	66.7 ± 4.1	43.5 ± 2.3	83.8 ± 1.7	55.2 ± 3.1	62.5 ± 7.2
I-18	68.8 ± 4.1	76.9 ± 4.5	84.0 ± 2.1	55.6 ± 1.9	23.5 ± 2.3	44.4 ± 3.3	90.3 ± 2.2	67.9 ± 4.1	90.3 ± 1.2
I-21	38.5 ± 2.6	58.8 ± 2.3	95.5 ± 0.7	73.7 ± 2.5	60.0 ± 1.9	44.0 ± 1.5	88.6 ± 1.5	74.4 ± 2.2	88.2 ± 1.4
I-23	42.3 ± 2.2	35.3 ± 2.5	100 ± 0	63.2 ± 1.5	60.0 ± 2.1	44.0 ± 2.1	79.5 ± 3.2	81.4 ± 1.6	84.3 ± 1.5
I-33	62.5 ± 1.8	76.9 ± 2.1	92.0 ± 1.4	72.7 ± 1.4	47.1 ± 1.3	50.0 ± 3.7	90.3 ± 1.2	64.3 ± 1.9	77.6 ± 2.3
Chlorothalonil	83.3 ± 0.4	75.0 ± 1.3	92.3 ± 0.3	73.9 ± 0.7	73.1 ± 0.4	81.0 ± 2.5	96.4 ± 0.5	96.1 ± 0.3	96.1 ± 0.7
Carbendazim	100 ± 0	8.3 ± 1.2	97.4 ± 0.5	43.5 ± 4.2	100 ± 0	100 ± 0	100 ± 0	9.8 ± 2.3	100 ± 0

“nt”: not test.

As shown in Table 3, most of the title compounds were found to exhibit certain antifungal activity. Among them, lots of compounds exhibited good fungicidal activity against *P. piricola*, *S. sclerotiorum* and *R. solani*. Among these compounds, **I-23** (100, 79.5 and 84.3%), **I-33** (92.0, 90.3 and 77.6%) also exhibited good activity against *P. piricola, S. sclerotiorum* and *R. Solani*, respectively. For the *F. oxysporum*, the compounds displayed moderate or low activity. Compounds **I-23** and **I-33** possessed good activity (76.9 and 76.9%) respectively, which is better than control Carbendazim (8.3%). All the compounds exhibited moderate inhibition against *A. solani,* (>70%), meanwhile, the control chlorothalonil and carbendazim also exhibited moderate activity (73.9 and 43.5%) against *A. solani.* For *G. zeae* and *P. infestans*, the control Carbendazim (100%) can kill them, but these compounds exhibited moderate activity (∼50%) against *G. zeae* and *P. infestans*. Compounds **I-10** (80.4%) and **I-23** (81.4%) possessed good antifungal activity against *B. Cinerea,* which is the same as the control Chlorothalonil (96.1%).

On the basis of the preliminary fungicidal activity results, compound **I-10, I-18, I-21, I-23, I-33**and Chlorothalonil were selected for further EC_50_ bioassays (> 90% inhibitory) and the results are shown in Table 4. From Table 4, compounds **I-21** and **I-33** exhibited good activity against *P. piricola,* which is better than that of control Chlorothalonil. For the *S. sclerotiorum,* only compound **I-10** displayed good activity, which is better than that of control Chlorothalonil. Among them, when the R^1^ is MeO or p-Pgoxy, R^3^ is Me, they exhibited best fungicidal activity for the three fungi respectively, such as compounds **I-33** and **I-10**.

**Table 4.** The EC_50_ of some compounds against three fungals (μg/mL).

No	*P. piricola*	*S. sclerotiorum*	*R. solani*
I-10	nt	4.00 ± 0.7	nt
I-18	nt	9.04 ± 1.5	9.49 ± 1.2
I-21	4.19 ± 0.3	8.80 ± 1.2	nt
I-23	7.01 ± 1.1	nt	nt
I-33	3.74 ± 1.6	9.76 ± 1.9	nt
Chlorothalonil	7.33 ± 0.4	5.78 ± 0.8	1.67 ± 0.2

nt: not test.

## Experimental

### Instrument

All the chemical reagents were analytical grade or prepared in our lab. Melting points were measured using an X-4 apparatus (Taike, Beijing, China) and were uncorrected. ^1^H NMR and 13C NMR spectra were recorded on BRUKER Advance 400 MHz spectrometer using CDCl_3_ as solvent. HRMS was determined on a FTMS 7.0 instrument.

### Synthesis

#### (2S, 3 R)-2-carbamate isopropyl-3-hydroxybutyric acid 1

L-threonine (35.8 g, 0.3 mol) was added into 2 N NaOH solution and stirred for 1 h, and isopropyl chloroformate (40.6 g, 0.36 mol) was added at 0 °C. The reaction was stopped by stirring at room temperature for 1 h, washed by ether (100 ml), then water phase was adjusted to pH 2–3 with 1 M dilute HCl, extracted by ether (80 ml*4), and dried by anhydrous MgSO_4_ to give **1**, white solid (48 g, yield 78.0%). m.p. 112–114 °C, ^1^H NMR (400 MHz, CDCl_3_) δ 5.04–4.84 (m, 1H, (CH_3_)_2_CHO), 4.43 (s, 1H, NHCHCOOH), 4.34 (s, 1H, CH_3_CHOH), 1.28 (d, *J* = 6.1 Hz, 9H, CH_3_CH).

#### 3-(benzyloxy)-2-((isopropoxycarbonyl)amino)butanoic acid

(2S, 3 R)−2-carbamate isopropyl-3-hydroxybutyric acid (5 g, 24.39 mmol) was dissolved in DMF (50 ml), then 60% NaH (2.93 g, 73.17 mmol) was added in batches at 0 °C. After stirring for 2 h, (chloromethyl)benzene (4.62g, 36.59 mmol) was added dropwise. The reaction was quenched by stirring for 5 h at room temperature. Then the mixture was poured into water (250 ml) and washed by ether (100 ml) once. the water phase was adjusted to pH 2–3 with 1 M dilute HCl, ether was extracted (80 ml*4), and dried by anhydrous MgSO_4_ to give **2a**, yellow oil, yield 52.1%, ^1^H NMR (400 MHz, CDCl_3_) δ 7.38–7.11 (m, 5H, Ar-H), 4.96 (s, 1H, NHCHCOOH), 4.40 (s, 1H, (CH_3_)_2_CHO), 4.13 (s, 1H, CH_3_CHOCH_2_), 3.09 (d, *J* = 6.9 Hz, 2H, Ar-CH_2_O), 1.52–0.99 (m, 9H, CH_3_). 3-((4-fluorobenzyl)oxy)−2-((isopropoxycarbonyl)amino)butanoic acid **2b**: yellow oil, yield 85.2%, ^1^H NMR (400 MHz, CDCl_3_) δ 9.41 (s, 1H, NHCHCOOH), 7.22 (dd, *J* = 8.4, 5.6 Hz, 2H, Ar-H), 6.98 (dd, *J* = 15.3, 6.6 Hz, 2H, Ar-H), 5.40 (d, *J* = 9.3 Hz, 1H, NHCHCOOH), 4.91 (td, *J* = 12.4, 6.2 Hz, 1H, NHCHCOOH), 4.59–4.34 (m, 2H, Ar-CH_2_O), 4.24–4.13 (m, 1H, (CH_3_)_2_CHO), 3.12–2.79 (m, 1H, CH_3_CHOCH_2_), 1.52–1.07 (m, 9H, CH_3_). 3-((4-chlorobenzyl)oxy)−2-((isopropoxycarbonyl)amino)butanoic acid **2c**: yellow solid, mp 125–126 °C, yield 74.3%, ^1^H NMR (400 MHz, CDCl_3_) δ 9.11 (s, 1H, NHCHCOOH), 7.38–7.29 (m, 2H, Ar-H), 7.18 (d, *J* = 7.9 Hz, 2H, Ar-H), 5.40 (d, *J* = 9.0 Hz, 1H, NHCHCOOH), 4.55 (d, *J* = 11.8 Hz, 1H, NHCHCOOH), 4.40 (dd, *J* = 16.4, 10.7 Hz, 2H, Ar-CH_2_O), 4.25–4.10 (m, 1H, (CH_3_)_2_CHO), 3.49 (d, *J* = 6.8 Hz, 1H, CH_3_CHOCH_2_), 1.23 (dd, *J* = 19.8, 6.5 Hz, 9H, CH_3_). 2-((isopropoxycarbonyl)amino)−3-((4-methylbenzyl)oxy)butanoic acid **2d**: yellow solid, mp 122–124 °C, yield 65.3%, ^1^H NMR (400 MHz, CDCl_3_) δ 10.04 (s, 1H, NHCHCOOH), 7.13 (d, *J* = 9.2 Hz, 4H, Ar-H), 5.49 (d, *J* = 8.9 Hz, 1H, NHCHCOOH), 4.95 (t, *J* = 12.7 Hz, 1H, NHCHCOOH), 4.54 (s, 2H, Ar-CH_2_O), 4.19 (septet, 1H, (CH_3_)_2_CHO), 3.58 (m, 1H, CH_3_CHOCH_2_), 2.33 (d, *J* = 9.1 Hz, 3H, Ar-CH_3_), 1.29 (m, 9H, CH_3_). 2-((isopropoxycarbonyl)amino)−3-((4-methoxybenzyl)oxy)butanoic acid 2e: yellow oil, yield 69.6%, ^1^H NMR (400 MHz, CDCl_3_) δ 7.16 (t, *J* = 18.9 Hz, 2H, Ar-H), 6.86 (t, *J* = 8.7 Hz, 2H, Ar-H), 5.43 (d, *J* = 8.8 Hz, 1H, NHCHCOOH), 4.69–4.32 (m, 2H, Ar-CH_2_O), 4.26–4.07 (m, 1H, NHCHCOOH), 3.79 (d, *J* = 6.0 Hz, 3H, Ar-OCH_3_), 3.53 (m, 1H, (CH_3_)_2_CHO), 2.95 (d, *J* = 33.4 Hz, 1H, CH_3_CHOCH_2_), 1.23 (m, 9H, CH_3_). 3-((3,4-dimethoxybenzyl)oxy)−2-((isopropoxycarbonyl)amino)butanoic acid 2f: yellow oil, yield 61.2%, ^1^H NMR (400 MHz, CDCl_3_) δ 6.99–6.58 (m, 3H, Ar-H), 5.45 (t, *J* = 29.5 Hz, 1H, NHCHCOOH), 4.90 (s, 1H, NHCHCOOH), 4.63–4.32 (m, 2H, Ar-CH_2_O), 4.17 (s, 1H, (CH_3_)_2_CHO), 3.96–3.74 (m, 6H, Ar-OCH_3_), 3.55–3.40 (m, 1H, CH_3_CHOCH_2_), 1.46–1.05 (m, 9H, CH_3_).

### Isopropyl ((2S,3R)-1-oxo-1-((1-phenylethyl)amino)-3-(prop-2-yn-1-yloxy)butan-2-yl)carbamate 3

Intermediate **2** (4.12 mmol) was dissolved in THF (50 ml), then triethylamine (0.50 g, 4.94 mmol) and ethyl chloroformate (0.45 g, 4.12 mmol) were added at 0 °C. The mixture was stirred for 1 h under this condition. The solution of substituted acetophenone amine (4.94 mmol) in THF (15 ml) was dropwised into the reaction solution. The mixture was further stirred for 5 h at room temperature. Remove the solvent, the residue was dissolved in ether, washed by dilute hydrochloric acid, saturated NaHCO_3_ washing, dried by anhydrous MgSO_4_, column chromatography separation (petroleum ether: ethyl acetate = 5:1) to obtain the target compound 3(I-1∼I-36). The detailed data can be found in supporting information.

**Data for I-1.** white solid, mp 95 − 97 °C. ^1^H NMR (400 MHz, CDCl_3_) δ 7.40–7.28 (m, 5H, Ar-H), 7.25–7.16 (m, 5H, Ar-H), 6.80 (s, 1H, Ar-CHNH), 5.63 (s, 1H, CHN**H**CO), 5.10 (s, 1H, Ar-CHCH_3_), 4.94–4.85 (m, 1H, (CH_3_)_2_CHO), 4.66–4.47 (m, 2H, Ar-CH_2_O), 4.32 (s, 1H, COCHNH), 4.17 (d, *J* = 17.1 Hz, 1H, CH_3_CHCH), 1.44 (dd, *J* = 12.6, 6.9 Hz, 3H, Ar-CHCH_3_), 1.25 (s, 6H, (CH_3_)_2_CHO), 1.06 (d, *J* = 6.0 Hz, 3H, CH_3_CHCH). HRMS calcd for C_23_H_30_N_2_O_4_ ([M + Na]): 421.2098; Found: 421.2098.

**Data for I-2.** white solid, mp 117 − 119 °C. ^1^H NMR (400 MHz, CDCl_3_) δ 7.42–7.26 (m, 5H, Ar-H), 7.18 (ddd, *J* = 24.3, 8.4, 5.5 Hz, 2H, Ar-H), 6.94 (dt, *J* = 23.6, 8.5 Hz, 2H, Ar-H), 6.75 (d, *J* = 8.7 Hz, 1H, Ar-CHNH), 5.61 (s, 1H, CHNHCO), 5.18–5.01 (m, 1H, Ar-CHCH_3_), 4.89 (dt, *J* = 12.2, 6.0 Hz, 1H, (CH_3_)_2_CHO), 4.56 (dt, *J* = 21.1, 11.2 Hz, 2H, Ar-CH_2_O), 4.32 (s, 1H, COCHNH), 4.15 (ddd, *J* = 9.2, 8.2, 4.9 Hz, 1H, CH_3_CHCH), 1.41 (dd, *J* = 12.1, 6.9 Hz, 3H, Ar-CHCH_3_), 1.24 (dd, *J* = 6.2, 3.9 Hz, 6H, (CH_3_)_2_CHO), 1.05 (d, *J* = 6.1 Hz, 3H, CH_3_CHCH). HRMS calcd for C_23_H_29_FN_2_O_4_ ([M + Na]): 439.2004; Found: 439.2005.

**Data for I-3.** white solid, mp 110 − 112 °C. ^1^H NMR (400 MHz, CDCl_3_) δ 7.41–7.26 (m, 5H, Ar-H), 7.25–7.16 (m, 1H, Ar-H), 7.07–6.89 (m, 3H, Ar-H), 6.78 (d, *J* = 9.9 Hz, 1H, Ar-CHNH), 5.62 (s, 1H, CHNHCO), 5.15–5.04 (m, 1H, Ar-CHCH_3_), 5.01–4.83 (m, 1H, (CH_3_)_2_CHO), 4.58 (dt, *J* = 24.2, 11.4 Hz, 2H, Ar-CH_2_O), 4.33 (s, 1H, COCHNH), 4.25–4.11 (m, 1H, CH_3_CHCH), 1.41 (dd, *J* = 15.0, 6.9 Hz, 3H, Ar-CHCH_3_), 1.28–1.21 (m, 6H, (CH_3_)_2_CHO), 1.13 (dd, *J* = 49.1, 6.2 Hz, 3H, CH_3_CHCH). ^13 ^C NMR (101 MHz, CDCl_3_) δ 170.82, 164.18, 161.73, 156.69, 145.67, 137.87, 130.18, 121.78, 114.21, 112.94, 74.96, 74.69, 71.63, 68.86, 57.51, 48.58, 22.02, 15.11. HRMS calcd for: C_23_H_29_ClN_2_O_4_ ([M + Na]^+^): 455.1708; Found: 455.1712.

**Data for I-4.** white solid, mp 130 − 132 °C. ^1^H NMR (400 MHz, CDCl_3_) δ 7.35 (dd, *J* = 15.7, 6.2 Hz, 5H, Ar-H), 7.27 (s, 2H, Ar-H), 7.20 (s, 1H, Ar-CHNH), 7.04 (d, *J* = 5.6 Hz, 2H, Ar-H), 5.65 (s, 1H, CHNHCO), 5.31 (dd, *J* = 14.4, 7.3 Hz, 1H, Ar-CHCH_3_), 4.99–4.87 (m, 1H, (CH_3_)_2_CHO), 4.60 (dt, *J* = 18.5, 11.5 Hz, 2H, Ar-CH_2_O), 4.35 (s, 1H, COCHNH), 4.23–4.08 (m, 1H, CH_3_CHCH), 1.48 (dd, *J* = 12.1, 7.0 Hz, 3H, Ar-CHCH_3_), 1.27 (dd, *J* = 6.0, 2.6 Hz, 6H, (CH_3_)_2_CHO), 1.15 (dd, *J* = 54.2, 6.1 Hz, 3H, CH_3_CHCH). ^13 ^C NMR (101 MHz, CDCl_3_) δ 168.56, 163.41, 160.14, 157.14, 138.92, 129.94, 128.96, 128.49, 127.75, 124.30, 115.98, 115.75, 74.68, 71.62, 68.73, 57.54, 45.25, 22.08, 21.52, 15.37. HRMS calcd for C_23_H_29_FN_2_O_4_ ([M + Na]): 439.2004; Found: 439.2010.

**Data for I-5.** white solid, mp 158 − 160 °C. ^1^H NMR (400 MHz, CDCl_3_) δ 7.38–7.30 (m, 3H, Ar-H), 7.30–7.22 (m, 3H, Ar-H), 7.17 (d, *J* = 8.3 Hz, 2H, Ar-H), 7.11 (d, *J* = 8.4 Hz, 1H, Ar-H), 6.77 (s, 1H, Ar-CHNH), 5.60 (s, 1H, CHNHCO), 5.05 (s, 1H, Ar-CHCH_3_), 4.91 (dd, *J* = 18.0, 10.7 Hz, 1H, (CH_3_)_2_CHO), 4.60 (t, *J* = 12.4 Hz, 1H, COCHNH), 4.32 (s, 2H, Ar-CH_2_O), 4.24–4.09 (m, 1H, CH_3_CHCH), 1.40 (dd, *J* = 13.0, 6.9 Hz, 3H, Ar-CHCH_3_), 1.27–1.21 (m, 6H, (CH_3_)_2_CHO), 1.20–1.01 (m, 3H, CH_3_CHCH). ^13 ^C NMR (101 MHz, CDCl_3_) δ 169.38, 168.72, 156.14, 141.57, 137.88, 135.11, 132.98, 128.95, 74.86, 72.96, 71.64, 68.78, 62.09, 57.41, 48.42, 47.16, 23.74, 14.12. HRMS calcd for C_23_H_29_ClN_2_O_4_ ([M + Na]): 455.1708; Found: 455.1712.

**Data for I-6.** white solid, mp 135 − 137 °C. ^1^H NMR (400 MHz, CDCl_3_) δ 7.42–7.22 (m, 5H, Ar-H), 7.18–7.01 (m, 4H, Ar-H), 6.77 (d, *J* = 6.8 Hz, 1H, Ar-CHNH), 5.61 (d, *J* = 7.0 Hz, 1H, CHNHCO), 5.07 (s, 1H, Ar-CHCH_3_), 4.90 (dd, *J* = 11.2, 5.2 Hz, 1H, (CH_3_)_2_CHO), 4.67–4.47 (m, 2H, Ar-CH_2_O), 4.31 (s, 1H, COCHNH), 4.22–4.09 (m, 1H, CH_3_CHCH), 2.32 (d, *J* = 4.9 Hz, 3H, Ar-CH_3_), 1.42 (dd, *J* = 12.5, 6.9 Hz, 3H, Ar-CHCH_3_), 1.27–1.20 (m, 6H, (CH_3_)_2_CHO), 1.13 (dd, *J* = 49.0, 6.3 Hz, 3H, CH_3_CHCH). ^13 ^C NMR (101 MHz, CDCl_3_) δ 168.44, 157.62, 139.96, 136.97, 129.32, 128.47, 127.78, 126.03, 75.03, 74.77, 71.61, 68.76, 57.51, 48.77, 30.97, 22.04, 21.08, 15.28. HRMS calcd for C_24_H_32_N_2_O_4_ ([M + Na]): 435.2254; Found: 435.2250.

**Data for I-7.** white solid, mp 125 − 127 °C. ^1^H NMR (400 MHz, CDCl_3_) δ 7.39–7.22 (m, 5H, Ar-H), 7.15 (dd, *J* = 21.5, 8.6 Hz, 2H, Ar-H), 6.83 (d, *J* = 8.7 Hz, 1H, Ar-CHNH), 6.77 (d, *J* = 8.5 Hz, 2H, Ar-H), 5.75–5.53 (m, 1H, CHNHCO), 5.06 (s, 1H, Ar-CHCH_3_), 4.95–4.85 (m, 1H, (CH_3_)_2_CHO), 4.67–4.49 (m, 2H, Ar-CH_2_O), 4.31 (s, 1H, COCHNH), 4.14 (dd, *J* = 13.5, 10.6 Hz, 1H, CH_3_CHCH), 3.78 (d, *J* = 7.6 Hz, 3H, Ar-OCH_3_), 1.42 (dd, *J* = 12.0, 6.9 Hz, 3H, Ar-CHCH_3_), 1.24–1.21 (m, 6H, (CH_3_)_2_CHO), 1.13 (dd, *J* = 53.8, 7.4 Hz, 3H, CH_3_CHCH). ^13 ^C NMR (101 MHz, CDCl_3_) δ 169.67, 158.77, 135.07, 128.48, 127.84, 127.71, 127.24, 113.97, 75.02, 74.79, 71.60, 68.78, 57.86, 55.29, 48.43, 32.10, 29.72, 22.10, 15.56. HRMS calcd for C_24_H_32_N_2_O_5_ ([M + Na]): 451.2203; Found: 451.2200.

**Data for I-8.** white solid, mp 90 − 92 °C. ^1^H NMR (400 MHz, CDCl_3_) δ 7.46–7.08 (m, 5H, Ar-H), 6.81 (dt, *J* = 12.1, 7.4 Hz, 4H, Ar-H), 7.09 (d, *J* = 8.0 Hz, 1H, Ar-CHNH), 5.65 (s, 1H, CHNHCO), 5.18–5.02 (m, 1H, Ar-CHCH_3_), 4.89 (dd, *J* = 12.3, 6.1 Hz, 1H, (CH_3_)_2_CHO), 4.70–4.49 (m, 2H, Ar-CH_2_O), 4.33 (s, 1H, COCHNH), 4.23–4.08 (m, 1H, CH_3_CHCH), 3.76 (d, *J* = 7.8 Hz,3H, Ar-OCH_3_), 1.49–1.37 (m, 3H, Ar-CHCH_3_), 1.28–1.22 (m, 6H, (CH_3_)_2_CHO), 1.16–1.02 (m, 3H, CH_3_CHCH);^13^C NMR (101 MHz, CDCl_3_) δ 171.58, 159.80, 144.60, 129.74, 128.49, 127.88, 118.30, 112.50, 75.48, 74.73, 71.63, 68.95, 58.12, 55.17, 49.02, 31.94, 29.71, 22.71, 22.07. HRMS calcd for C_24_H_32_N_2_O_5_ ([M + Na]): 451.2203; Found: 451.2209.

**Data for I-9.** white solid, mp 99 − 101 °C. ^1^H NMR (400 MHz, CDCl_3_) δ 7.38–7.29 (m, 5H, Ar-H), 7.26–7.15 (m, 3H, Ar-H), 6.94–6.90 (m, 1H, Ar-H), 6.87 (d, *J* = 8.0 Hz, 1H, Ar-CHNH),5.66 (d, *J* = 6.5 Hz, 1H, CHNHCO), 5.34 − 5.21 (m, 1H, Ar-CHCH_3_), 5.00–4.85 (m, 1H, (CH_3_)_2_CHO), 4.63–4.50 (m, 2H, Ar-CH_2_O), 4.31 (s, 1H, COCHNH), 4.20 (dd, *J* = 15.2, 8.2 Hz, 1H, CH_3_CHCH), 3.78–3.57 (m, 3H, Ar-OCH_3_), 1.48–1.38 (m, 3H, Ar-CHCH_3_), 1.24 (d, *J* = 7.6 Hz, 6H, (CH_3_)_2_CHO), 1.21–1.02 (m, 3H, CH_3_CHCH). ^13 ^C NMR (101 MHz, CDCl_3_) δ 168.54, 156.97, 135.15, 130.47, 128.96, 128.48, 128.40, 128.23, 127.88, 127.73, 120.81, 110.88, 71.65, 68.67, 62.53, 57.63, 55.08, 47.17, 29.72, 22.09, 14.13. HRMS calcd for C_24_H_32_N_2_O_5_ ([M + Na]): 451.2203; Found: 451.2206.

**Data for I-10.** white solid, mp 136 − 137 °C.^1^HNMR (400 MHz, CDCl_3_) δ 7.39–7.23 (m, 5H, Ar-H), 7.16 (dd, *J* = 22.4, 8.6 Hz, 2H, Ar-H), 6.88 (dd, *J* = 22.1, 8.6 Hz, 2H, Ar-H), 6.74 (s, 1H, Ar-CHNH), 5.61 (s, 1H, CHNHCO), 5.06 (s, 1H, Ar-CHCH_3_), 4.89 (dt, *J* = 12.3, 6.0 Hz, 1H, (CH_3_)_2_CHO), 4.66 (dd, *J* = 8.3, 2.3 Hz, 2H, Ar-CH_2_O), 4.63–4.47 (m, 2H, CHCCH_2_O), 4.31 (s, 1H, COCHNH), 4.14 (dd, *J* = 12.6, 9.8 Hz, 1H, CH_3_CHCH), 2.51 (dd, *J* = 5.2, 2.5 Hz, 1H, CHCCH_2_O), 1.42 (dd, *J* = 12.0, 6.9 Hz, 3H, Ar-CHCH_3_), 1.28–1.20 (m, 6H, (CH_3_)_2_CHO), 1.13 (dd, *J* = 52.4, 6.4 Hz, 3H, CH_3_CHCH). ^13 ^C NMR (101 MHz, CDCl_3_) δ 168.45, 156.72, 139.25, 136.01, 128.50, 127.86, 127.26, 114.94, 78.55, 75.58, 74.77, 71.59, 68.78, 57.57, 55.80, 48.36, 22.10, 21.88, 15.27. HRMS calcd for C_26_H_32_N_2_O_5_ ([M + Na]): 475.2203; Found: 475.2207.

**Data for I-11.** white solid, mp 122 − 124 °C. ^1^H NMR (400 MHz, CDCl_3_) δ 7.30 (dd, *J* = 20.8, 9.5 Hz, 5H, Ar-H), 6.93 (dd, *J* = 24.2, 8.1 Hz, 1H, Ar-CHNH), 6.78 (d, *J* = 20.3 Hz, 3H, Ar-H), 5.62 (s, 1H, CHNHCO), 5.07 (s, 1H, Ar-CHCH_3_), 4.96–4.84 (m, 1H, (CH_3_)_2_CHO), 4.73 (d, *J* = 4.4 Hz, 2H, CHCH_2_O), 4.59 (t, *J* = 16.6 Hz, 2H, Ar-CH_2_O), 4.32 (s, 1H, COCHNH), 4.15 (s, 1H, CH_3_CHCH), 3.78 (d, *J* = 13.3 Hz, 3H), 2.49 (s, 1H, CHCH_2_O), 1.43 (dd, *J* = 13.5, 6.9 Hz, 3H, Ar-CHCH_3_), 1.23 (d, *J* = 5.9 Hz, 6H, (CH_3_)_2_CHO), 1.08 (d, *J* = 5.6 Hz, 3H, CH_3_CHCH). ^13 ^C NMR (101 MHz, CDCl_3_) δ 168.51, 156.26, 149.64, 145.98, 137.88, 136.99, 128.50, 127.87, 127.67, 117.81, 114.25, 110.18, 78.60, 75.84, 74.79, 71.57, 68.80, 57.49, 56.73, 55.83, 48.64, 22.10, 21.84, 15.25. HRMS calcd for C_27_H_34_N_2_O_6_ ([M + Na]): 505.2309; Found: 505.2314.

**Data for I-12.** white solid, mp 117 − 119 °C. ^1^H NMR (400 MHz, CDCl_3_) δ 7.39–7.20 (m, 5H, Ar-H), 6.98–6.85 (m, 1H, Ar-CHNH), 6.83–6.72 (m, 3H, Ar-H), 5.63 (s, 1H, CHNHCO), 5.06 (s, 1H, Ar-CHCH_3_), 4.88 (dd, *J* = 12.1, 6.2 Hz, 1H, (CH_3_)_2_CHO), 4.59 (t, *J* = 17.2 Hz, 2H, Ar-CH_2_O), 4.32 (s, 1H, COCHNH), 4.14 (s, 1H, CH_3_CHCH), 3.91–3.72 (m, 6H, Ar-OCH_3_), 1.43 (dd, *J* = 13.7, 6.9 Hz, 3H, Ar-CHCH_3_), 1.23 (d, *J* = 6.1 Hz, 6H, (CH_3_)_2_CHO), 1.07 (d, *J* = 6.1 Hz, 3H, CH_3_CHCH). ^13 ^C NMR (101 MHz, CDCl_3_) δ 168.58, 157.23, 148.95, 148.21, 137.90, 135.55, 128.47, 127.88, 127.64, 118.02, 111.13, 109.66, 74.89, 71.63, 68.75, 57.44, 55.83, 48.69, 22.06, 21.89, 15.23. HRMS calcd for C_27_H_34_N_2_O_6_ ([M + Na]): 505.2309; Found: 482.2417.

**Data for I-13.** white solid, mp 132 − 135 °C. ^1^H NMR (400 MHz, CDCl_3_) δ 7.32–7.24 (m, 5H, Ar-H), 7.22–7.10 (m, 5H, Ar-H), 6.72 (ddd, *J* = 27.2, 16.5, 10.8 Hz, 3H, Ar-H), 6.18 (d, *J* = 7.7 Hz, 1H, Ar-CHNH), 5.65 (s, 1H, Ar-CH-Ar), 4.98–4.84 (m, 1H, (CH_3_)_2_CHO), 4.66–4.49 (m, 2H, Ar-CH_2_O), 4.41 (s, 1H, COCHNH), 4.18 (dd, *J* = 6.3, 2.9 Hz, 1H, CH_3_CHCH), 3.79 (dd, *J* = 46.1, 9.3 Hz, 6H, Ar-OCH_3_), 1.27–1.18 (m, 6H, (CH_3_)_2_CHO), 1.15 (d, *J* = 6.2 Hz, 3H, CH_3_CHCH). HRMS calcd for C_30_H_36_N_2_O_6_ ([M + Na]): 543.2466; Found: 543.2471.

**Data for I-14.** white solid, mp 115 − 117 °C. ^1^H NMR (400 MHz, CDCl_3_) δ 7.26 (d, *J* = 35.2 Hz, 5H, Ar-H), 7.14 (d, *J* = 25.0 Hz, 2H, Ar-H), 6.98 (dd, *J* = 18.0, 9.1 Hz, 2H, Ar-H), 6.73 (dd, *J* = 49.0, 17.0 Hz, 3H, Ar-H), 6.18 (s, 1H, Ar-CHNH), 5.66 (s, 1H, Ar-CH-Ar), 4.93 (s, 1H, (CH_3_)_2_CHO), 4.59 (dd, *J* = 30.3, 10.8 Hz, 2H, Ar-CH_2_O), 4.45 (d, *J* = 18.8 Hz, 1H, COCHNH), 4.20 (s, 1H, CH_3_CHCH), 3.94–3.67 (m, 6H, Ar-OCH_3_), 1.25 (s, 6H, (CH_3_)_2_CHO), 1.17 (s, 3H, CH_3_CHCH). ^13 ^C NMR (101 MHz, CDCl_3_) δ 170.11, 164.25, 161.50, 157.19 149.12, 148.80, 137.22, 133.65, 128.77, 128.50, 127.66, 119.58, 115.55, 115.35, 111.07, 110.61, 75.00, 71.73, 68.88, 57.61, 56.12, 55.91, 22.06, 15.17. HRMS calcd for C_30_H_35_FN_2_O_6_ ([M + Na]): 561.2371; Found: 561.2373.

**Data for I-15.** white solid, mp 118 − 120 °C. ^1^H NMR (400 MHz, CDCl_3_) δ 7.30 (dd, *J* = 14.9, 7.0 Hz, 5H, Ar-H), 7.21 (d, *J* = 3.7 Hz, 2H, Ar-H), 7.17–7.05 (m, 2H, Ar-H), 6.89–6.57 (m, 3H, Ar-H), 6.16 (d, *J* = 7.8 Hz, 1H, Ar-CHNH), 5.67 (d, *J* = 6.1 Hz, 1H, CHNHCO), 5.00–4.90 (m, 1H, Ar-CH-Ar), 4.83 (s, 1H, (CH_3_)_2_CHO), 4.70–4.47 (m, 2H, Ar-CH_2_O), 4.45 (s, 1H, COCHNH), 4.21 (dd, *J* = 6.3, 3.0 Hz, 1H, CH_3_CHCH), 3.86 (dd, *J* = 12.2, 6.5 Hz, 3H, Ar-OCH_3_), 3.77 (t, *J* = 8.5 Hz, 3H, Ar-OCH_3_), 1.26 (dd, *J* = 11.8, 7.7 Hz, 6H, (CH_3_)_2_CHO), 1.18 (d, *J* = 5.8 Hz, 3H, CH_3_CHCH). ^13 ^C NMR (101 MHz, CDCl_3_) δ 170.70, 157.35, 149.16, 148.54, 139.97, 138.44, 133.36, 128.72, 128.54, 127.97, 127.68, 119.65, 111.11, 110.66, 75.01, 71.75, 70.27, 68.91, 59.38, 55.87, 29.72, 22.07. HRMS calcd for C_30_H_35_ClN_2_O_6_ ([M + Na]): 577.2076; Found: 577.2081.

**Data for I-16.** white solid, mp 130 − 132 °C.^1^H NMR (400 MHz, CDCl_3_) δ 7.39 (ddd, *J* = 22.2, 14.3, 7.9 Hz, 2H, Ar-H), 7.32–7.27 (m, 3H, Ar-H), 7.22–7.15 (m, 2H, Ar-H), 7.03 (dd, *J* = 26.1, 8.3 Hz, 2H, Ar-H), 6.81–6.54 (m, 3H, Ar-H), 6.11 (d, *J* = 7.7 Hz, 1H, Ar-CHNH), 5.63 (d, *J* = 6.1 Hz, 1H, CHNHCO), 4.96–4.84 (m, 1H, Ar-CH-Ar), 4.80 (s, 1H, (CH_3_)_2_CHO), 4.55 (dd, *J* = 46.2, 23.3 Hz, 2H, Ar-CH_2_O), 4.40 (s, 1H, COCHNH), 4.24–4.11 (m, 1H, CH_3_CHCH), 3.83 (dd, *J* = 12.1, 6.4 Hz, 3H, Ar-OCH_3_), 3.74 (t, *J* = 8.6 Hz, 3H, Ar-OCH_3_), 1.27–1.19 (m, 6H, (CH_3_)_2_CHO), 1.15 (d, *J* = 5.9 Hz, 3H, CH_3_CHCH). ^13 ^C NMR (101 MHz, CDCl_3_) δ 169.77, 157.19, 149.17, 148.56, 140.49, 138.07, 133.26, 131.68, 129.01, 128.88, 128.51, 121.31, 119.66, 111.13, 75.00, 71.74, 68.90, 57.64, 56.28, 55.87, 22.06, 15.73. HRMS calcd for C_30_H_35_BrN_2_O_6_ ([M + Na]): 621.1571; Found: 621.1574.

**Data for I-17.** white solid, mp 144 − 146 °C. ^1^H NMR (400 MHz, CDCl_3_) δ 7.31–7.27 (m, 5H, Ar-H), 7.20 (dd, *J* = 6.5, 2.9 Hz, 2H, Ar-H), 7.08 (t, *J* = 6.5 Hz, 2H, Ar-H), 6.77–6.62 (m, 3H, Ar-H), 6.13 (t, *J* = 7.1 Hz, 1H, Ar-CHNH), 5.65 (d, *J* = 6.1 Hz, 1H, CHNHCO), 4.95–4.86 (m, 1H, Ar-CH-Ar), 4.64–4.51 (m, 2H, Ar-CH_2_O), 4.40 (s, 1H, COCHNH), 4.18 (dd, *J* = 6.4, 2.9 Hz, 1H, COCHNH), 3.85 (t, *J* = 4.6 Hz, 3H, Ar-OCH_3_), 3.82 (d, *J* = 3.8 Hz, 1H, CH_3_CHCH), 3.73 (d, *J* = 11.8 Hz, 3H, Ar-OCH_3_), 2.32 (d, *J* = 3.2 Hz, 6H, (CH_3_)_2_CHO), 1.26–1.20 (m, 3H, CH_3_CHCH), 1.15 (d, *J* = 5.5 Hz, 3H, Ar-CH_3_). ^13 ^C NMR (101 MHz, CDCl_3_) δ 169.57, 156.84, 149.00, 148.28, 139.34, 137.95, 137.18, 134.08, 129.31, 128.45, 127.80, 127.71, 127.24, 127.13, 119.49, 110.98, 110.55, 75.00, 71.64, 68.80, 57.52, 56.67, 55.89, 22.05, 21.09, 15.51. HRMS calcd for C_31_H_28_N_2_O_6_ ([M + Na]): 557.2622; Found: 557.2628.

**Data for I-18.** white solid, mp 104 − 106 °C. ^1^H NMR (400 MHz, CDCl_3_) δ 7.23–7.10 (m, 2H, Ar-H), 7.04–6.87 (m, 2H, Ar-H), 6.82 (dd, *J* = 16.8, 9.4 Hz, 3H, Ar-H), 5.60 (s, 1H, CHNHCO), 5.06 (s, 1H, Ar-CHCH_3_), 4.89 (d, *J* = 5.4 Hz, 1H, (CH_3_)_2_CHO), 4.63–4.41 (m, 2H, Ar-CH_2_O), 4.31 (s, 1H, COCHNH), 4.20–4.08 (m, 1H, CH_3_CHCH), 3.86 (t, *J* = 13.4 Hz, 6H, CH_3_O), 1.40 (dd, *J* = 14.0, 6.9 Hz, 3H, Ar-CHCH_3_), 1.23 (dd, *J* = 10.5, 5.7 Hz, 6H, (CH_3_)_2_CHO), 1.02 (d, *J* = 6.0 Hz, 3H, CH_3_CHCH). ^13 ^C NMR (101 MHz, CDCl_3_) δ 168.56, 164.21, 160.78, 157.54, 148.90, 138.81, 130.41, 128.26, 120.48, 115.27, 111.34, 110.95, 74.44, 71.56, 68.73, 57.29, 55.88, 55.82, 48.32, 22.08, 15.09. HRMS calcd for C_25_H_33_FN_2_O_6_ ([M + Na]): 499.2215; Found:499.2216.

**Data for I-19.** white solid, mp 124 − 126 °C. ^1^H NMR (400 MHz, CDCl_3_) δ 7.08 (dd, *J* = 25.5, 11.8 Hz, 4H, Ar-H), 6.82 (dd, *J* = 16.0, 7.3 Hz, 3H, Ar-H), 5.63 (s, 1H, CHNHCO), 5.05 (s, 1H, Ar-CHCH_3_), 4.89 (s, 1H, (CH_3_)_2_CHO), 4.52 (dd, *J* = 25.8, 10.1 Hz, 2H, Ar-CH_2_O), 4.31 (s, 1H, COCHNH), 4.13 (s, 1H, CH_3_CHCH), 3.83 (d, *J* = 32.1 Hz, 6H, CH_3_O), 2.31 (s, 3H, Ar-CH_3_), 1.52 − 1.29 (m, 3H, Ar-CHCH_3_), 1.22 (s, 6H, (CH_3_)_2_CHO), 1.11 (d, *J* = 48.5 Hz, 3H, CH_3_CHCH). ^13 ^C NMR (101 MHz, CDCl_3_) δ 168.41, 156.73, 149.00, 148.78, 141.90, 139.99, 136.87, 130.52, 129.31, 125.94, 120.45, 110.97, 74.40, 71.57, 68.72, 57.31, 55.78, 49.96, 48.74, 22.10, 21.05. HRMS calcd for C_26_H_36_N_2_O_6_ ([M + Na]): 495.2466; Found:495.2467.

**Data for I-20.** white solid, mp 110– 112 °C. ^1^H NMR (400 MHz, CDCl_3_) δ 7.11 (s, 2H, Ar-H), 6.80 (d, *J* = 13.7 Hz, 5H, Ar-H), 5.62 (s, 1H, CHNHCO), 5.04 (s, 1H, Ar-CHCH_3_), 4.88 (s, 1H, (CH_3_)_2_CHO), 4.64 (s, 2H, Ar-CH_2_O), 4.49 (d, *J* = 28.3 Hz, 2H, CHCCH_2_O), 4.29 (s, 1H, COCHNH), 4.13 (s, 1H, CH_3_CHCH), 3.82 (d, *J* = 29.3 Hz, 6H, CH_3_O), 2.50 (s, 1H, CHCCH_2_O), 1.41 (s, 3H, Ar-CHCH_3_), 1.22 (s, 6H, (CH_3_)_2_CHO), 1.04 (s, 3H, CH_3_CHCH). ^13 ^C NMR (101 MHz, CDCl_3_) δ 168.41, 156.70, 156.24, 148.94, 135.96, 130.35, 127.28, 127.18, 120.50, 114.90, 111.32, 110.95, 78.53, 75.60, 74.42, 71.53, 68.73, 57.32, 55.92, 55.79, 48.32, 22.09, 21.96, 15.31. HRMS calcd for C_28_H_36_N_2_O_7_ ([M + Na]): 535.2415; Found:535.2413.

**Data for I-21.** white solid, mp 139 − 140 °C. ^1^H NMR (400 MHz, CDCl_3_) δ 7.17 (d, *J* = 8.6 Hz, 1H, Ar-H), 7.11 (d, *J* = 8.4 Hz, 1H, Ar-H), 6.87–6.72 (m, 5H, Ar-H), 5.62 (d, *J* = 5.9 Hz, 1H, CHNHCO), 5.04 (d, *J* = 4.9 Hz, 1H, Ar-CHCH_3_), 4.95–4.82 (m, 1H, (CH_3_)_2_CHO), 4.60–4.41 (m, 2H, Ar-CH_2_O), 4.30 (s, 1H, COCHNH), 4.14 (dd, *J* = 12.1, 5.6 Hz, 1H, CH_3_CHCH), 3.90–3.86 (m, 6H, Ar-OCH_3_), 3.78 (d, *J* = 6.1 Hz, 3H, Ar-OCH_3_), 1.41 (dd, *J* = 13.4, 6.8 Hz, 3H, Ar-CHCH_3_), 1.26–1.20 (m, 6H, (CH_3_)_2_CHO), 1.10 (dd, *J* = 50.9, 6.2 Hz, 3H, CH_3_CHCH). ^13 ^C NMR (101 MHz, CDCl_3_) δ 169.74, 160.35, 149.21, 148.88, 137.18, 130.81, 127.27, 127.16, 120.44, 113.94, 111.34, 110.99, 74.61, 71.55, 69.02, 57.38, 55.90, 55.82, 55.25, 48.86, 22.10, 22.00, 15.56. HRMS calcd for C_28_H_36_N_2_O_7_ ([M + Na]): 535.2415; Found:535.2418.

**Data for I-22.** white solid, mp 152 − 154 °C. ^1^H NMR (400 MHz, CDCl_3_) δ 7.17 (dd, *J* = 43.4, 23.8 Hz, 4H, Ar-H), 6.99 (d, *J* = 21.6 Hz, 4H, Ar-H), 6.74 (s, 1H, Ar-CHNH), 5.60 (s, 1H, CHNHCO), 5.07 (s, 1H, Ar-CHCH_3_), 4.91 (s, 1H, (CH_3_)_2_CHO), 4.59–4.39 (m, 2H, Ar-CH_2_O), 4.30 (s, 1H, COCHNH), 4.18 (s, 1H, CH_3_CHCH), 2.32 (s, 3H, Ar-CH_3_), 1.44 (d, *J* = 6.2 Hz, 3H, Ar-CHCH_3_), 1.23 (s, 6H, (CH_3_)_2_CHO), 1.13 (d, *J* = 47.3 Hz, 3H, CH_3_CHCH). ^13 ^C NMR (101 MHz, CDCl_3_) δ 168.44, 163.60, 161.26, 156.31, 139.90, 137.08, 133.65, 129.45, 126.03, 115.30, 74.66, 70.84, 68.85, 57.62, 48.77, 22.00, 21.01, 15.36. HRMS calcd for C_24_H_31_FN_2_O_4_ ([M + Na]): 453.2160; Found:453.2160.

**Data for I-23.** white solid, mp 130 − 131 °C. ^1^H NMR (400 MHz, CDCl_3_) δ 7.34–7.27 (m, 2H, Ar-H), 7.22–7.00 (m, 4H, Ar-H), 6.80 (t, *J* = 8.5 Hz, 2H, Ar-H), 6.75 (s, 1H, Ar-CHNH), 5.62 (s, 1H, CHNHCO), 5.05 (s, 1H, Ar-CHCH_3_), 4.98–4.81 (m, 1H, (CH_3_)_2_CHO), 4.63 (t, *J* = 12.2 Hz, 2H, Ar-CH_2_O), 4.32 (s, 1H, COCHNH), 4.24–4.07 (m, 1H, CH_3_CHCH), 3.77 (d, *J* = 1.7 Hz, 3H, Ar-OCH_3_), 1.43 (t, *J* = 6.3 Hz, 3H, Ar-CHCH_3_), 1.30–1.21 (m, 6H, (CH_3_)_2_CHO), 1.13 (dd, *J* = 54.8, 6.3 Hz, 3H, CH_3_CHCH). ^13 ^C NMR (101 MHz, CDCl_3_) δ 168.30, 163.19, 158.75, 135.17, 129.83, 127.26, 125.53, 124.20, 115.26, 113.91, 75.11, 68.72, 65.58, 55.25, 48.50, 22.10, 21.81, 15.01. HRMS calcd for C_24_H_31_FN_2_O_5_ ([M + Na]): 469.2109; Found:469.2115.

**Data for I-24.** white solid, mp 145 − 147 °C. ^1^H NMR (400 MHz, CDCl_3_) δ 7.17 (dd, *J* = 17.9, 7.6 Hz, 4H, Ar-H), 7.06–6.80 (m, 4H, Ar-H), 6.70 (s, 1H, Ar-CHNH), 5.59 (d, *J* = 10.7 Hz, 1H, CHNHCO), 5.07 (s, 1H, Ar-CHCH_3_), 4.90 (s, 1H, (CH_3_)_2_CHO), 4.67 (s, 2H, Ar-CH_2_O), 4.50 (d, *J* = 28.3 Hz, 2H, CHCCH_2_O), 4.29 (s, 1H, COCHNH), 4.14 (d, *J* = 20.9 Hz, 1H, CH_3_CHCH), 2.51 (s, 1H, CHCCH_2_O), 1.46–1.38 (m, 3H, Ar-CHCH_3_), 1.23 (s, 6H, (CH_3_)_2_CHO), 1.13 (d, *J* = 48.8 Hz, 3H, CH_3_CHCH). HRMS calcd for C_26_H_31_FN_2_O_5_ ([M + Na]): 493.2109; Found:493.2114.

**Data for I-25.** white solid, mp 135 − 137 °C. ^1^H NMR (400 MHz, CDCl_3_) δ 7.18 (s, 2H, Ar-H), 6.96 (s, 2H, Ar-H), 6.84 − 6.68 (m, 3H, Ar-H), 5.59 (s, 1H, CHNHCO), 5.04 (s, 1H, Ar-CHCH_3_), 4.89 (s, 1H, (CH_3_)_2_CHO), 4.50 (d, *J* = 25.1 Hz, 2H, Ar-CH_2_O), 4.29 (s, 1H, COCHNH), 4.15 (s, 1H, CH_3_CHCH), 3.89–3.74 (m, 6H, Ar-OCH_3_), 1.44 (d, *J* = 6.3 Hz, 3H, Ar-CHCH_3_), 1.23 (s, 6H, (CH_3_)_2_CHO), 1.13 (d, *J* = 42.4 Hz, 3H, CH_3_CHCH). ^13 ^C NMR (101 MHz, CDCl_3_) δ 168.41, 165.59, 161.97, 156.81, 149.57, 148.31, 135.47, 134.44, 129.47, 117.85, 115.19, 109.73, 74.66, 70.82, 68.84, 57.56, 55.86, 48.72, 29.70, 21.92, 15.30. HRMS calcd for C_25_H_33_FN_2_O_6_ ([M + Na]): 499.2215; Found:499.2215.

**Data for I-26.** white solid, mp 133 − 135 °C. ^1^H NMR (400 MHz, CDCl_3_) δ 7.19 (s, 1H, Ar-H), 6.98 (s, 3H, Ar-H), 6.78 (s, 2H, Ar-H), 5.60 (s, 1H, CHNHCO), 5.06 (s, 1H, Ar-CHCH_3_), 4.89 (s, 1H, (CH_3_)_2_CHO), 4.74 (s, 2H, Ar-CH_2_O), 4.53 (s, 2H, CHCCH_2_O), 4.30 (s, 1H, COCHNH), 4.15 (s, 1H, CH_3_CHCH), 3.80 (s, 3H, Ar-OCH_3_), 2.50 (s, 1H, CHCCH_2_O), 1.44 (s, 3H, Ar-CHCH_3_), 1.23 (s, 6H, (CH_3_)_2_CHO), 1.09 (s, 3H, CH_3_CHCH). ^13 ^C NMR (101 MHz, CDCl_3_) δ 168.46, 164.55, 162.77, 156.89, 149.65, 147.12, 136.88, 134.98, 129.47, 117.69, 114.17, 110.28, 78.53, 75.87, 74.64, 70.82, 68.85, 57.58, 56.69, 55.84, 48.75, 22.07, 21.79, 15.30. HRMS calcd for C_27_H_33_FN_2_O_6_ ([M + Na]): 523.2215; Found:523.2217.

**Data for I-27.** white solid, mp 125 − 127 °C. ^1^H NMR (400 MHz, CDCl_3_) δ 7.29 (s, 2H, Ar-H), 7.18 (s, 2H, Ar-H), 6.96 (dd, *J* = 22.6, 7.8 Hz, 1H, Ar-CHNH), 6.89–6.63 (m, 3H, Ar-H), 5.61 (s, 1H, CHNHCO), 5.09 (d, *J* = 5.4 Hz, 1H, Ar-CHCH_3_), 4.92 (d, *J* = 6.0 Hz, 1H, (CH_3_)_2_CHO), 4.77 (s, 2H, Ar-CH_2_O), 4.54 (d, *J* = 25.5 Hz, 2H, CHCCH_2_O), 4.32 (s, 1H, COCHNH), 4.18 (s, 1H, CH_3_CHCH), 3.83 (d, *J* = 11.6 Hz, 3H, Ar-OCH_3_), 2.53 (s, 1H, CHCCH_2_O), 1.62–1.39 (m, 3H, Ar-CHCH_3_), 1.26 (s,6H, (CH_3_)_2_CHO), 1.12 (s, 3H, CH_3_CHCH). ^13 ^C NMR (101 MHz, CDCl_3_) δ 170.95, 157.95, 149.66, 147.10, 136.85, 134.41, 128.81, 117.78, 114.13, 110.28, 101.68, 78.55, 75.88, 74.78, 70.78, 69.49, 58.03, 56.70, 55.87, 48.64, 22.09, 21.82, 15.67. HRMS calcd for C_27_H_33_ClN_2_O_6_ ([M + Na]): 539.1919; Found:539.1921.

**Data for I-28.** white solid, mp 137 − 139 °C. ^1^H NMR (400 MHz, CDCl_3_) δ 7.50–7.15 (m, 5H, Ar-H), 7.00–6.71 (m, 3H, Ar-H), 5.76 (d, *J* = 28.0 Hz, 1H, CHNHCO), 5.15 (s, 1H, Ar-CHCH_3_), 4.99 (s, 1H, (CH_3_)_2_CHO), 4.54 (dd, *J* = 31.5, 18.0 Hz, 2H, Ar-CH_2_O), 4.41 (s, 1H, COCHNH), 4.23 (d, *J* = 30.6 Hz, 1H, CH_3_CHCH), 3.87 (s, 3H, Ar-OCH_3_), 1.52 (s, 3H, Ar-CHCH_3_), 1.32 (s, 6H, (CH_3_)_2_CHO), 1.16 (s, 3H, CH_3_CHCH). ^13 ^C NMR (101 MHz, CDCl_3_) δ 175.24, 168.55, 158.85, 156.28, 136.45, 134.87, 133.54, 128.69, 127.30, 113.97, 74.95, 70.48, 68.84, 55.26, 48.52, 21.92, 21.79, 16.24. HRMS calcd for C_24_H_31_ClN_2_O_5_ ([M + Na]): 485.1814; Found:485.1813.

**Data for I-29.** white solid, mp 149 − 151 °C.^1^H NMR (400 MHz, CDCl_3_) δ 7.25–7.16 (m, 3H, Ar-H), 7.09 (dt, *J* = 30.1, 10.4 Hz, 3H, Ar-H), 6.82 (dd, *J* = 18.1, 8.2 Hz, 2H, Ar-H), 6.62 (t, *J* = 7.8 Hz, 1H, Ar-CHNH), 5.60–5.46 (m, 1H, CHNHCO), 5.00 (s, 1H, Ar-CHCH_3_), 4.83 (dd, *J* = 12.1, 5.9 Hz, 1H, (CH_3_)_2_CHO), 4.61 (s, 2H, Ar-CH_2_O), 4.41 (dt, *J* = 26.8, 12.9 Hz, 2H, CHCCH_2_O), 4.22 (s, 1H, COCHNH), 4.15–4.01 (m, 1H, CH_3_CHCH), 2.45 (s, 1H, CHCCH_2_O), 1.36 (t, *J* = 7.5 Hz, 3H, Ar-CHCH_3_), 1.20–1.14 (m, 6H, (CH_3_)_2_CHO), 1.05 (dd, *J* = 46.7, 6.0 Hz, 3H, C**H**_3_CHCH). HRMS calcd for C_26_H_31_ClN_2_O_5_ ([M + Na]): 509.1814; Found:509.2062.

**Data for I-30.** white solid, mp 136 − 138 °C. ^1^H NMR (400 MHz, CDCl_3_) δ 7.17 (s, 4H, Ar-H), 6.91–6.75 (m, 4H, Ar-H), 5.68 (s, 1H, CHNHCO), 5.05 (s, 1H, Ar-CHCH_3_), 4.89 (s, 1H, (CH_3_)_2_CHO), 4.46 (dt, *J* = 31.3, 10.1 Hz, 2H, Ar-CH_2_O), 4.31 (s, 1H, COCHNH), 4.12 (d, *J* = 21.3 Hz, 1H, CH_3_CHCH), 3.78 (d, *J* = 7.1 Hz, 6H, Ar-CH_2_O), 1.40 (d, *J* = 6.5 Hz, 3H, Ar-CHCH_3_), 1.22 (s, 6H, (CH_3_)_2_CHO), 1.04 (s, 3H, CH_3_CHCH). ^13 ^C NMR (101 MHz, CDCl_3_) δ 173.77, 168.61, 159.05, 156.40, 135.05, 130.34, 128.62, 127.30, 113.89, 71.24, 68.69, 57.47, 55.24, 48.47, 22.07, 21.88, 16.35. HRMS calcd for C_26_H_31_ClN_2_O_5_ ([M + Na]): 509.1814; Found:509.1808.

**Data for I-31.** white solid, mp 119 − 120 °C. ^1^H NMR (400 MHz, CDCl_3_) δ 7.18 (dd, *J* = 21.1, 8.9 Hz, 4H, Ar-H), 6.88 (dt, *J* = 34.8, 17.3 Hz, 4H, Ar-H), 5.67 (s, 1H, CHNHCO), 5.07 (s, 1H, Ar-CHCH_3_), 4.91 (d, *J* = 5.2 Hz, 1H, (CH_3_)_2_CHO), 4.67 (d, *J* = 3.7 Hz, 2H, Ar-CH_2_O), 4.61–4.37 (m, 2H, CHCCH_2_O), 4.32 (s, 1H, COCHNH), 4.14 (d, *J* = 17.6 Hz, 1H, CH_3_CHCH), 3.82 (s, 3H, Ar-OCH_3_), 2.54 (s, 1H, CHCCH_2_O), 1.47–1.39 (m, 3H, Ar-CHCH_3_), 1.25 (s, 6H, (CH_3_)_2_CHO), 1.13 (dd, *J* = 50.9, 5.2 Hz, 3H, CH_3_CHCH). HRMS calcd for C_27_H_34_N_2_O_6_ ([M + Na]): 505.2309; Found:505.2304.

**Data for I-32.** white solid, mp 95 − 96 °C. ^1^H NMR (400 MHz, CDCl_3_) δ 7.19 (s, 2H, Ar-H), 6.81 (d, *J* = 15.1 Hz, 5H, Ar-H), 5.65 (s, 1H, CHNHCO), 5.07 (s, 1H, Ar-CHCH_3_), 4.91 (s, 1H, (CH_3_)_2_CHO), 4.75 (s, 2H, Ar-CH_2_O), 4.52 (d, *J* = 20.3 Hz, 2H, CHCCH_2_O), 4.33 (s, 1H, COCHNH), 4.15 (s, 1H, CH_3_CHCH), 3.82 (s, 6H, Ar-OCH_3_), 2.52 (s, 1H, CHCCH_2_O), 1.46 (s, 3H, Ar-CHCH_3_), 1.25 (s, 6H, (CH_3_)_2_CHO), 1.09 (s, 3H, CH_3_CHCH). HRMS calcd for C_28_H_36_N_2_O_7_ ([M + Na]): 535.2415; Found: 535.2413.

**Data for I-33.** white solid, mp 142 − 144 °C. ^1^H NMR (400 MHz, CDCl_3_) δ 7.35–7.08 (m, 6H, Ar-H), 6.92–6.76 (m, 2H, Ar-H), 6.74 (d, J = 7.1 Hz, 1H, Ar-CHNH), 5.62 (s, 1H, CHNHCO), 5.17–4.98 (m, 1H, Ar-CHCH_3_), 4.90 (dd, *J* = 12.1, 5.9 Hz, 1H, (CH_3_)_2_CHO), 4.54 (dd, *J* = 23.7, 11.9 Hz, 2H, Ar-CH_2_O), 4.30 (s, 1H, COCHNH), 4.14 (d, *J* = 7.8 Hz, 1H, CH_3_CHCH), 3.79 (t, *J* = 5.5 Hz, 3H, Ar-OCH_3_), 2.35 (d, *J* = 1.1 Hz, 3H, Ar-CH_3_), 1.42 (dd, *J* = 10.4, 6.9 Hz, 3H, Ar-CHCH_3_), 1.27–1.19 (m, 6H, (CH_3_)_2_CHO), 1.11 (dd, *J* = 52.3, 6.2 Hz, 3H, CH_3_CHCH). ^13 ^C NMR (101 MHz, CDCl_3_) δ 168.45, 158.76, 156.88, 137.57, 135.04, 129.17, 127.30, 126.68, 113.91, 71.48, 68.67, 57.39, 55.27, 48.46, 22.04, 21.22, 15.00. HRMS calcd for C_25_H_34_N_2_O_5_ ([M + Na]): 465.2360; Found:465.2363.

**Data for I-34.** white solid, mp 126 − 128 °C. ^1^H NMR (400 MHz, CDCl_3_) δ 7.19 (s, 3H, Ar-H), 6.81 (d, *J* = 15.1 Hz, 5H, Ar-H), 5.65 (s, 1H, CHNHCO), 5.07 (s, 1H, Ar-CHCH_3_), 4.91 (s, 1H, (CH_3_)_2_CHO), 4.75 (s, 2H, Ar-CH_2_O), 4.52 (d, *J* = 20.3 Hz, 2H, CHCCH_2_O), 4.33 (s, 1H, COCHNH), 4.15 (s, 1H, CH_3_CHCH), 3.82 (s, 3H), 2.52 (s, 1H, CHCCH_2_O), 1.46 (s, 3H, Ar-CHCH_3_), 1.25 (s, 6H, (CH_3_)_2_CHO), 1.09 (s, 3H, CH_3_CHCH). HRMS calcd for C_27_H_34_N_2_O_5_ ([M + Na]): 489.2360; Found:489.2365.

**Data for I-35.** white solid, mp 128 − 130 °C.^1^H NMR (400 MHz, CDCl_3_) δ 7.12 (s, 4H, Ar-H), 6.87 (d, *J* = 6.7 Hz, 1H, Ar-H), 6.77 (d, *J* = 18.2 Hz, 2H, Ar-H), 5.73 (s, 1H, CHNHCO), 5.06 (s, 1H, Ar-CHCH_3_), 4.92 (dd, *J* = 27.9, 13.9 Hz, 1H, (CH_3_)_2_CHO), 4.55 (dd, *J* = 13.9, 8.7 Hz, 2H, Ar-CH_2_O), 4.35 (s, 1H, COCHNH), 4.13 (d, *J* = 14.6 Hz, 1H, CH_3_CHCH), 3.81 (dd, *J* = 21.7, 8.6 Hz, 6H, Ar-OCH_3_), 2.32 (s, 3H, Ar-CH_3_), 1.53–1.32 (m, 3H, Ar-CHCH_3_), 1.21 (d, *J* = 15.2 Hz, 6H, (CH_3_)_2_CHO), 1.12 (d, *J* = 28.0 Hz, 3H, CH_3_CHCH). ^13 ^C NMR (101 MHz, CDCl_3_) δ 172.32, 169.02, 157.56, 156.39, 148.98, 148.29, 137.74, 135.75, 131.98, 118.01, 111.16, 109.73, 73.19, 68.96, 61.92, 55.84, 48.85, 46.60, 21.93, 21.10, 15.37. HRMS calcd for C_26_H_36_N_2_O_6_([M + Na]): 495.2466; Found:495.2463.

**Data for I-36.** white solid, mp 118 − 120 °C. ^1^H NMR (400 MHz, CDCl_3_) δ 7.13 (s, 4H, Ar-H), 6.81 (s, 3H, Ar-H), 5.70 (s, 1H, CHNHCO), 5.06 (s, 1H, Ar-CHCH_3_), 4.90 (s, 1H, (CH_3_)_2_CHO), 4.73 (s, 2H, Ar-CH_2_O), 4.55 (s, 2H, CHCCH_2_O), 4.34 (s, 1H, COCHNH), 4.12 (s, 1H, Ar-CHCH_3_), 3.77 (s, 3H, Ar-OCH_3_), 2.49 (s, 1H, CHCCH_2_O), 2.33 (s, 3H, Ar-CH_3_), 1.41 (d, *J* = 23.5 Hz, 3H, Ar-CHCH_3_), 1.23 (s, 6H, (CH_3_)_2_CHO), 1.09 (s, 3H, CH_3_CHCH). ^13 ^C NMR (101 MHz, CDCl_3_) δ 172.03, 169.56, 156.64, 150.15, 146.40, 137.09, 135.58, 132.28, 117.78, 114.20, 110.19, 78.57, 73.11, 69.58, 61.87, 57.53, 56.71, 55.81, 48.73, 46.61, 22.06, 21.03, 15.32. HRMS calcd for C_28_H_36_N_2_O_6_([M + Na]): 519.2466; Found:519.2466.

### Fungicidal activity

Detailed fungicidal activity test method were according to our previous work^40–43^. According to statistical requirements, each fungicidal activity was repeated at least three times.

## Conclusions

In summary, a series of new CAA analogues were designed and synthesised using pharmacophore model. Their structures were determined by the spectra analysis, and their fungicidal activities were assayed *in vitro* and *in vivo*. From the bioassay results, some of target compounds exhibited more potent fungicidal activity against *Oomycete* fungi *P. capsici in vitro*. Interestingly, compound **I-1, I-2, I-3, I-6** and **I-7** exhibited moderate control effect (>50%) against *P. cubensis* in greenhouse at 6.25 μg/mL. Furthermore, further structure expeditions are undergoing using pharmacophore model and will be reported in the near future.

## Supplementary Material

Supplemental Material
