# Supplementary material for: Design, synthesis and antifungal activity of threoninamide carbamate derivatives via pharmacophore model
Source: J Enzyme Inhib Med Chem. 2020 Mar 9;35(1):682–91. doi: 10.1080/14756366.2020.1729144 (PMC7144198; doi:10.1080/14756366.2020.1729144)
Supplement: Supplemental Material [file IENZ_A_1729144_SM7037.pdf]

Supplemental Materials

Design, Synthesis and Antifungal Activity ofThreoninamide Carbamate Derivatives Via Pharmacophore Model

Xiu-Jiang Du[a], Xing-JiePeng[a], Rui-QiZhao[a], Wei-Guang Zhao[a]*, Wei-Li Dong[b]*, Xing-Hai Liu[c]*

a. State Key Laboratory of Elemental Organic Chemistry, Nankai University, Tianjin, 310074, China

b.Tianjin Key Laboratory on Technologies Enabling Development of Clinical Therapeutics and Diagnostics (T heranostics), School of Pharmacy, Tianjin Medical University, Tianjin, China

c. College of Chemical Engineering, Zhejiang University of Technology, Hangzhou 310014, China

Table of contents

1. The chemical data of target compounds……….................... 2-16

I-1

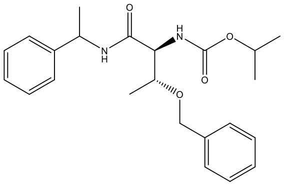

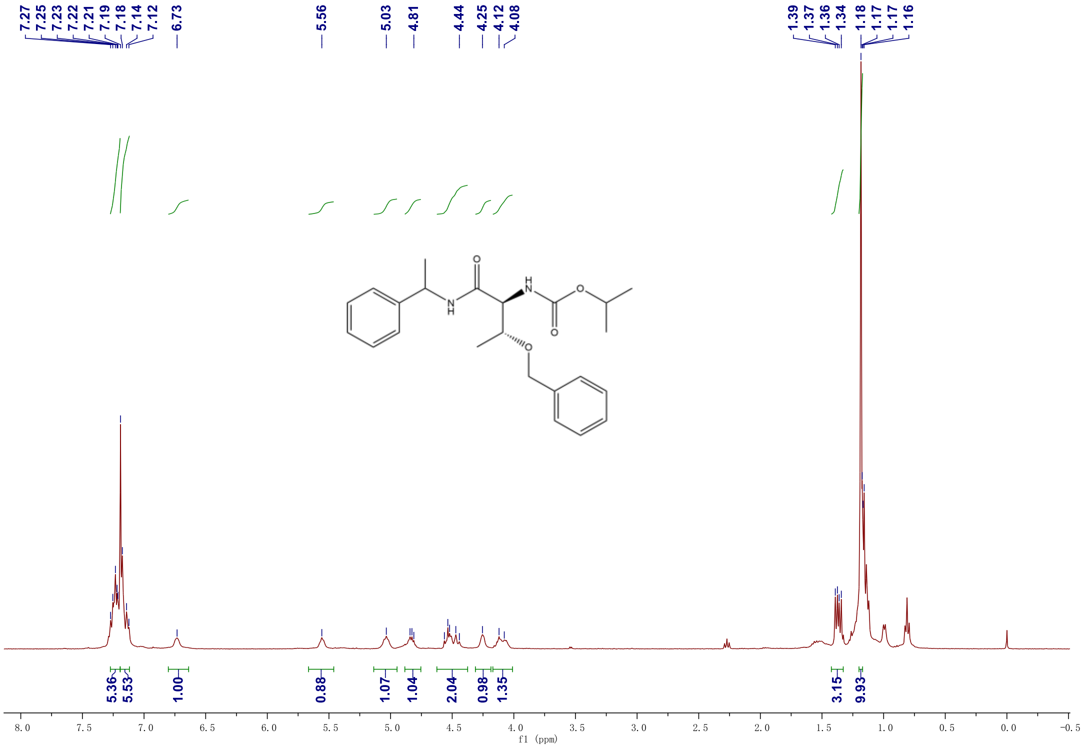

Figure 1 ^{1}HNMR spectrum of **I-1**

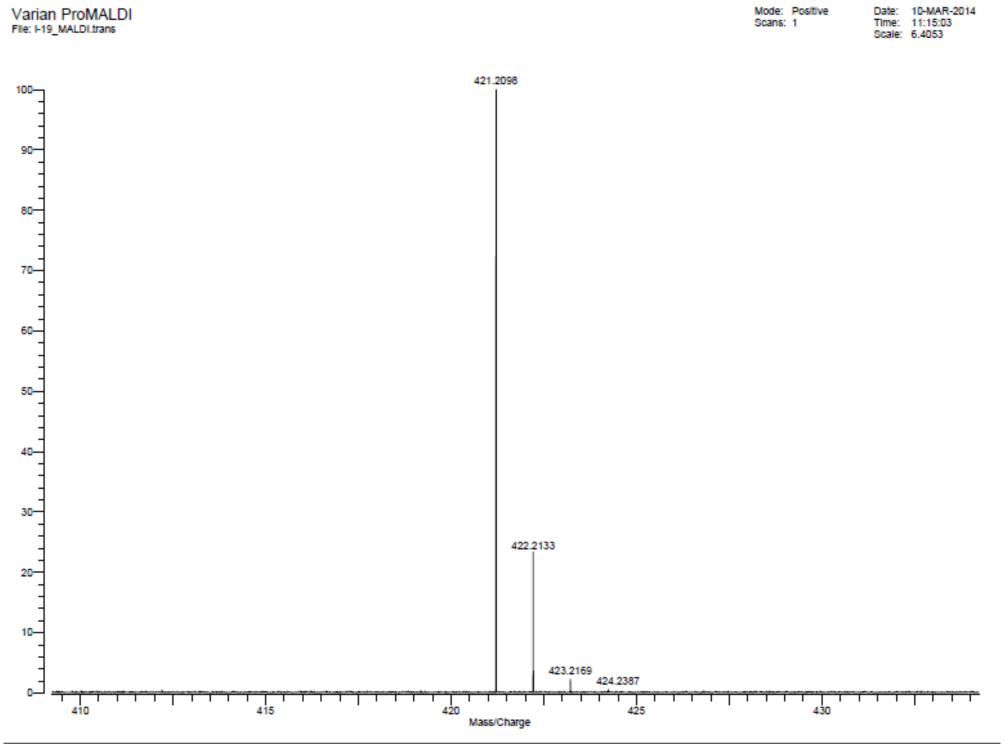

Figure 2 HRMS spectrum of **I-1**

I-2

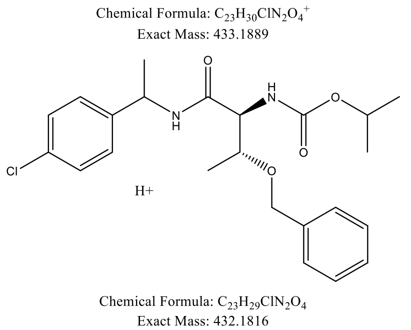

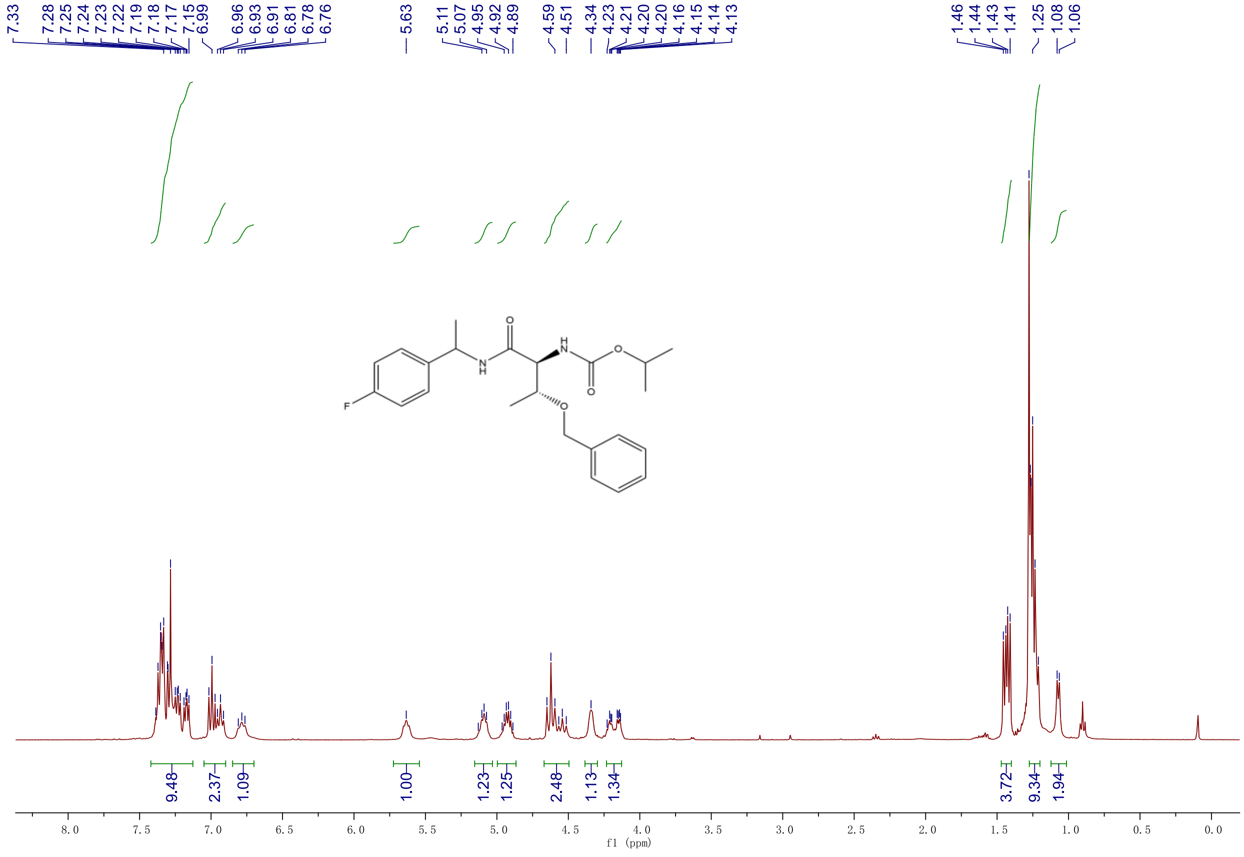

Figure 3 ^{1}HNMR spectrum of **I-2**

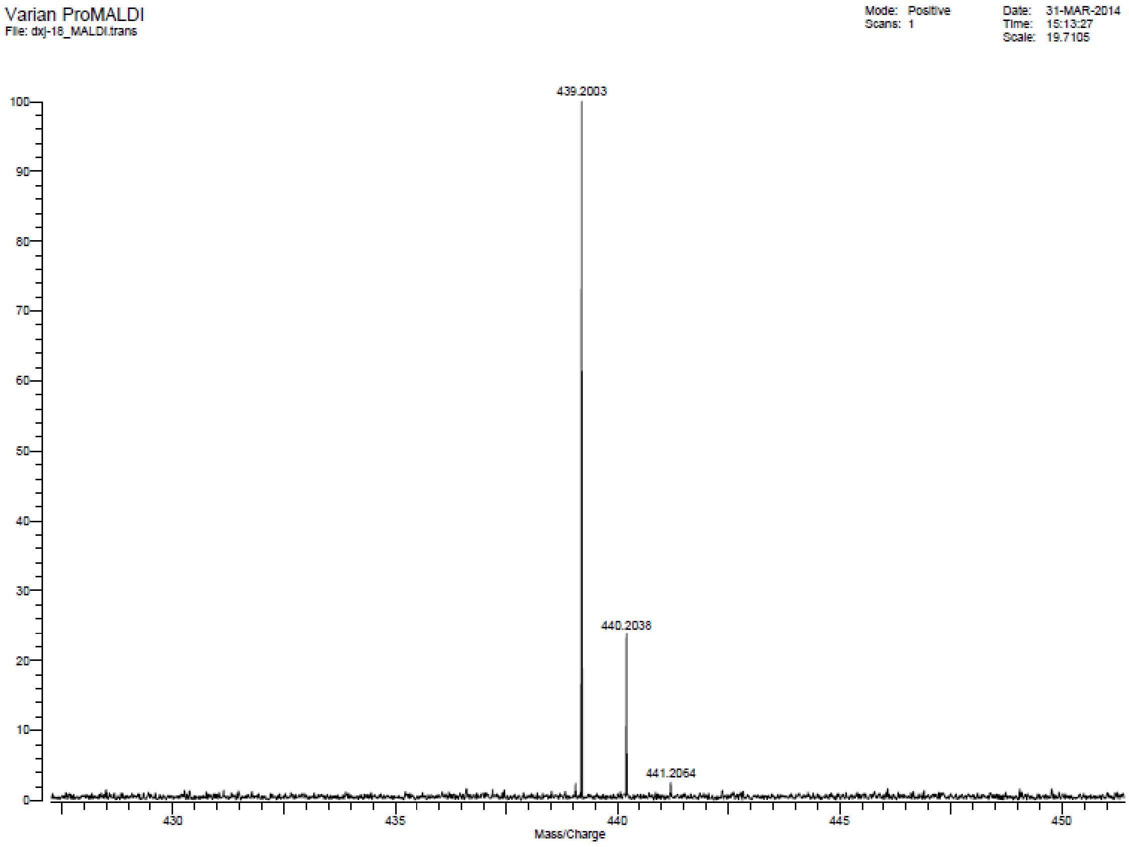

Figure 4 HRMS spectrum of **I-2**

I-3

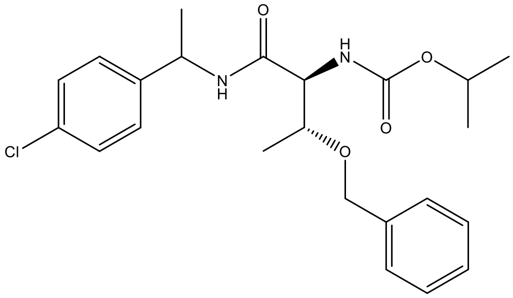

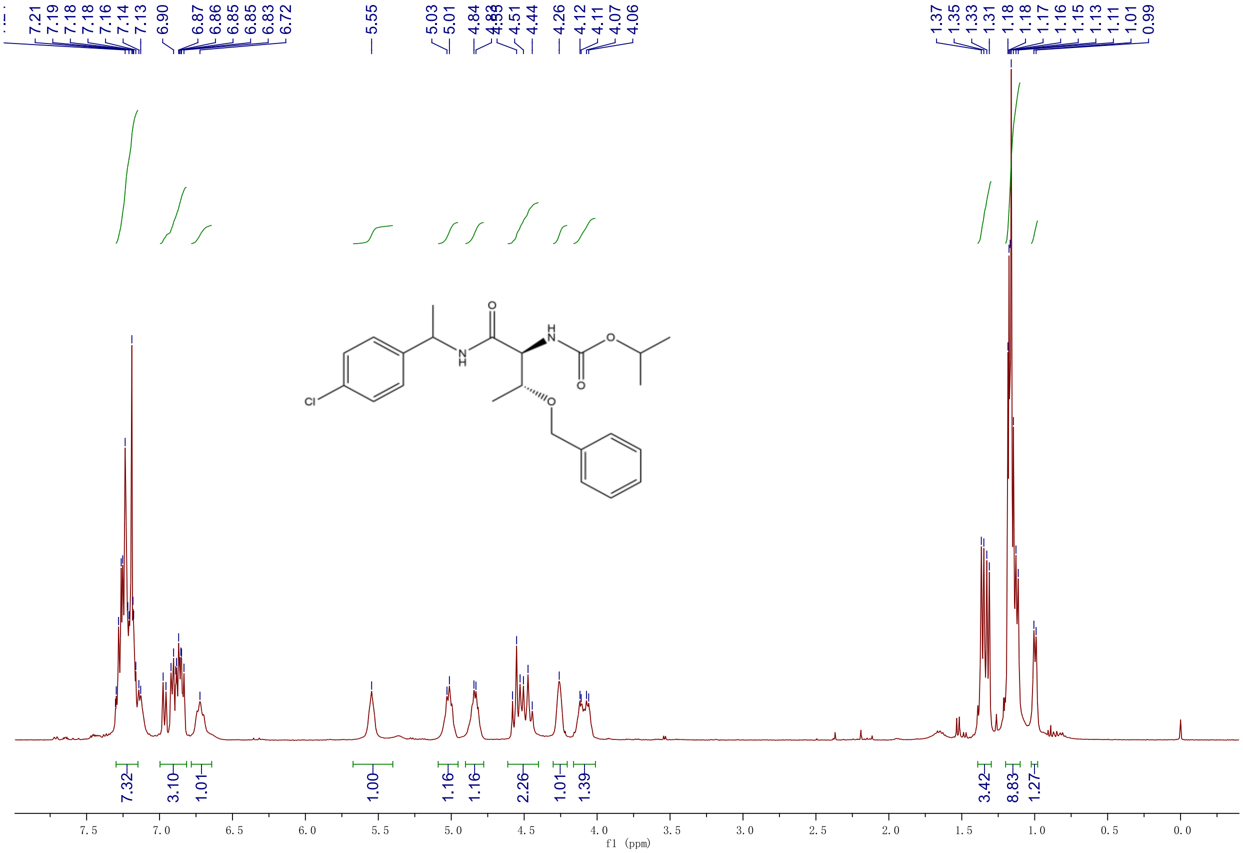

Figure 5 ^{1}HNMR spectrum of **I-3**

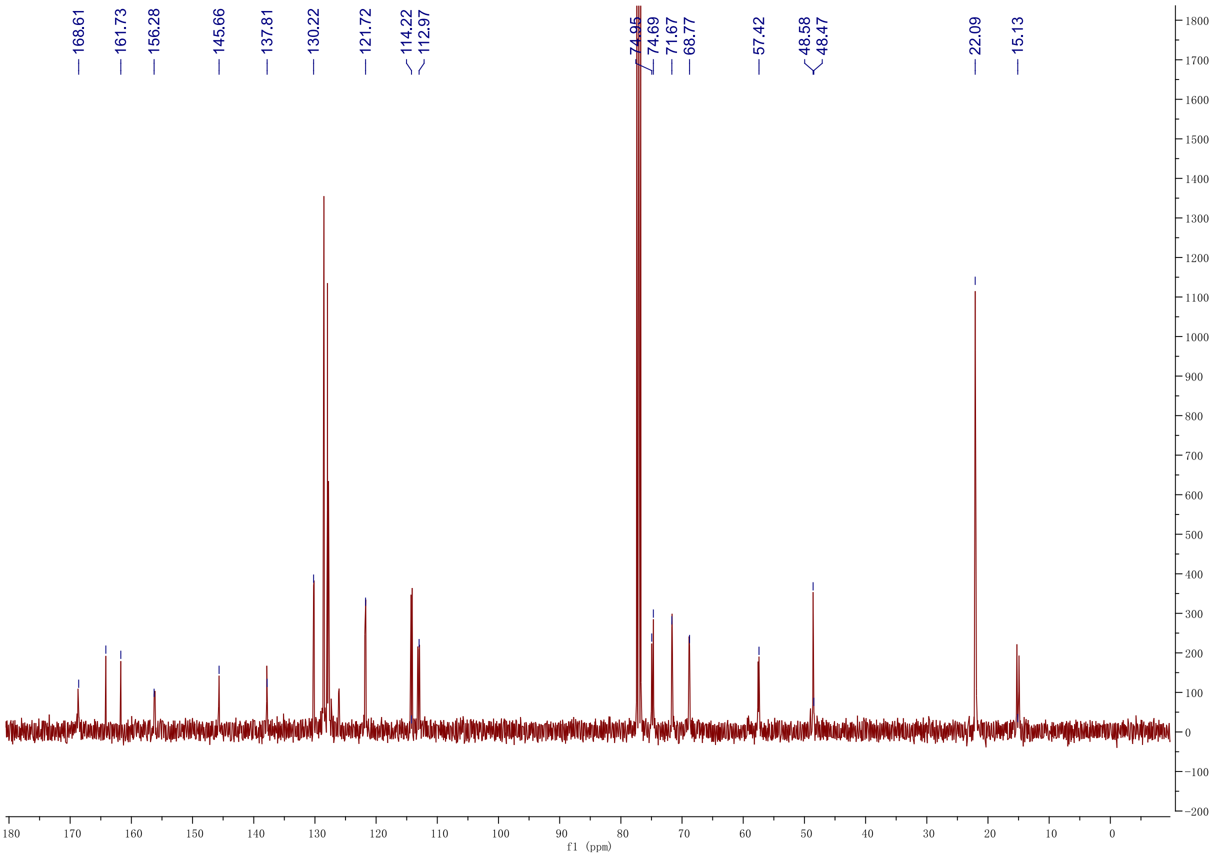

Figure 6 ^{13}C NMR spectrum of **I-3**

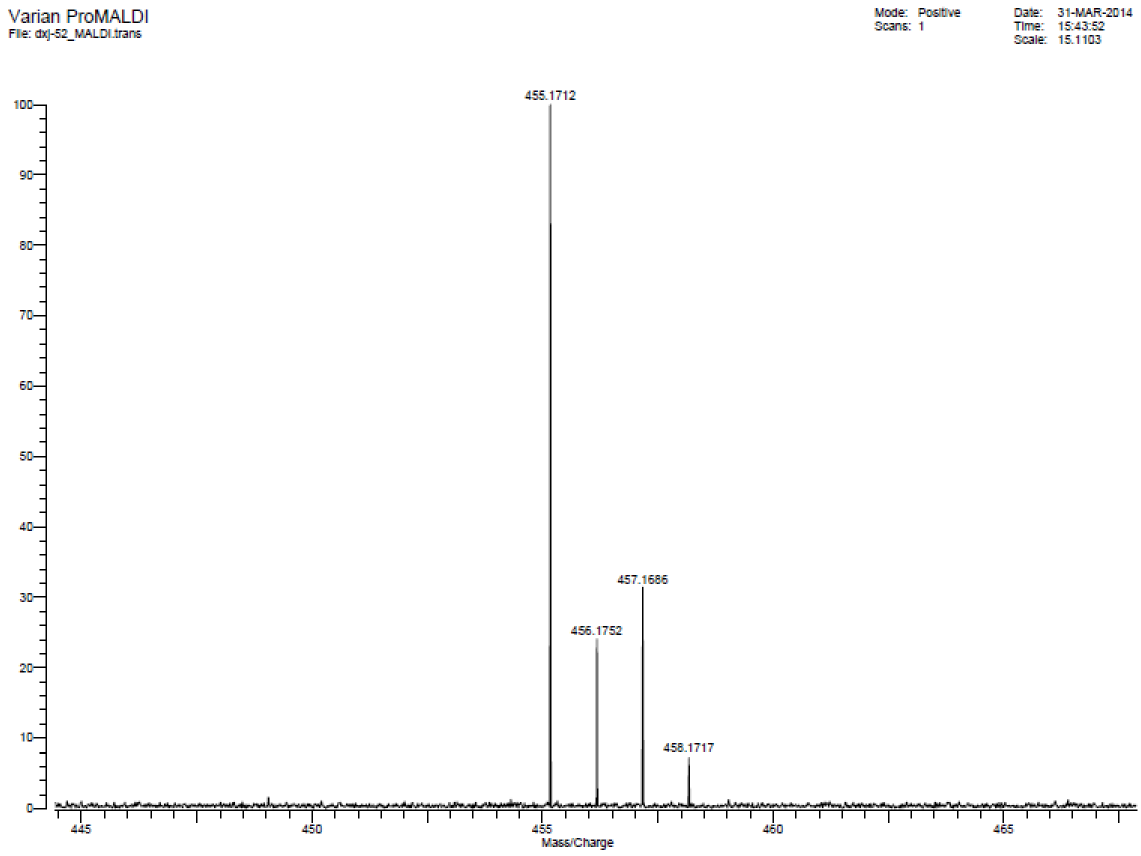

Figure 7 HRMS spectrum of **I-3**

I-4

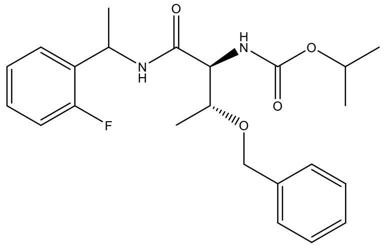

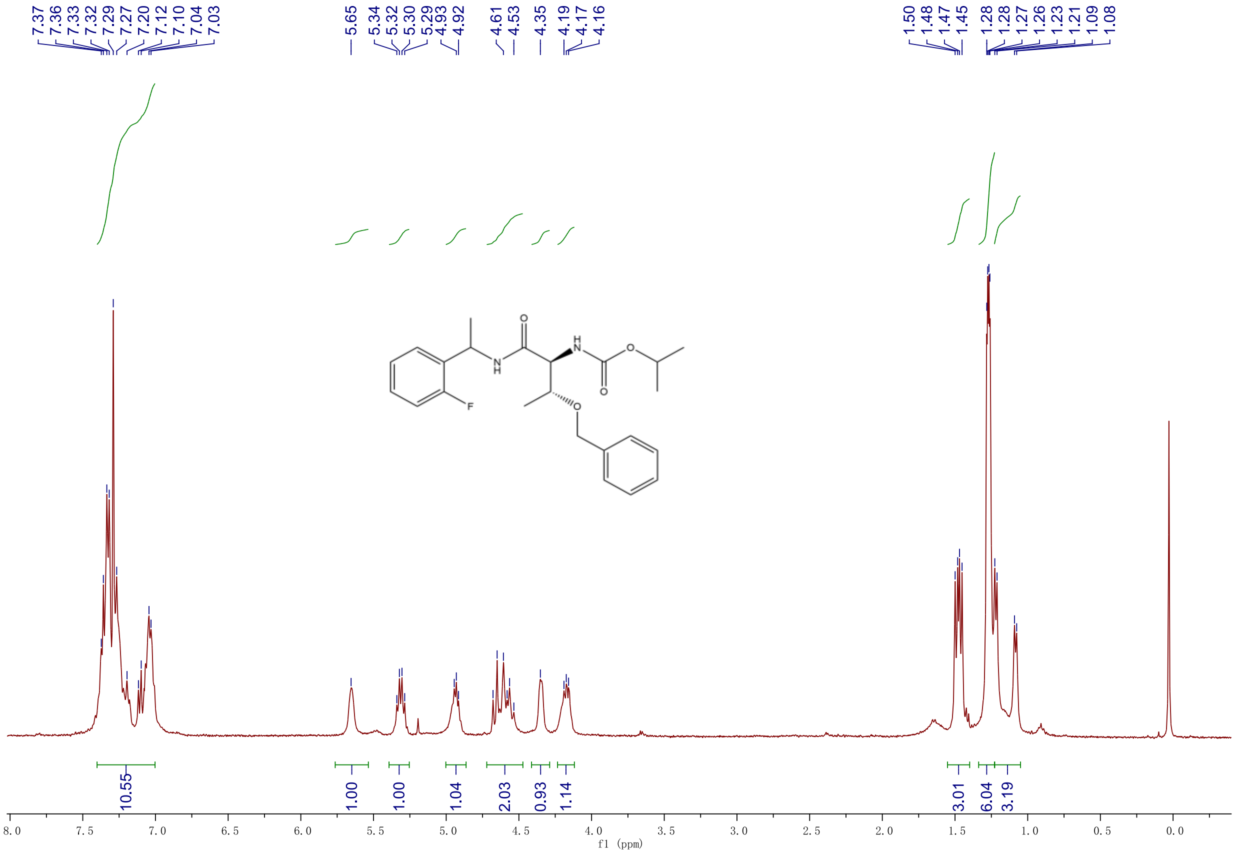

Figure 8 ^{1}HNMR spectrum of **I-4**

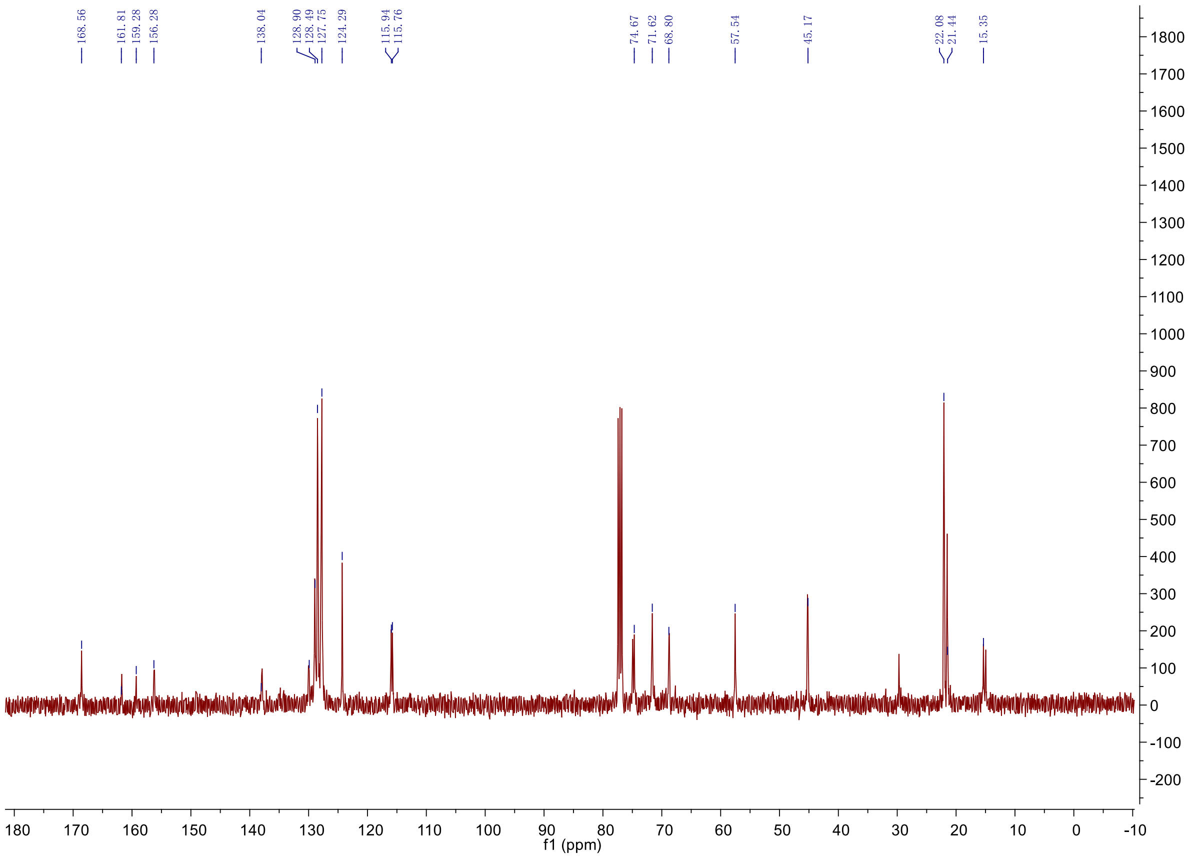

Figure 9 ^{13}C NMR spectrum of **I-4**

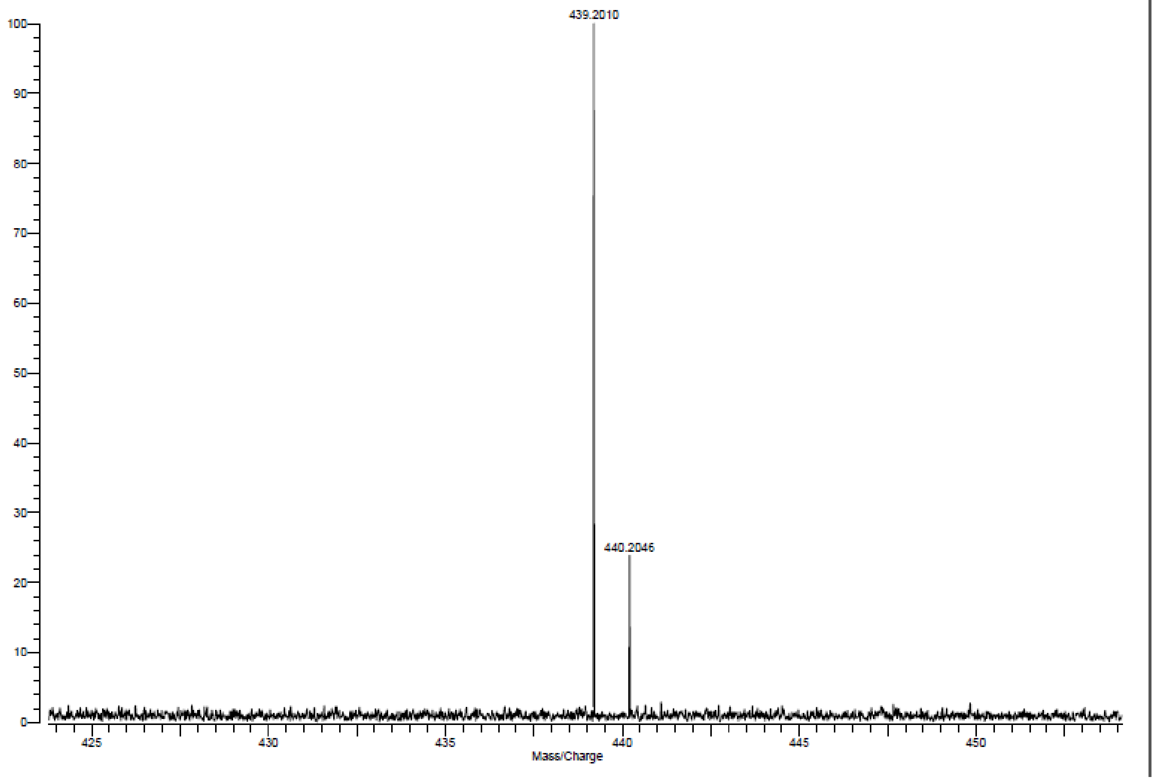

Figure 10 HRMS spectrum of **I-4**

I-5

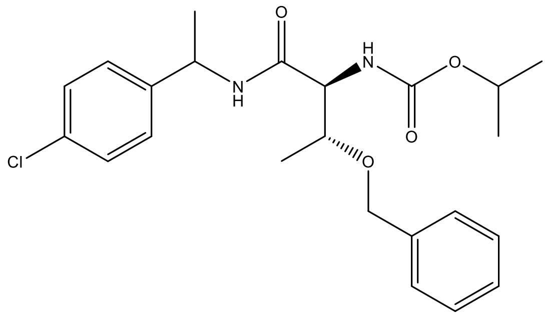

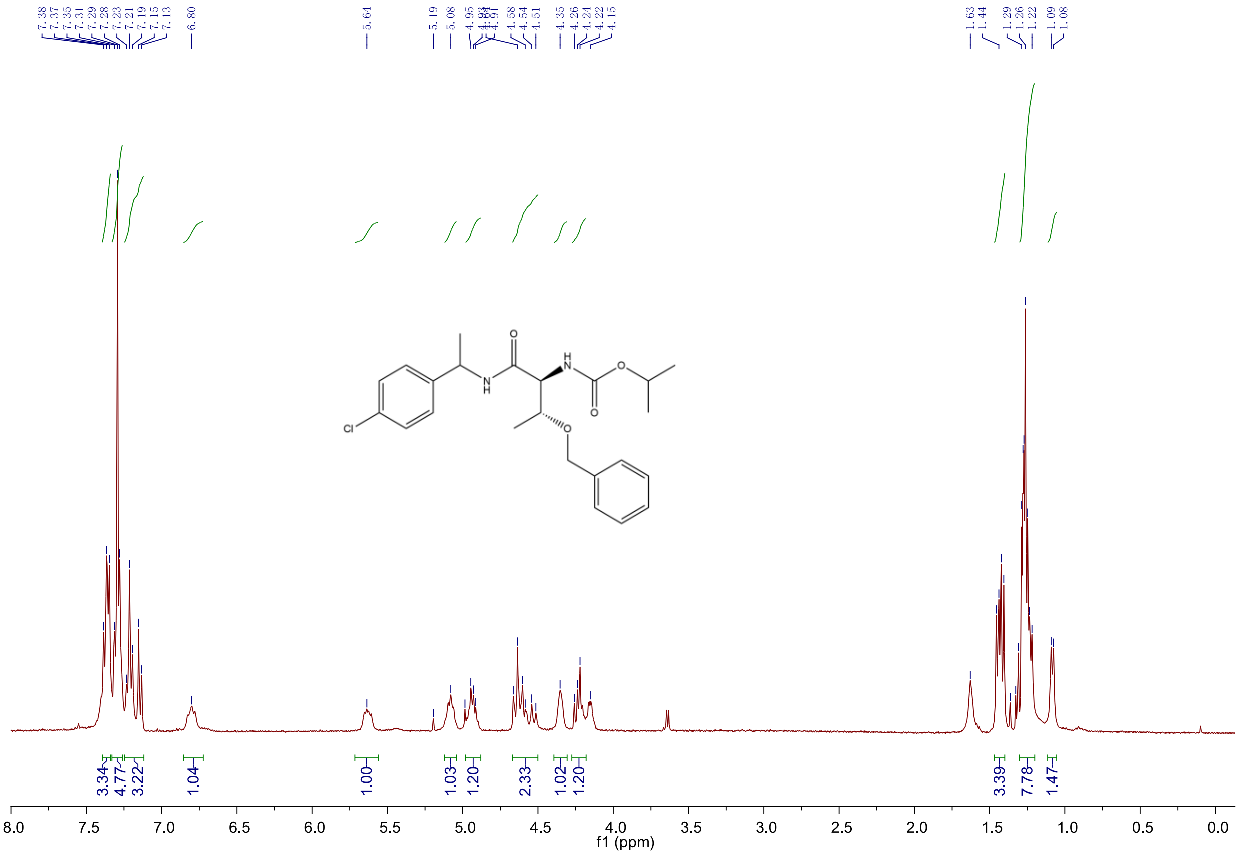

Figure 11 ^{1}HNMR spectrum of **I-5**

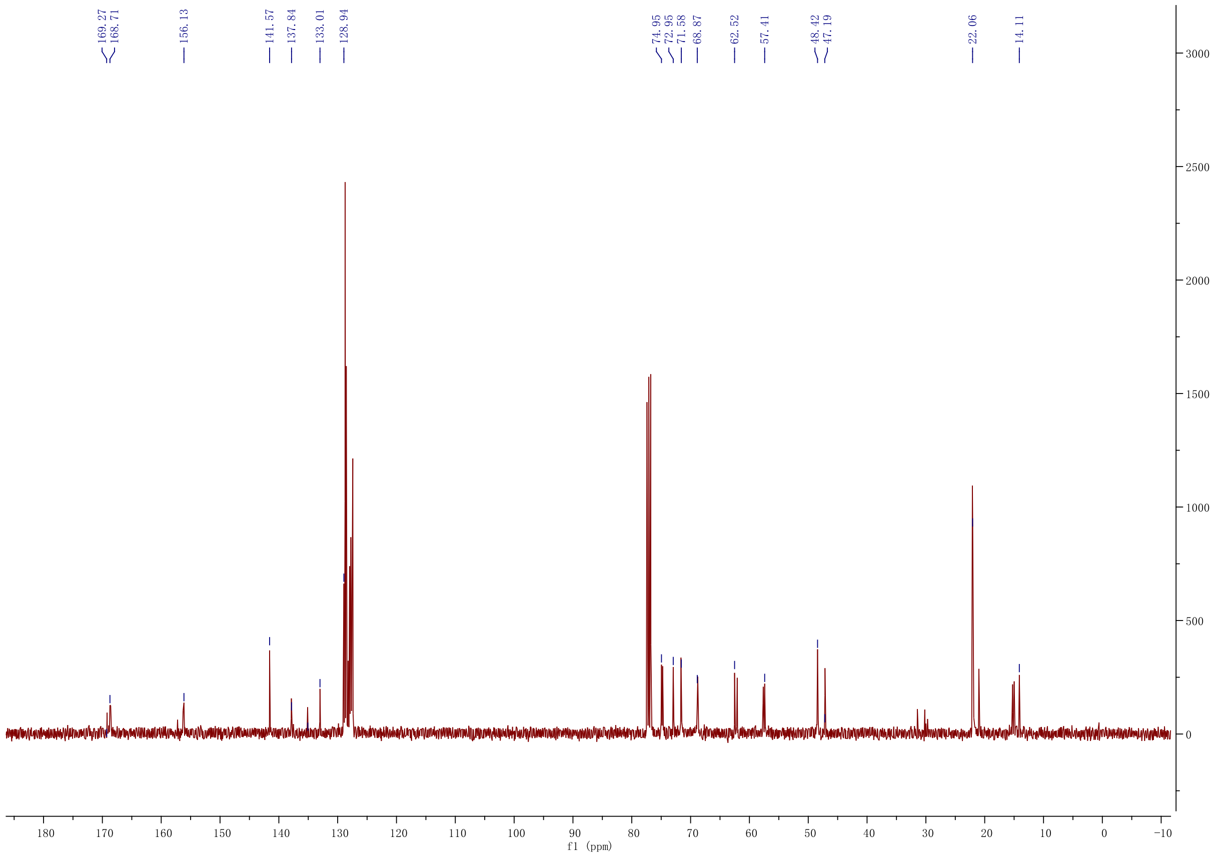

Figure 12 ^{13}C NMR spectrum of **I-5**

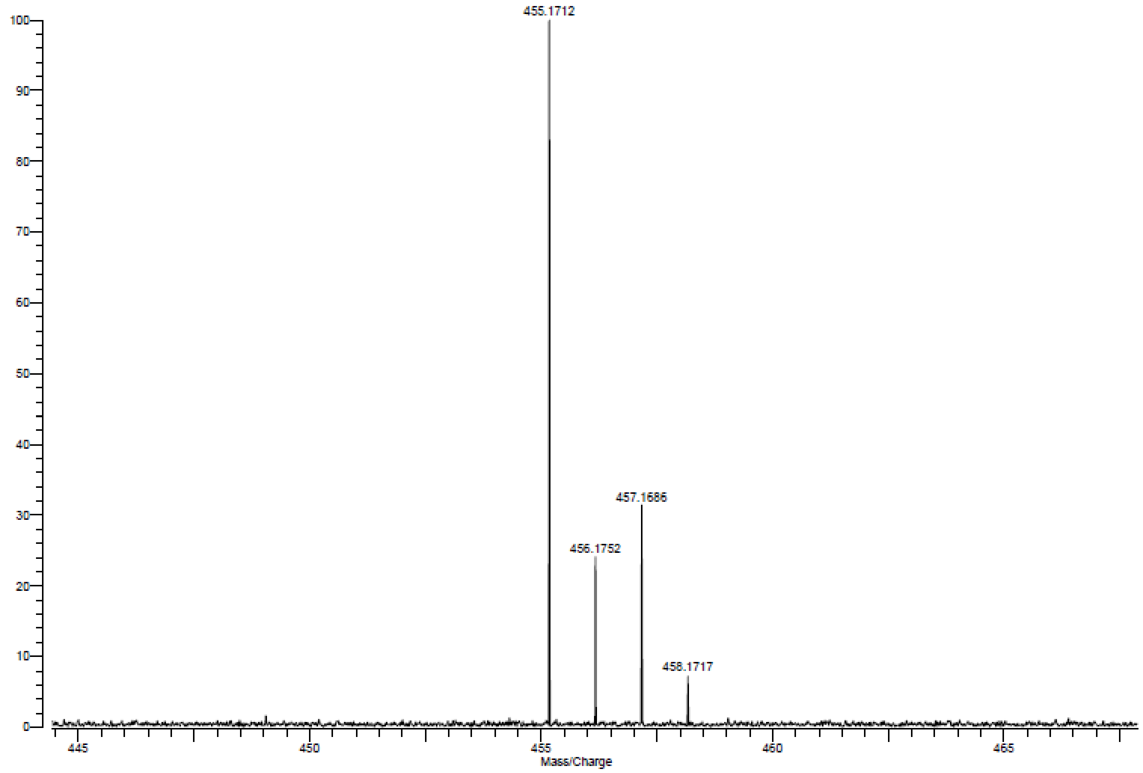

Figure 13 HRMS spectrum of **I-5**

I-6

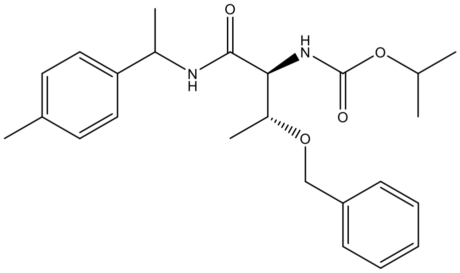

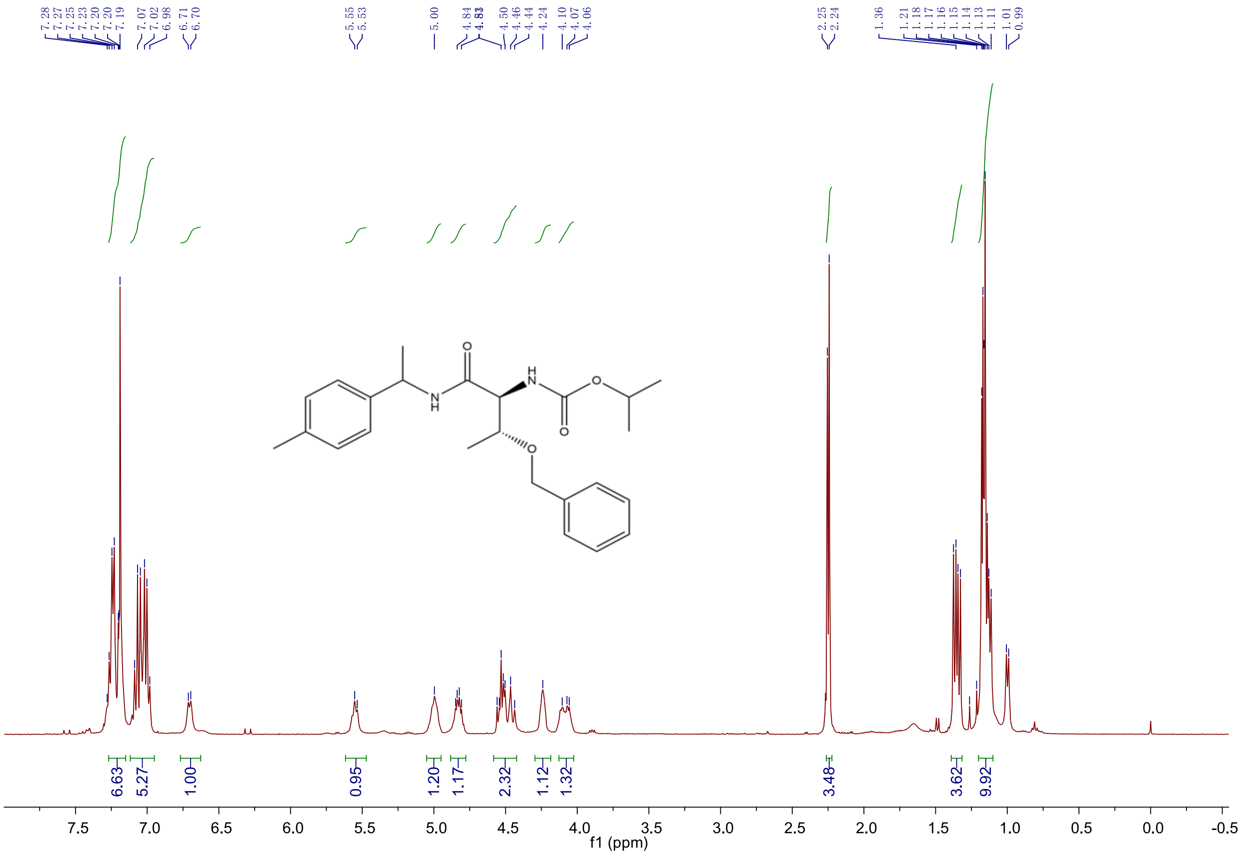

Figure 14 ^{1}HNMR spectrum of **I-6**

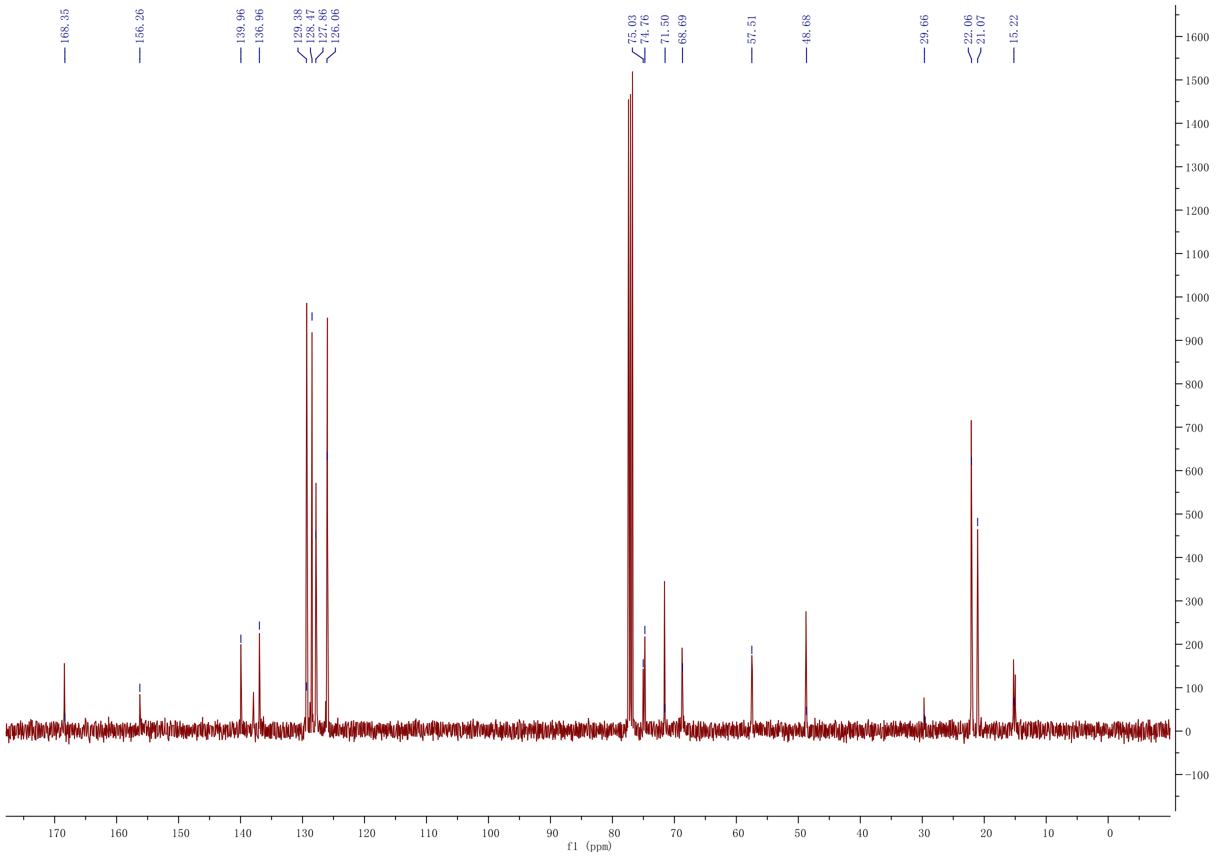

Figure 15 ^{13}C NMR spectrum of **I-6**

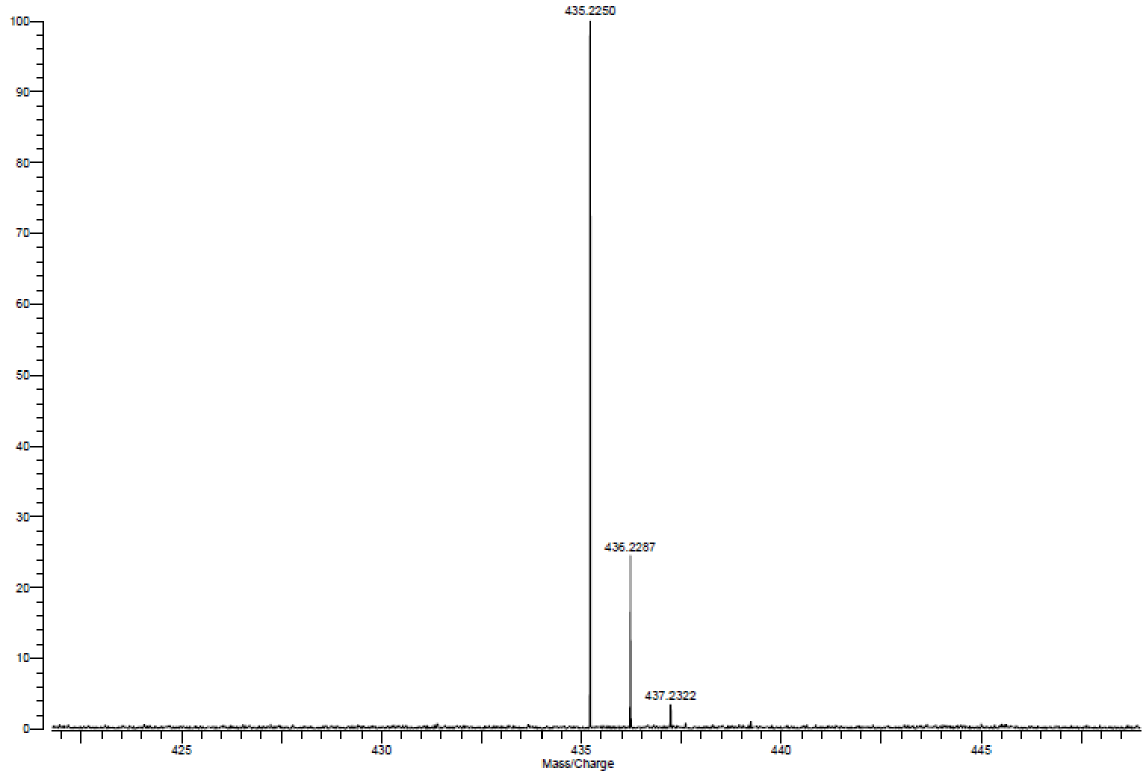

Figure 16 HRMS of compound I-6

I-7

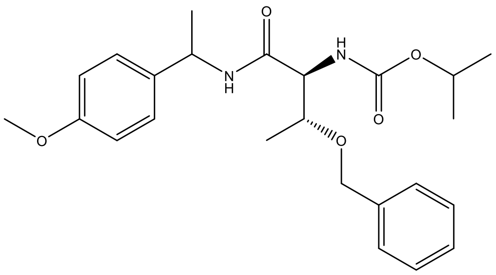

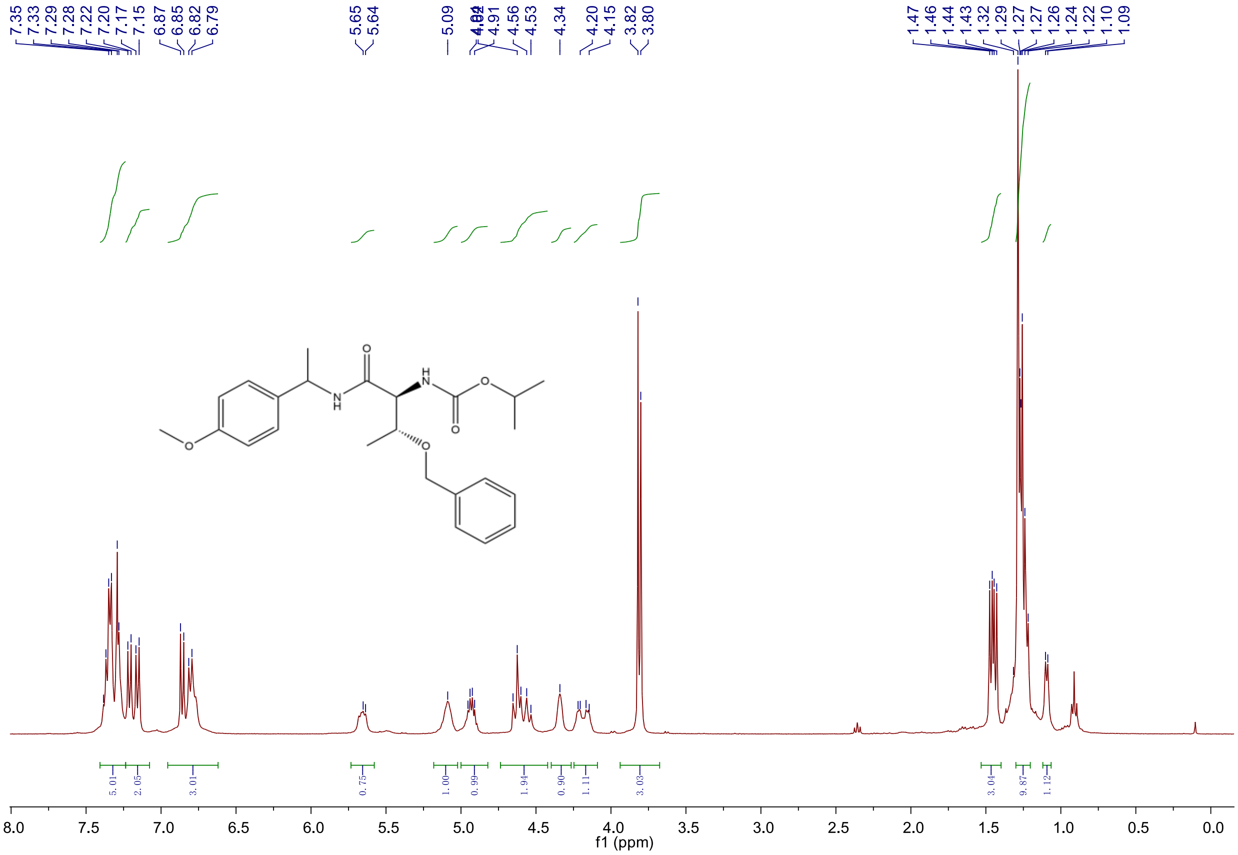

Figure 17 ^{1}HNMR spectrum of **I-7**

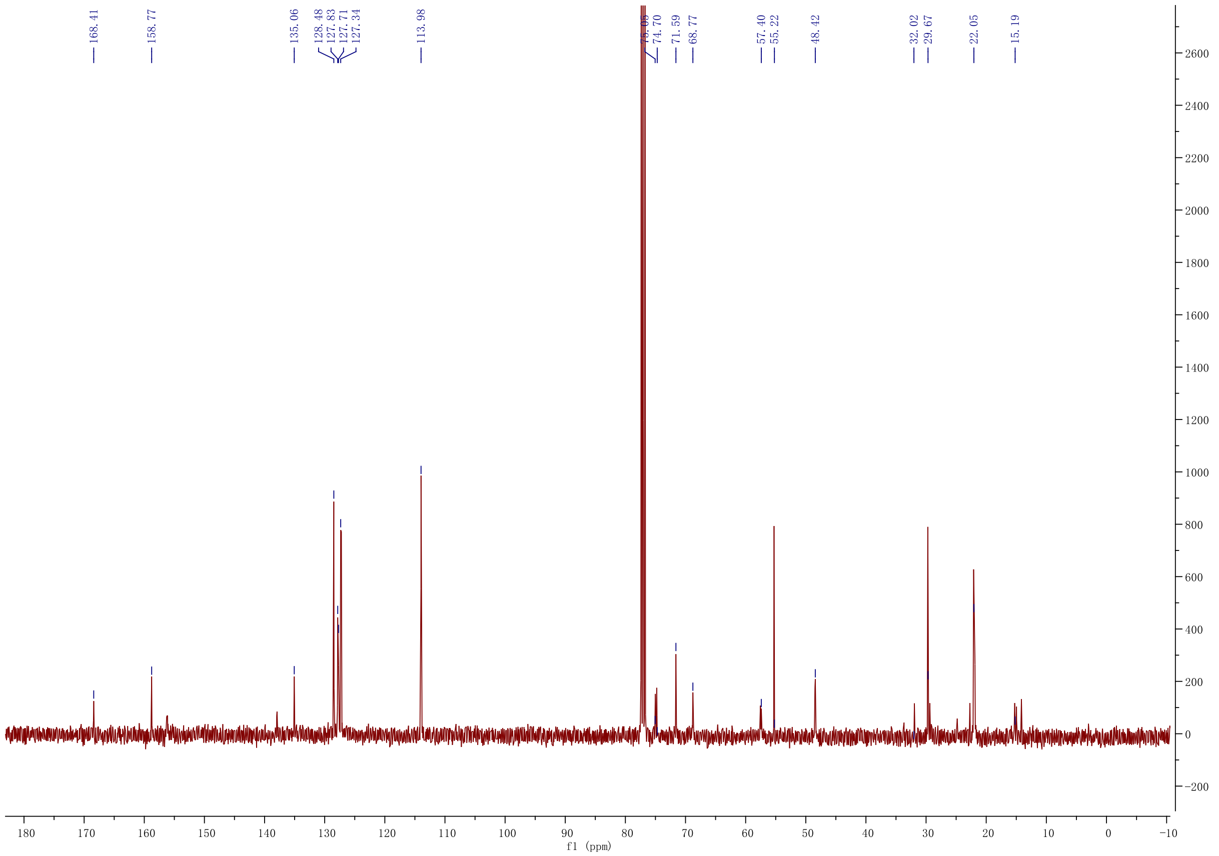

Figure 18 ^{13}C NMR spectrum of **I-7**

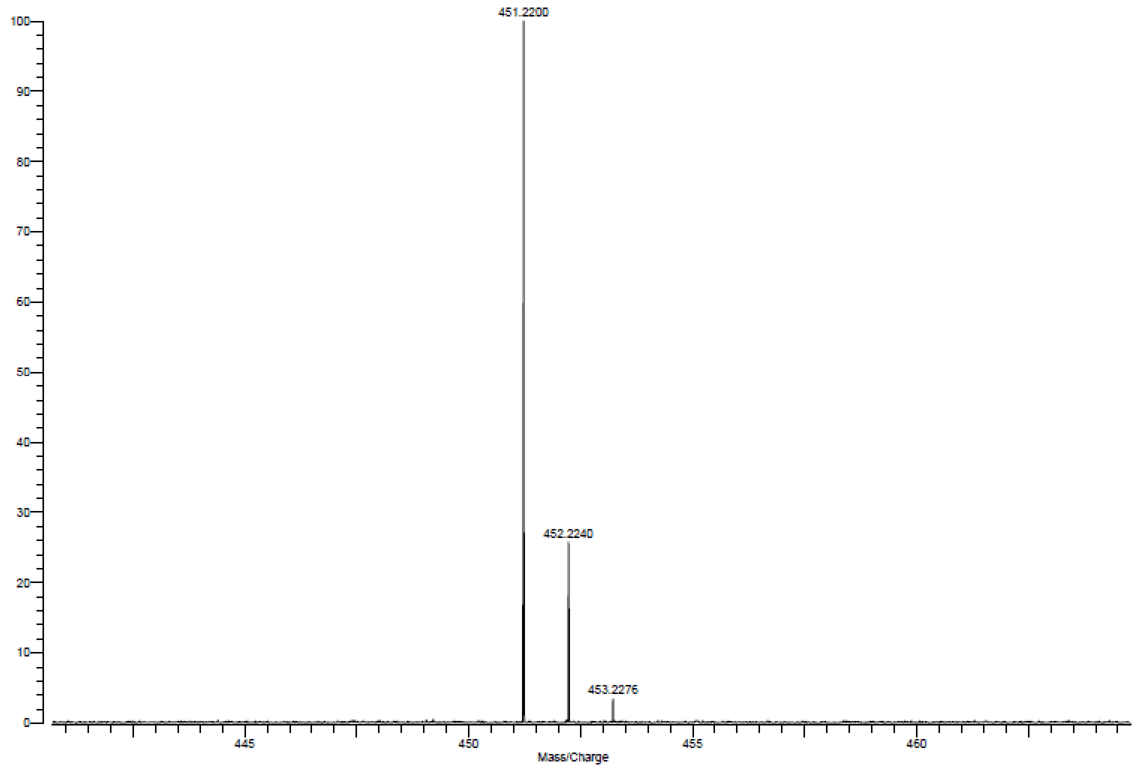

Figure 19 HRMS of **I-7**

I-8

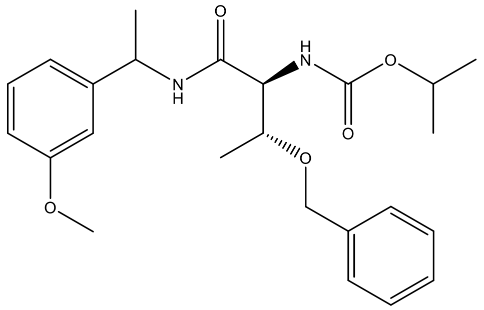

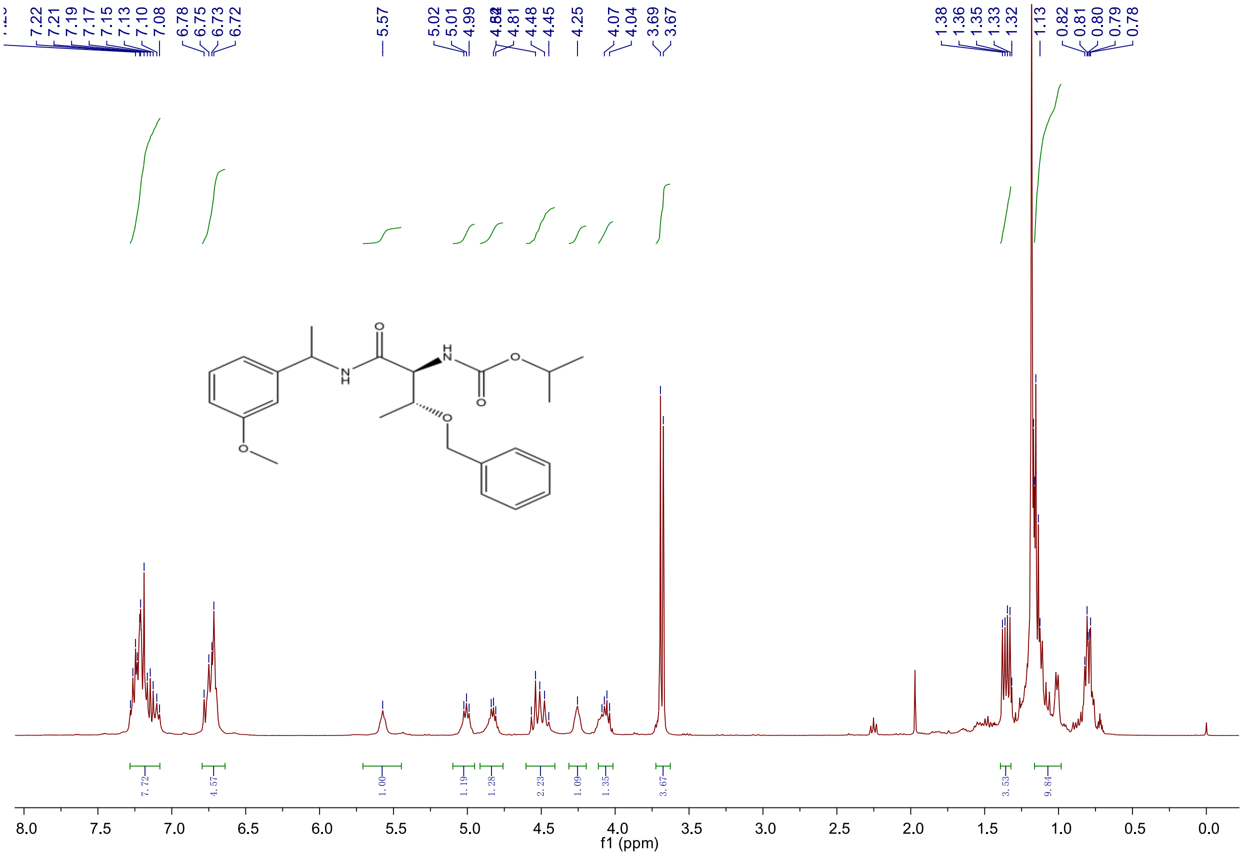

Figure 20 ^{1}HNMR spectrum of **I-8**

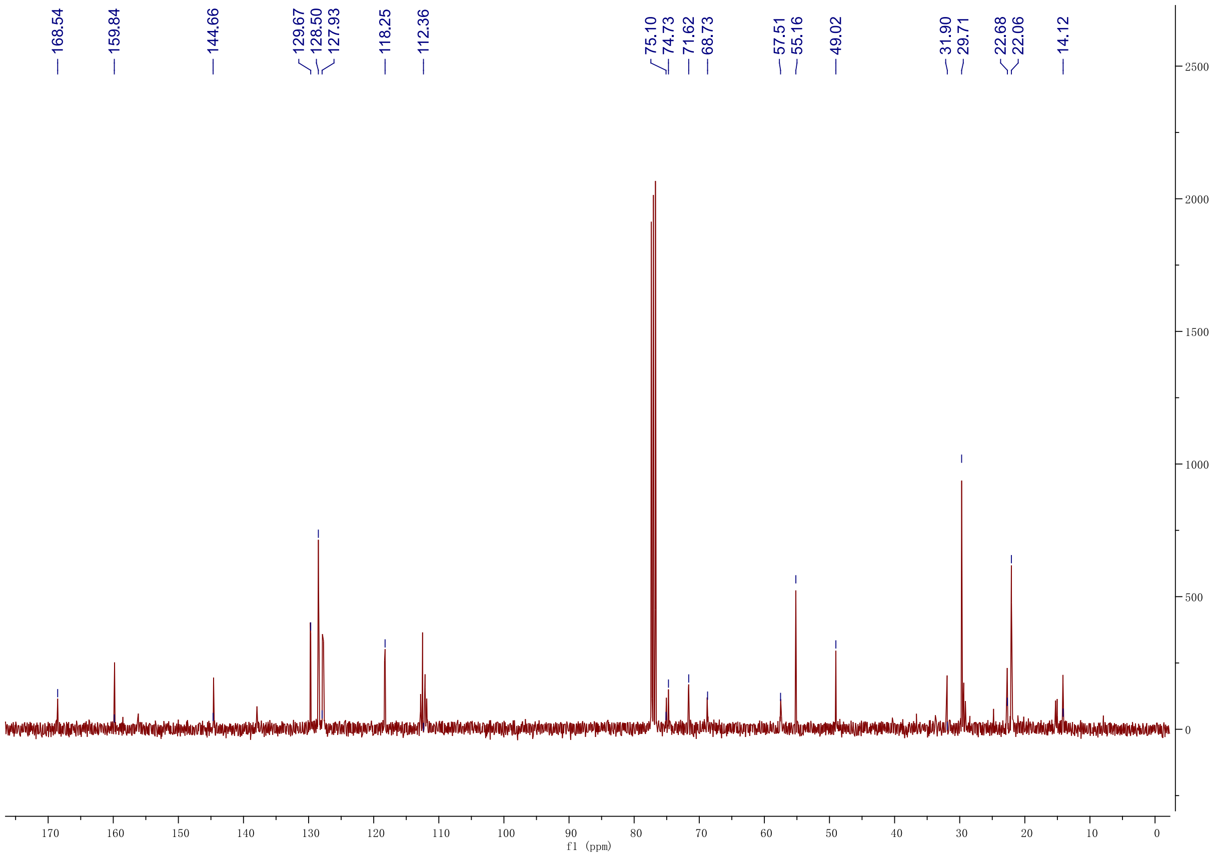

Figure 21 ^{13}C NMR spectrum of **I-8**

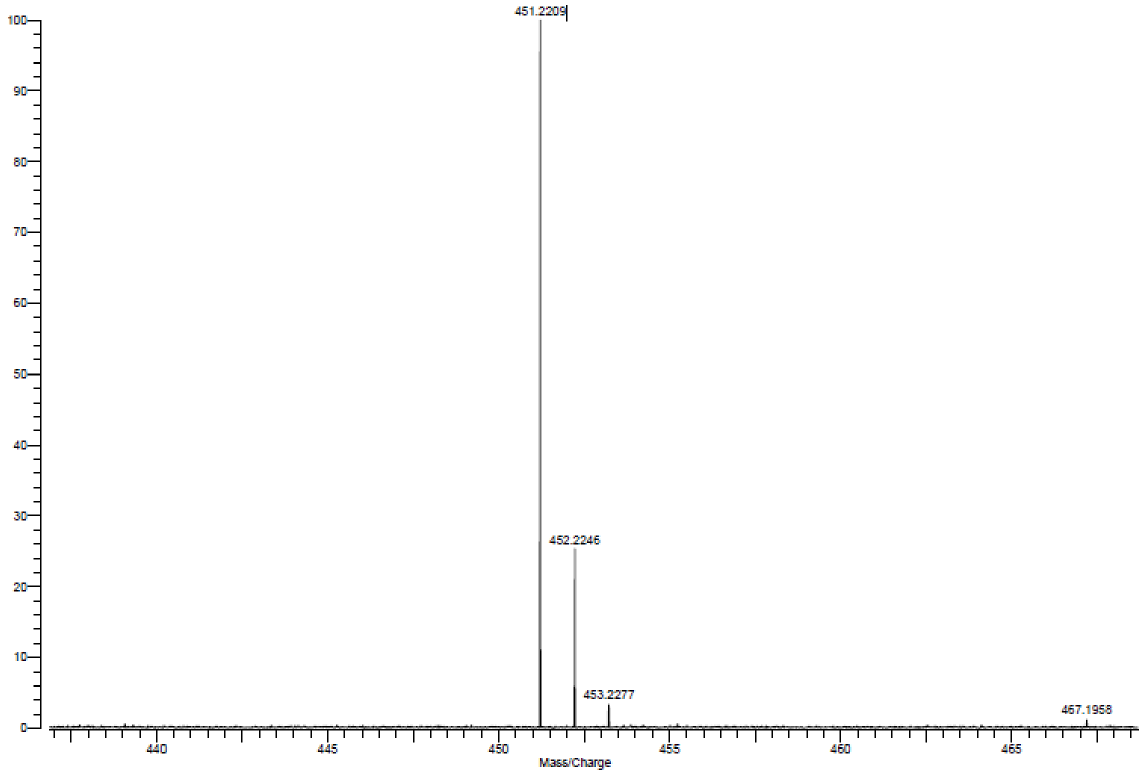

Figure 22 HRMS of I-8

I-9

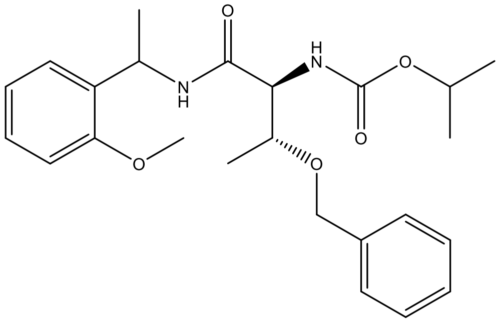

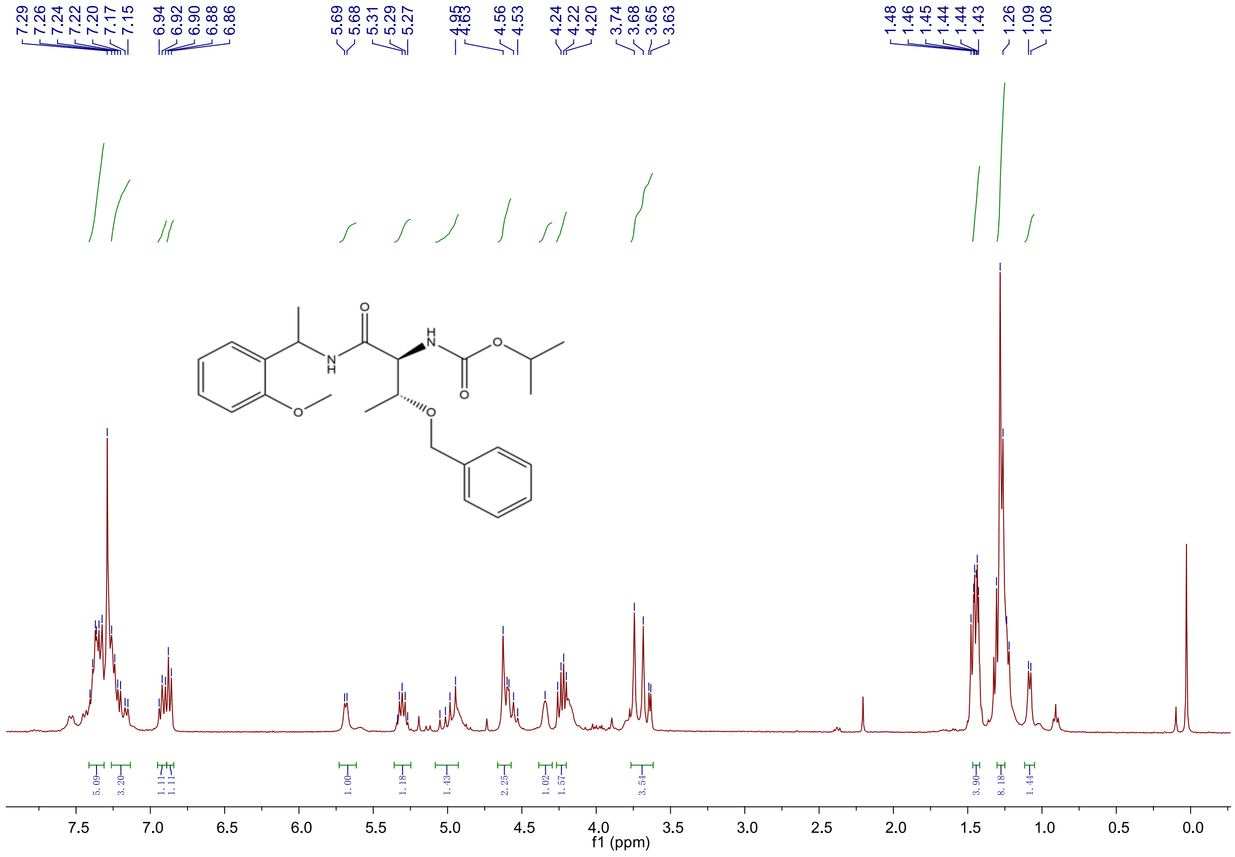

Figure 23 ^{1}HNMR spectrum of **I-9**

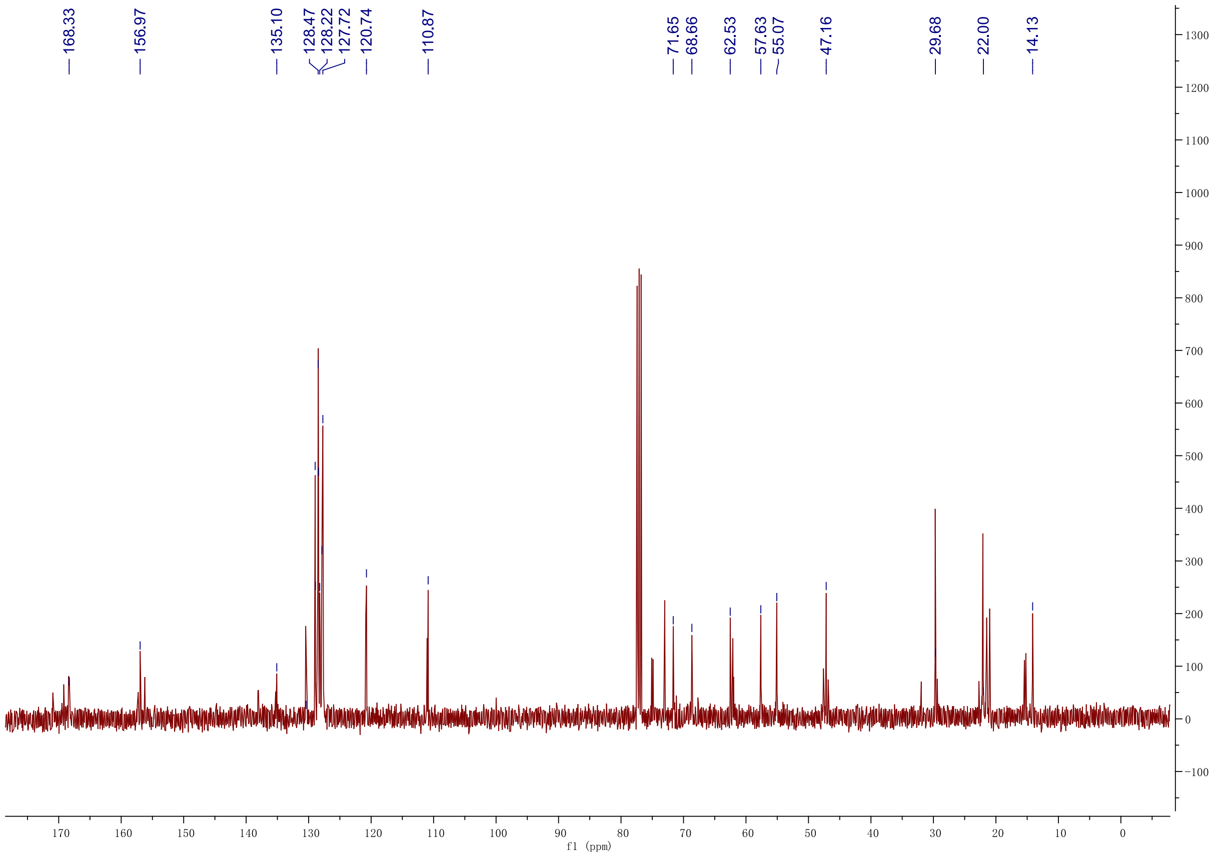

Figure 24 ^{13}C NMR spectrum of **I-9**

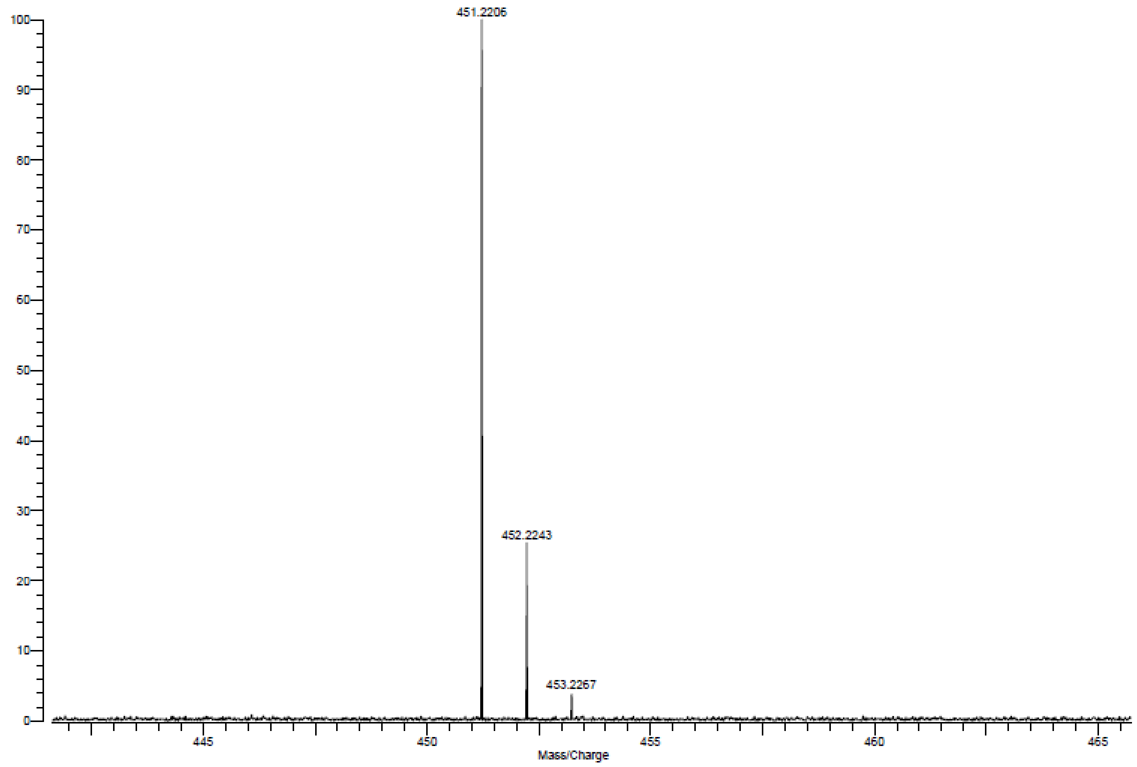

Figure 25 HRMS of I-9

I-10

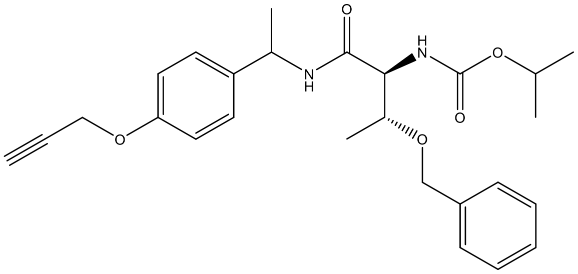

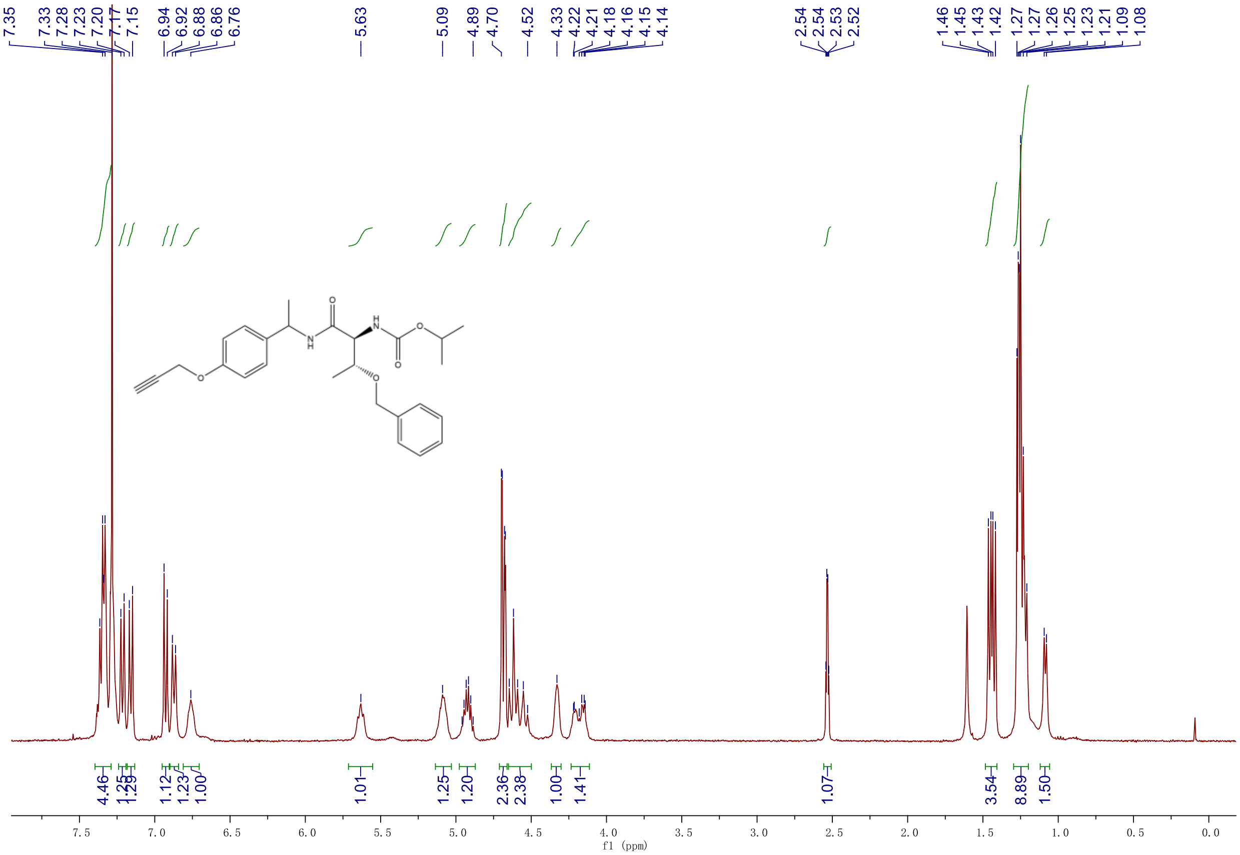

Figure 26 ^{1}HNMR spectrum of **I-10**

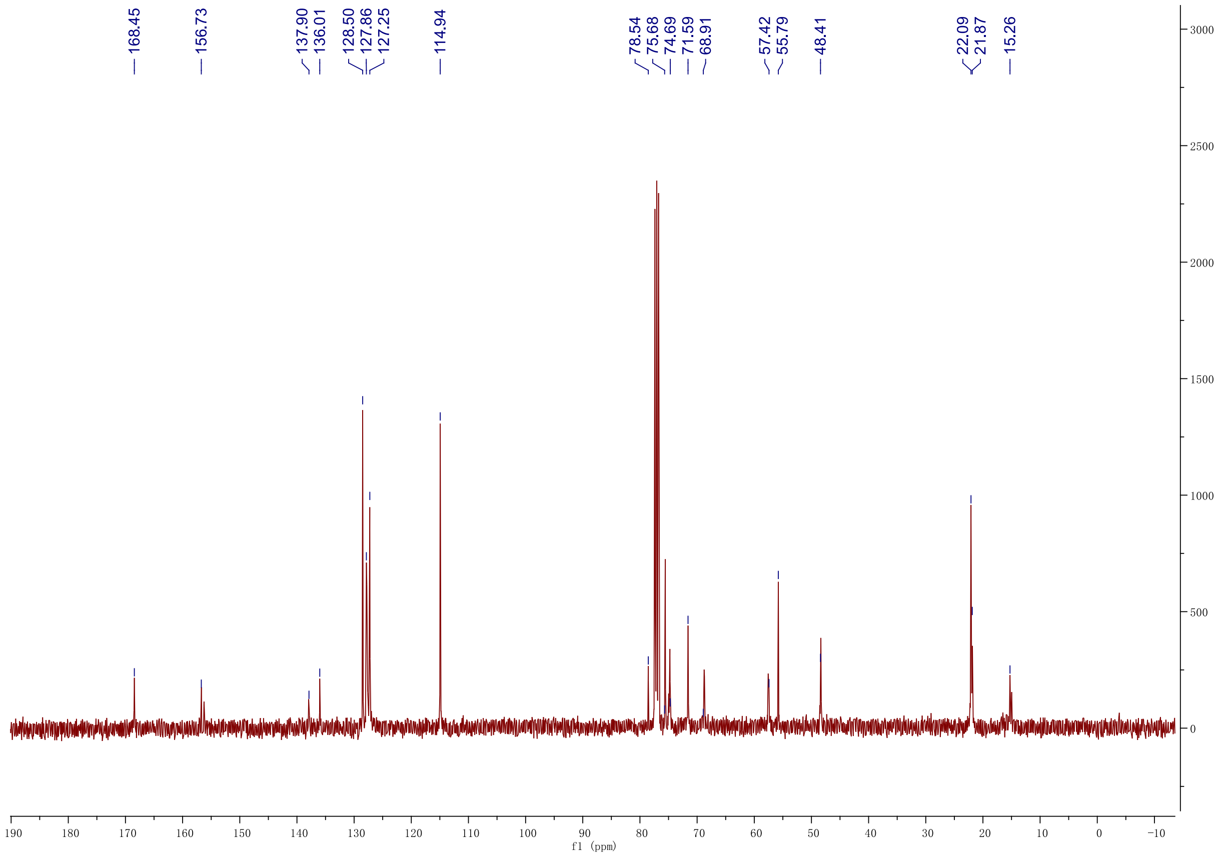

Figure 27 ^{13}C NMR spectrum of **I-10**

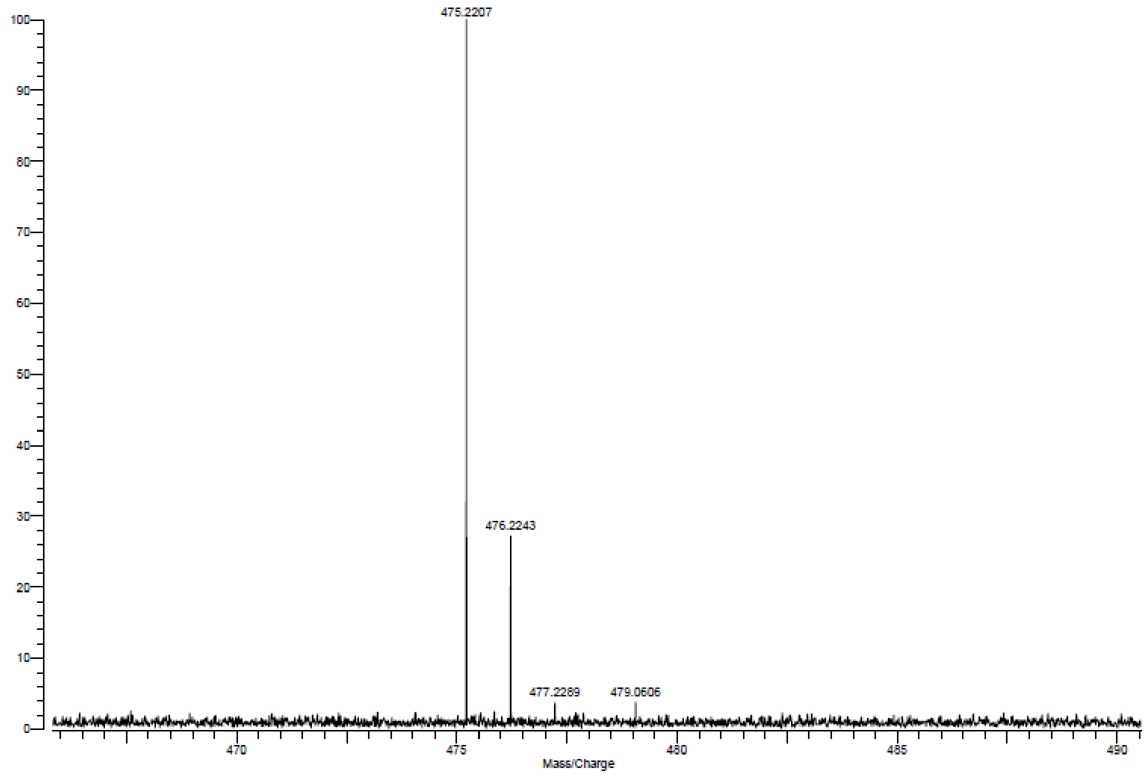

Figure 28 HRMS of I-10

I-13

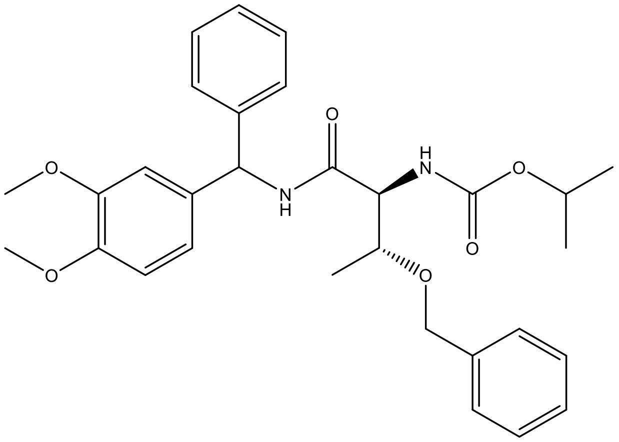

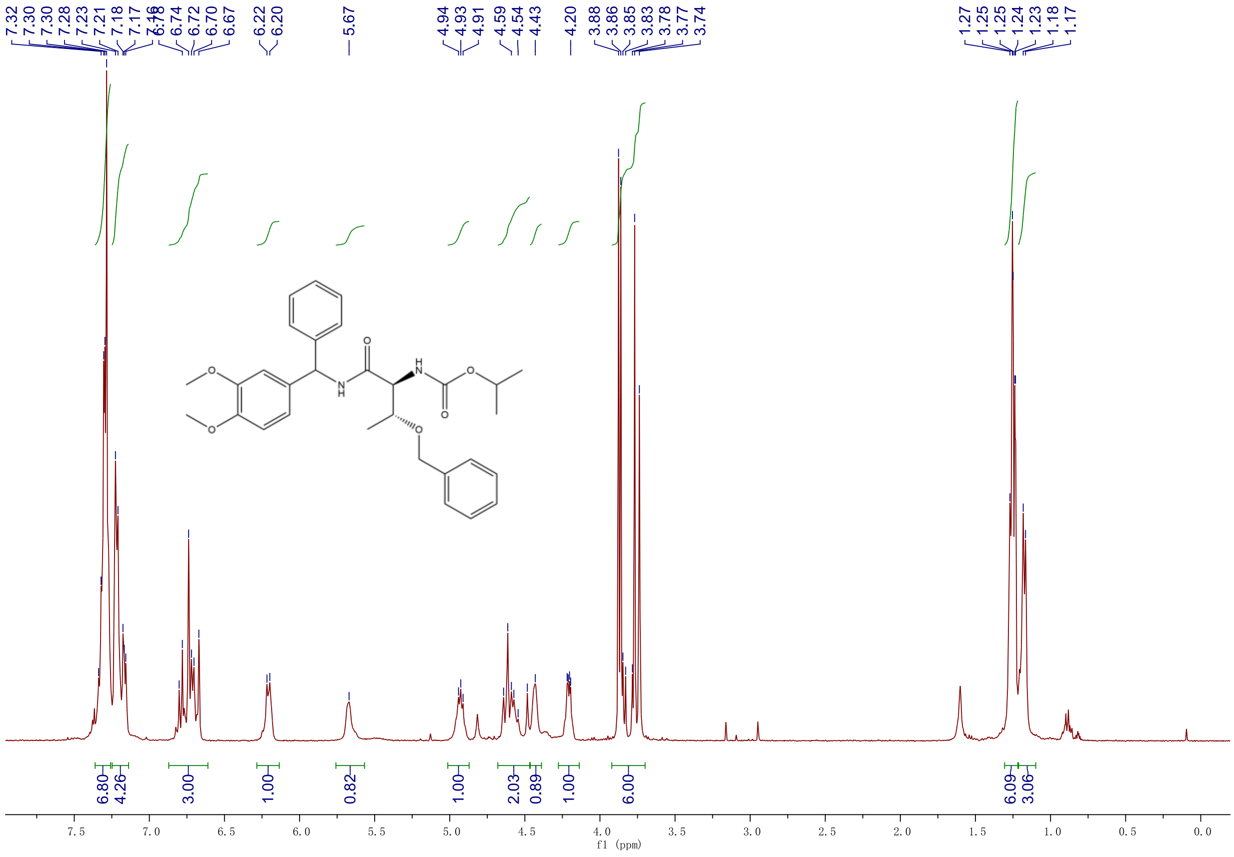

Figure 29 ¹HNMR spectrum of **I-13**

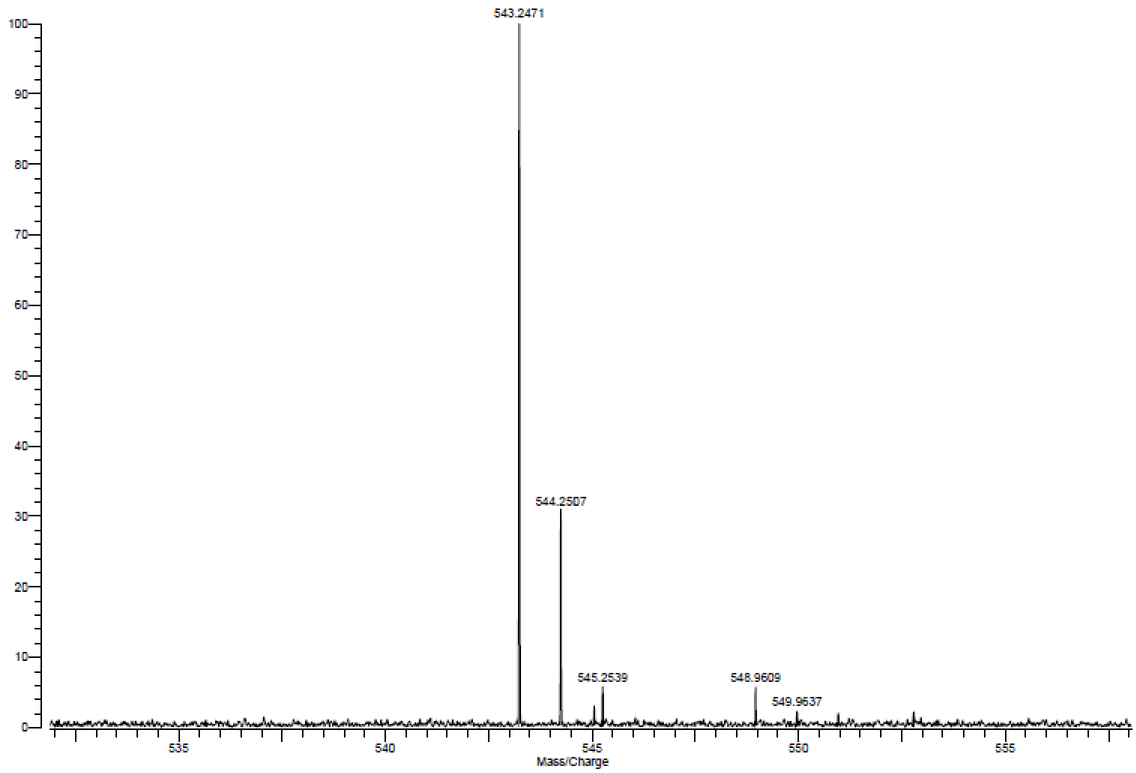

Figure 30 HRMS of I-13

I-14

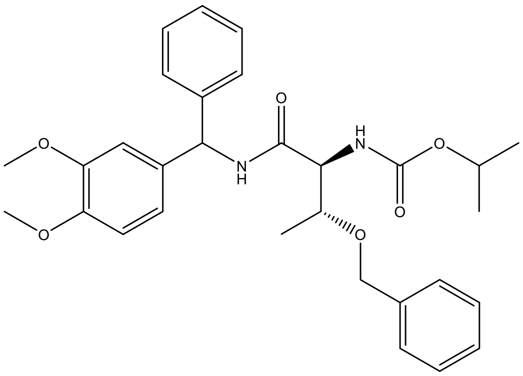

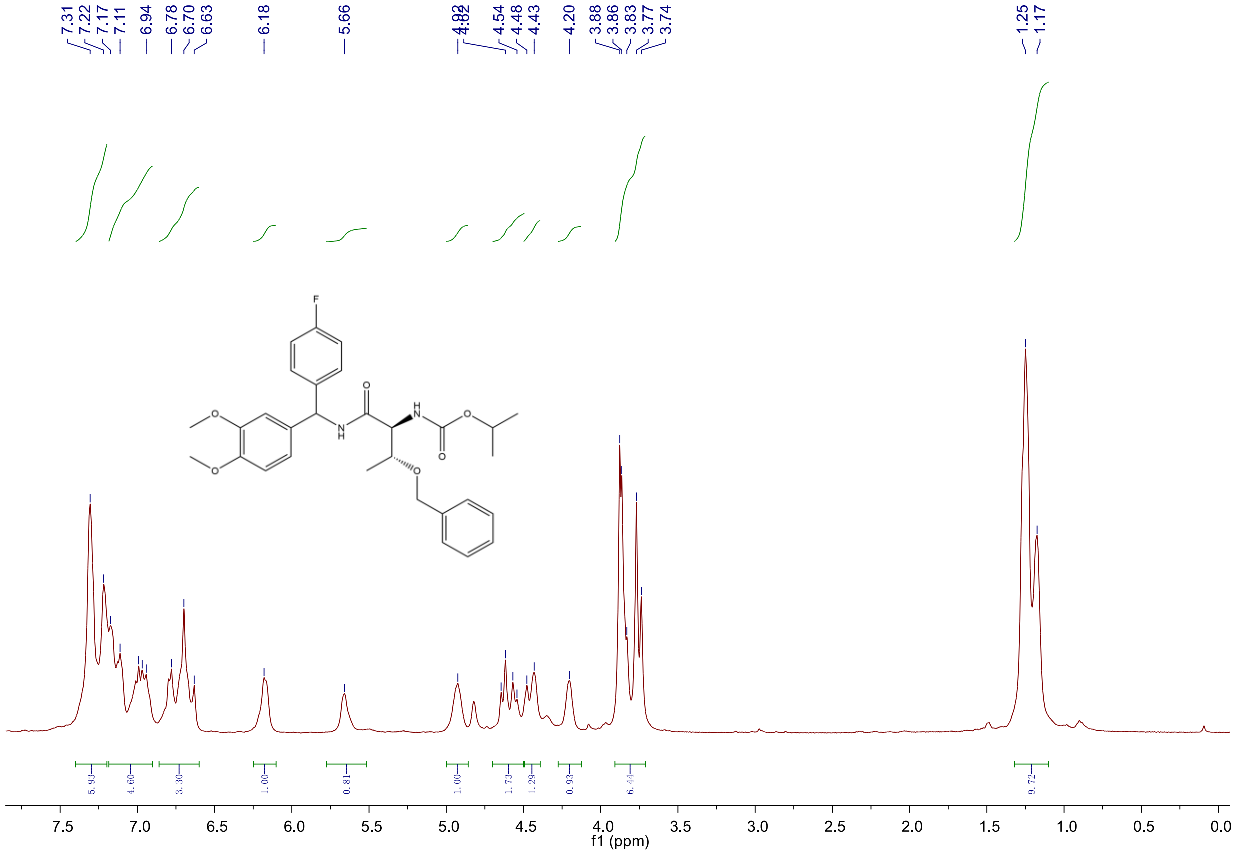

Figure 31 ^{1}HNMR spectrum of **I-14**

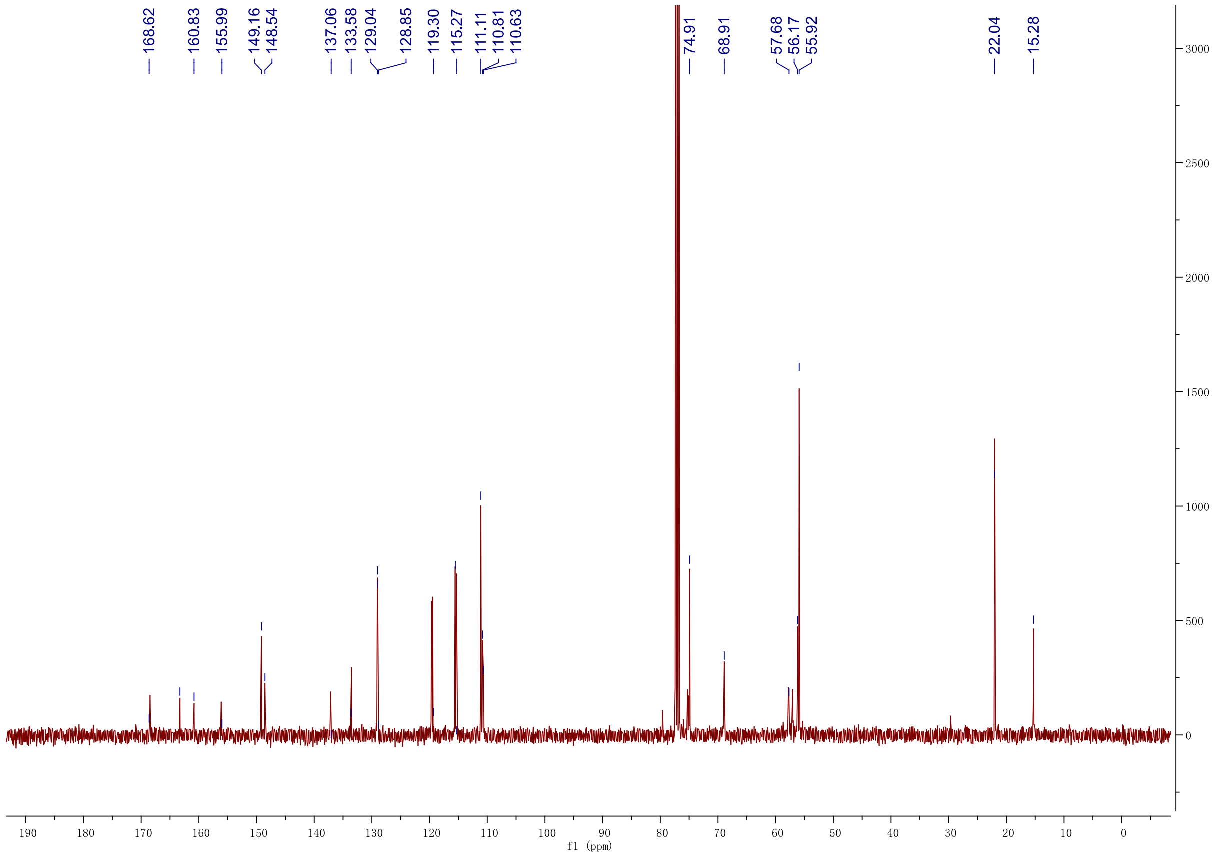

Figure 32 ^{13}C NMR spectrum of **I-14**

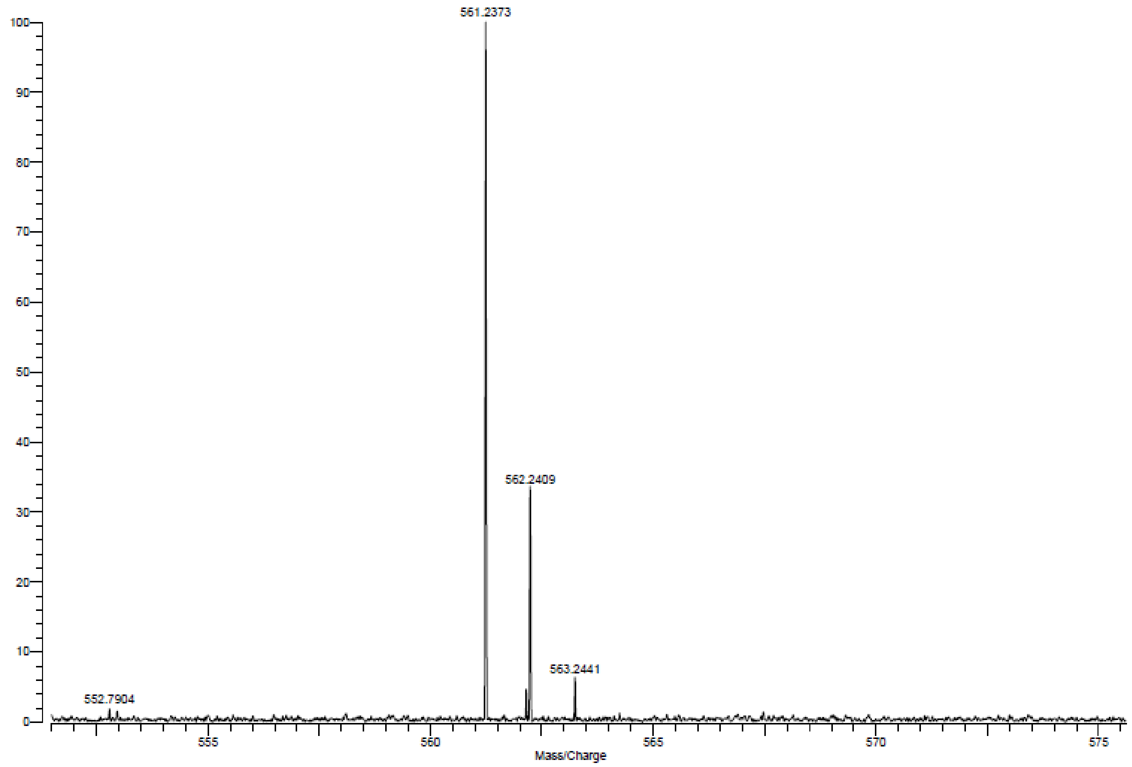

Figure 33 HRMS of **I-14**

I-15

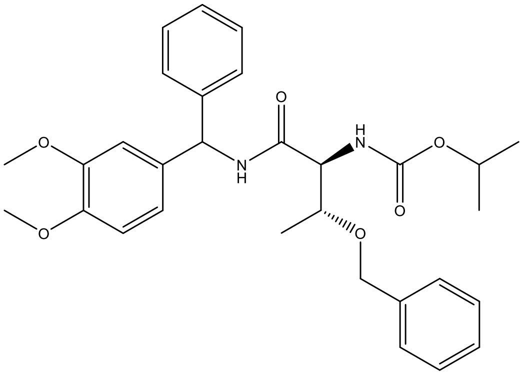

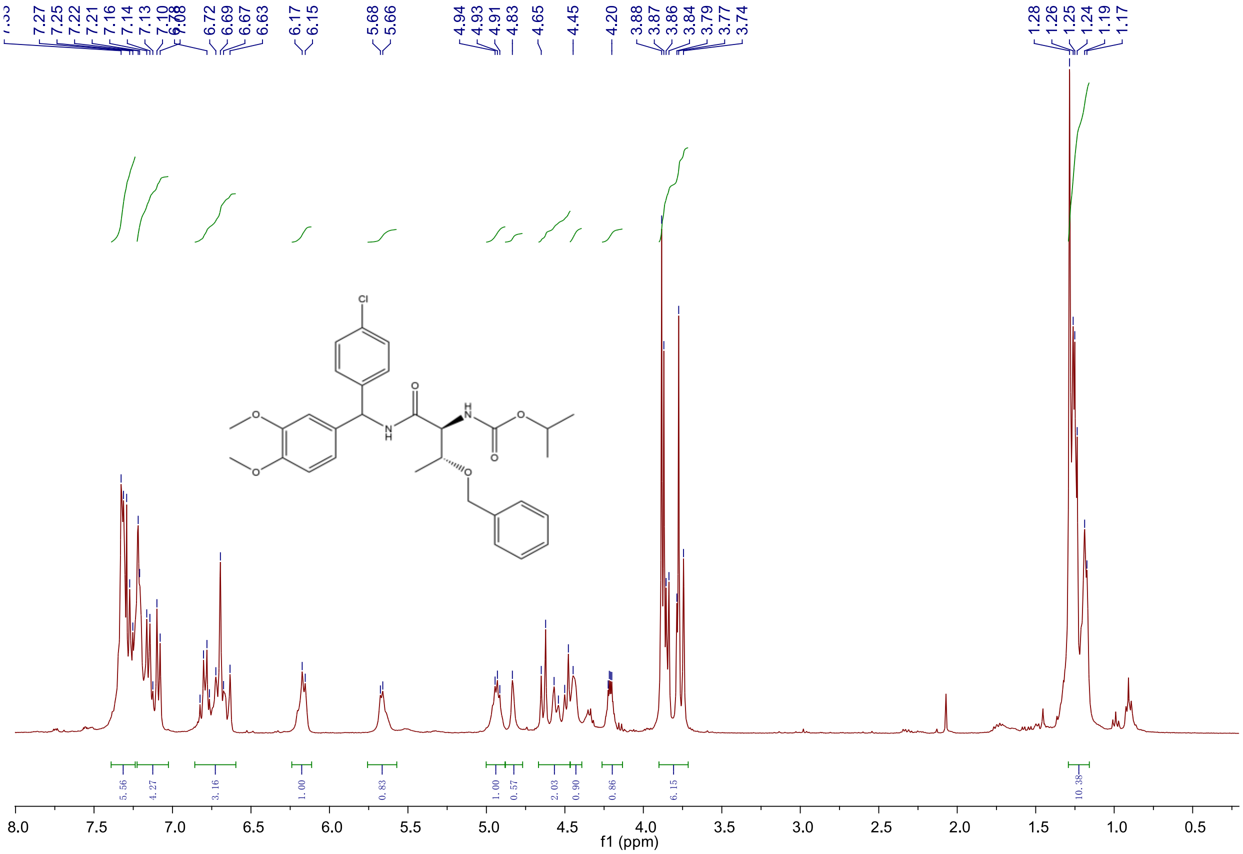

Figure 34 ^{1}HNMR spectrum of **I-15**

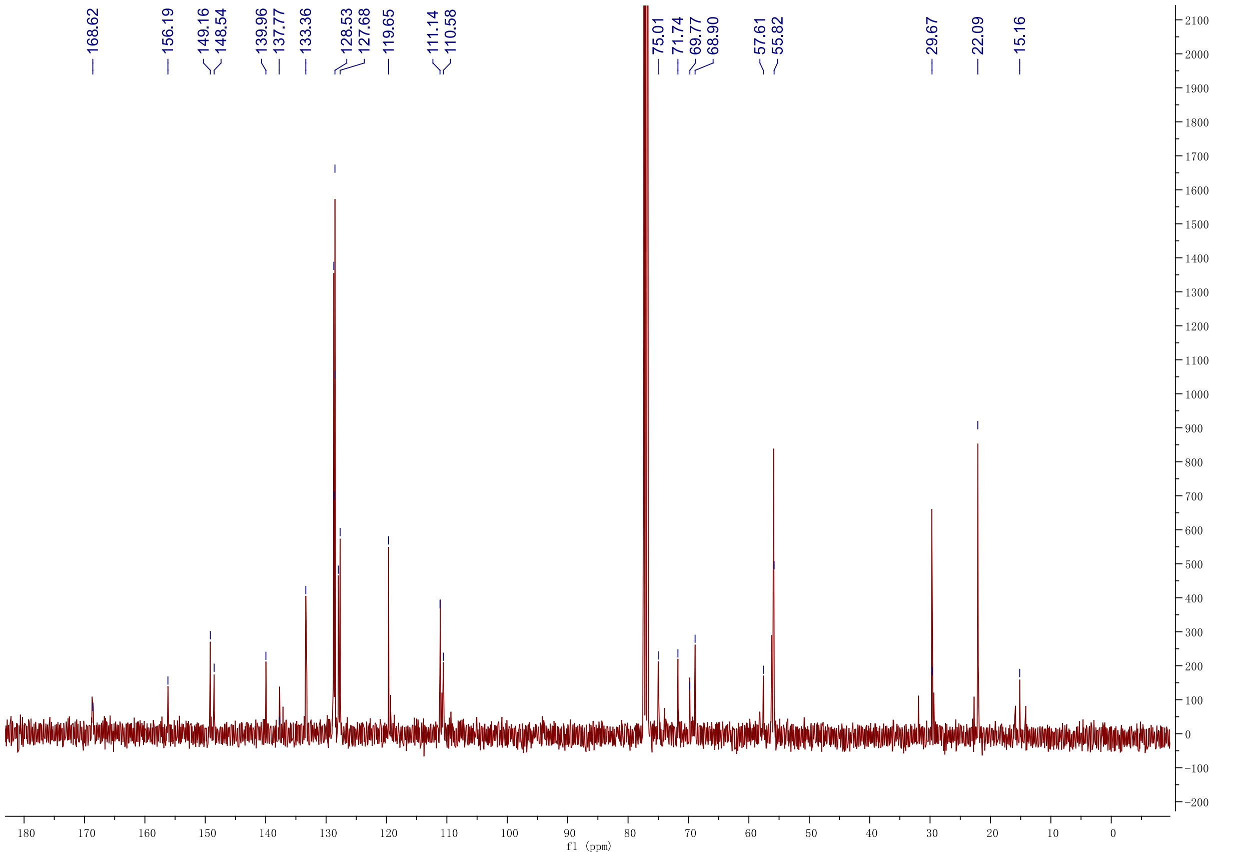

Figure 35 ^{13}C NMR spectrum of **I-15**

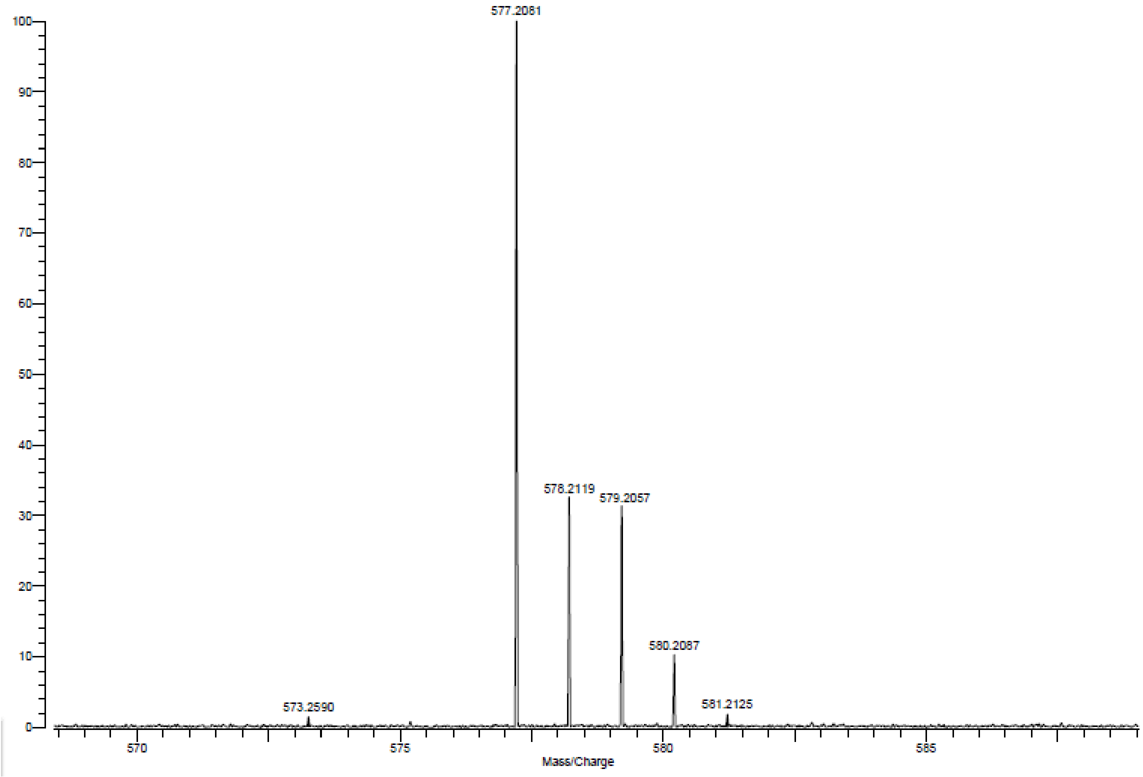

Figure 36 HRMS of **I-15**

I-16

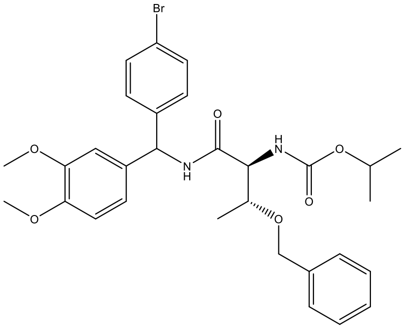

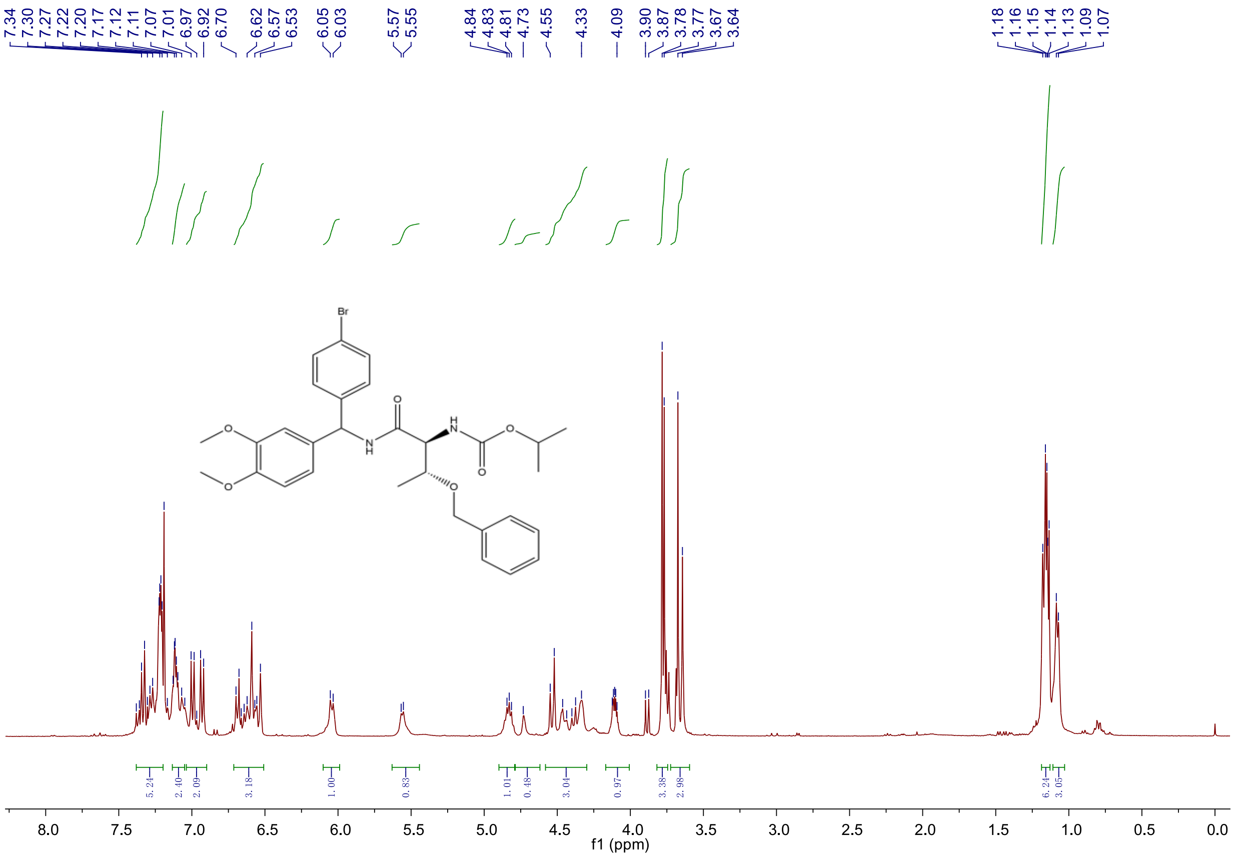

Figure 37 ^{1}HNMR spectrum of **I-16**

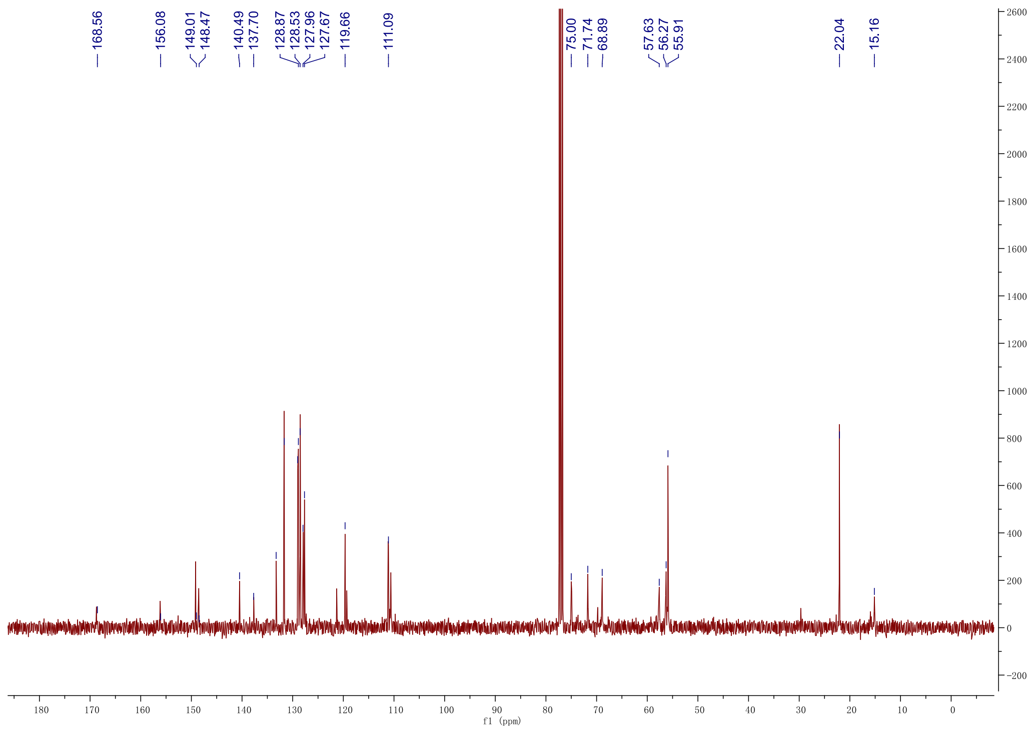

Figure 38 ^{13}C NMR spectrum of **I-16**

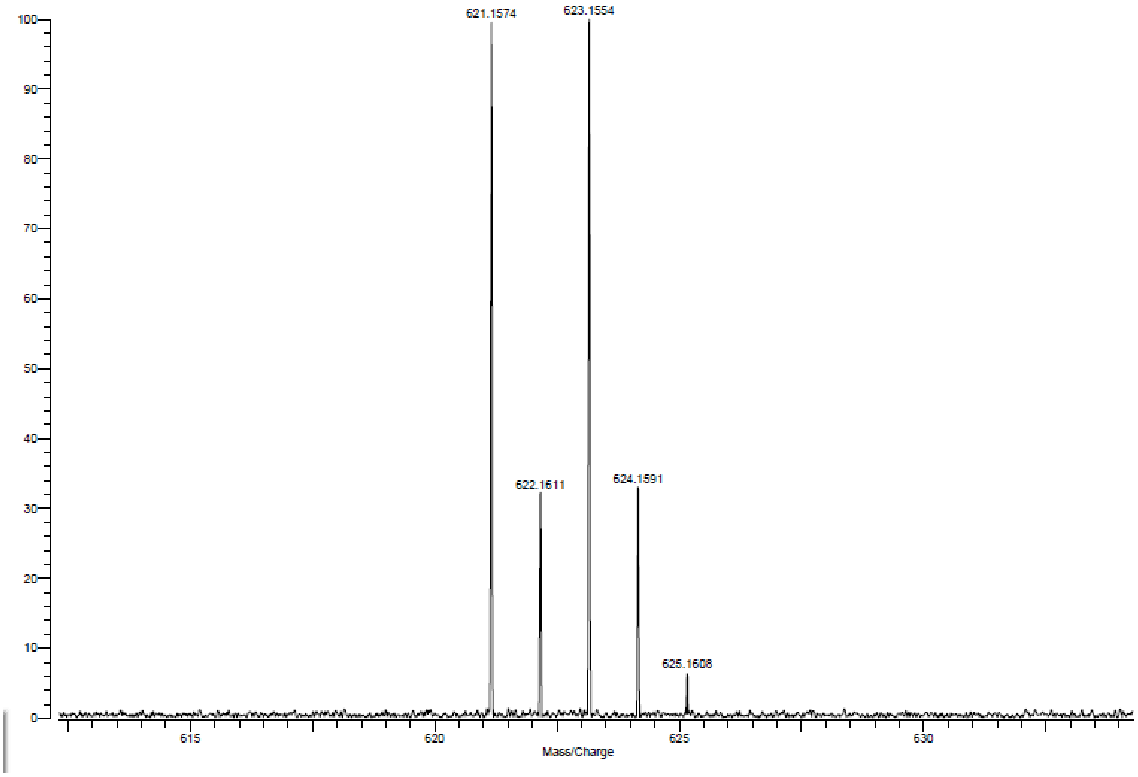

Figure 39 HRMS of **I-16**

I-18

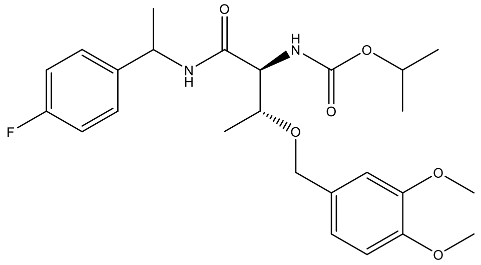

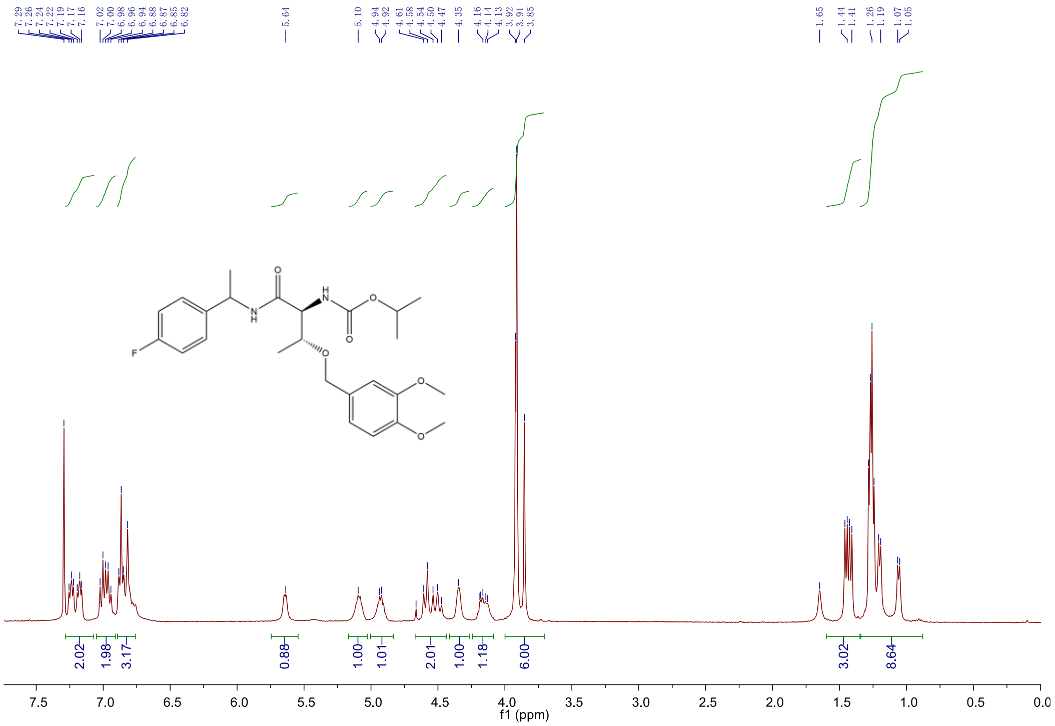

Figure 40 ^{1}HNMR spectrum of **I-18**

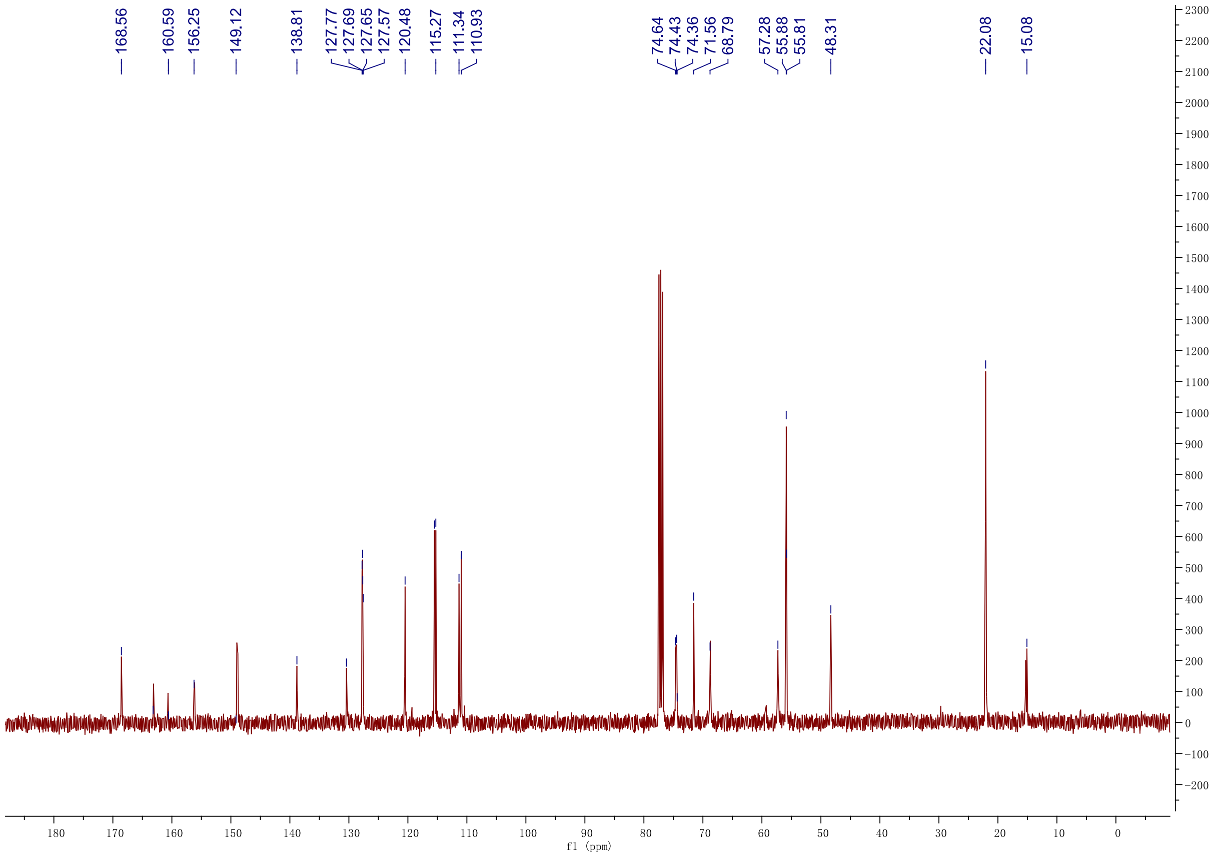

Figure 41 ^{13}C NMR spectrum of **I-18**

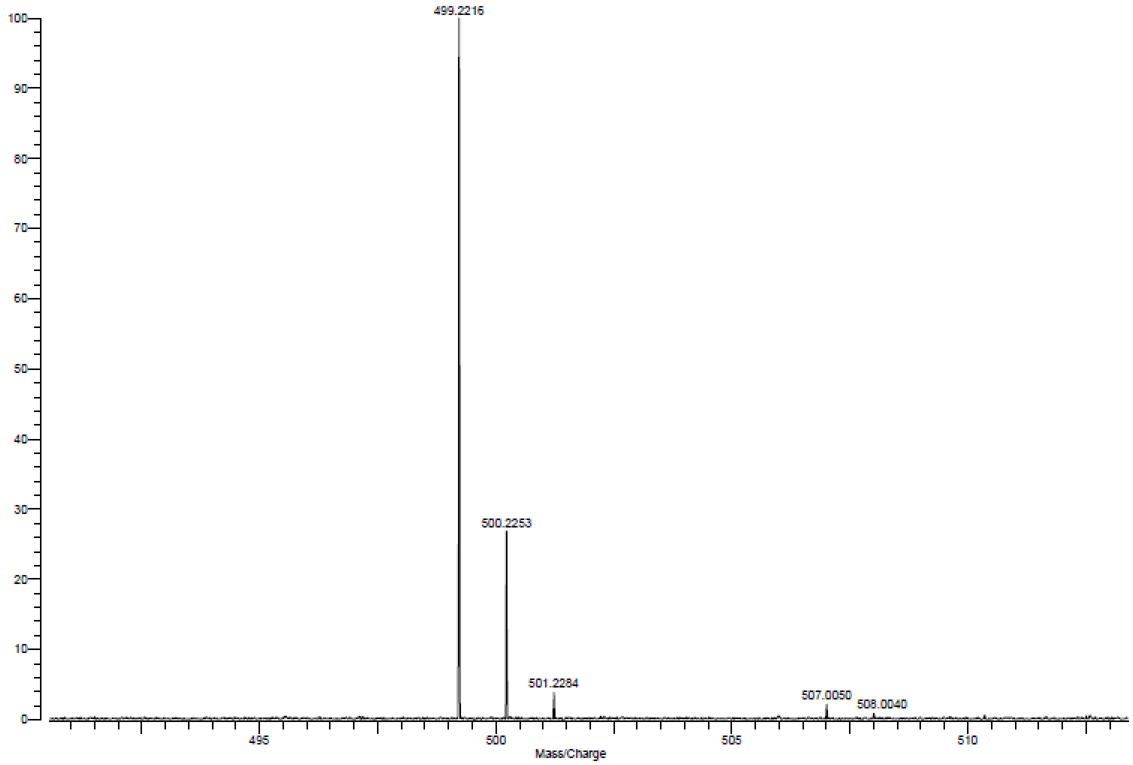

Figure 42 HRMS of **I-18**

I-19

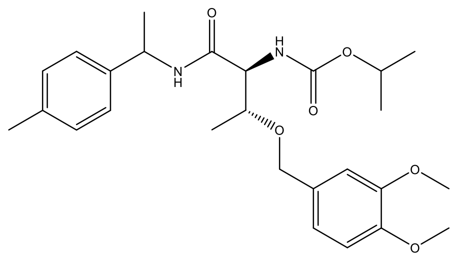

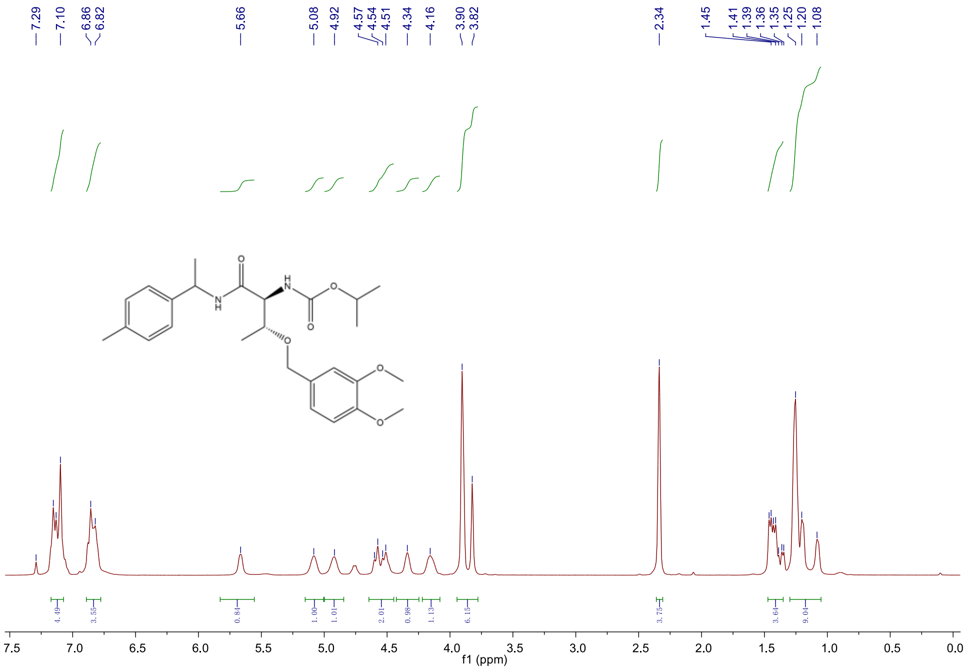

Figure 43 ^{1}HNMR spectrum of **I-19**

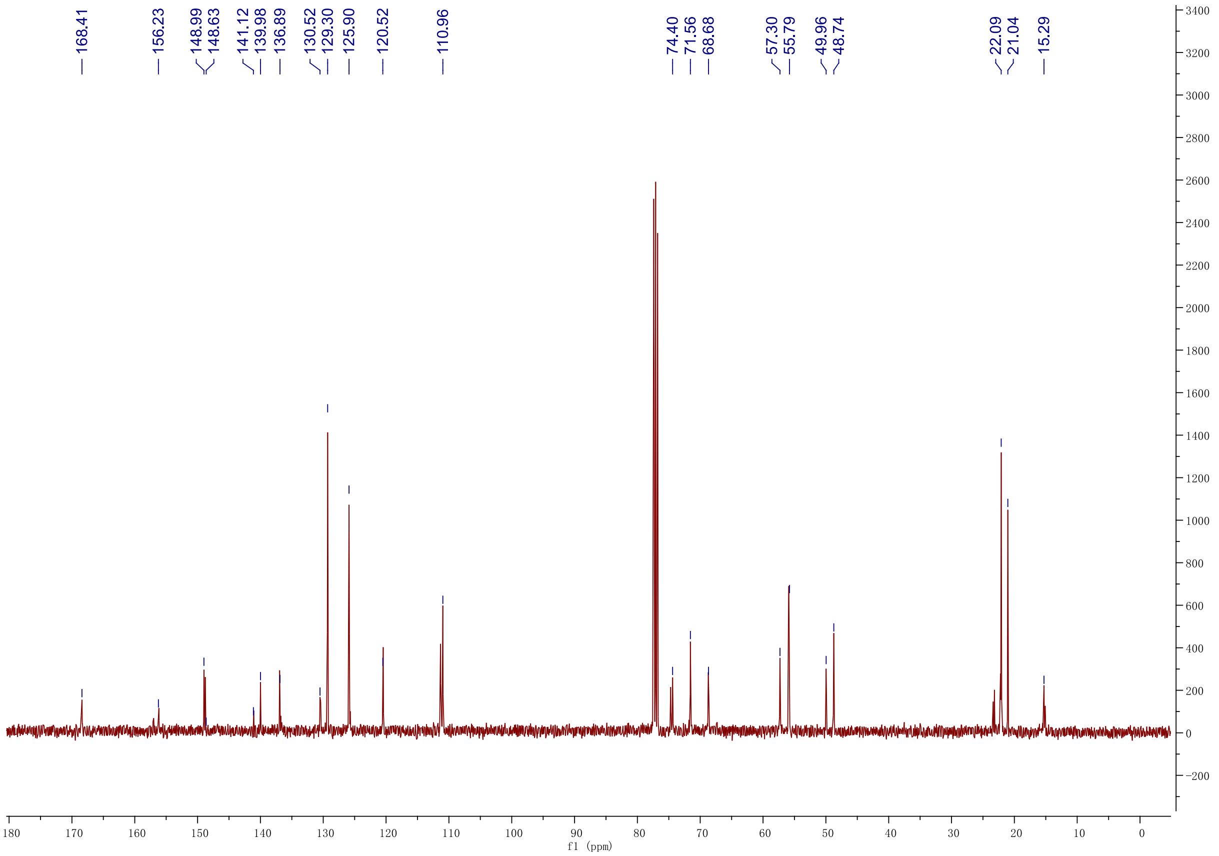

Figure 44 ^{13}C NMR spectrum of **I-19**

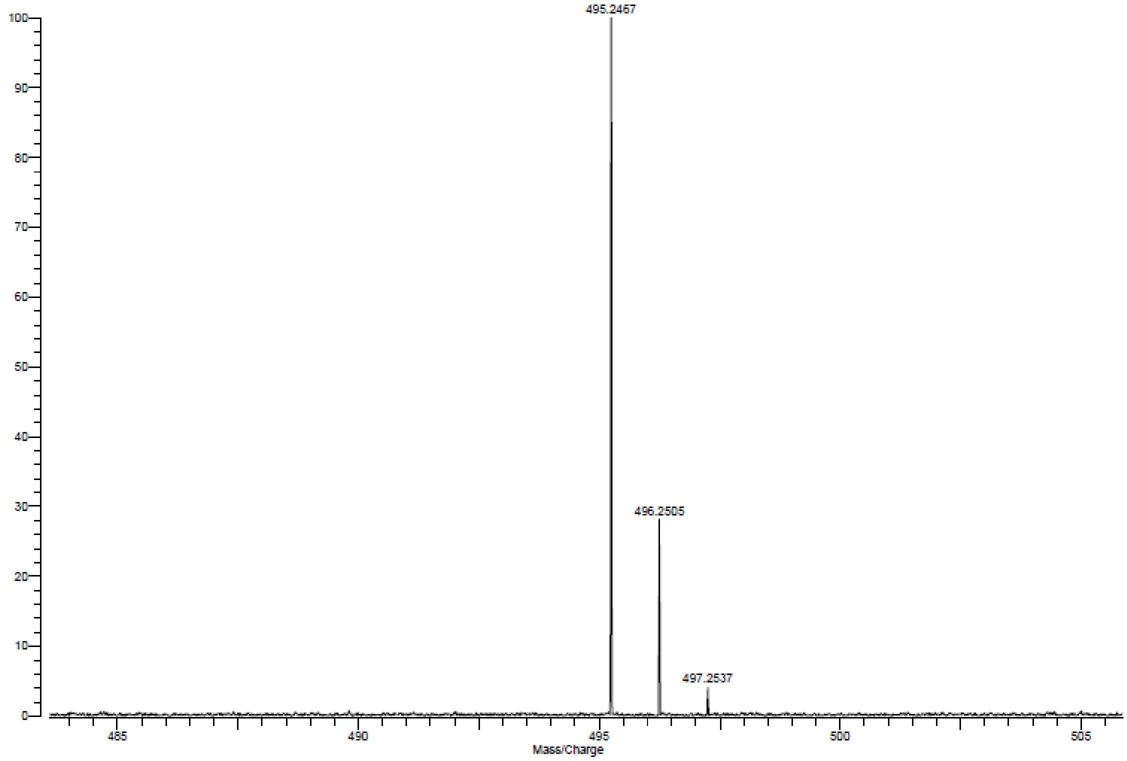

Figure 45 HRMS of **I-19**

I-20

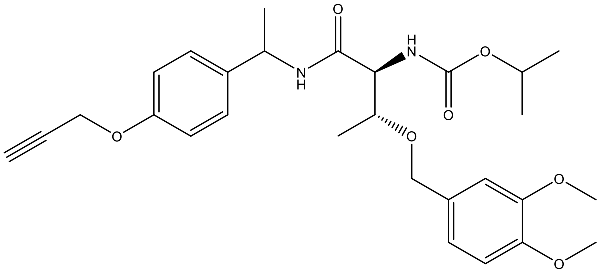

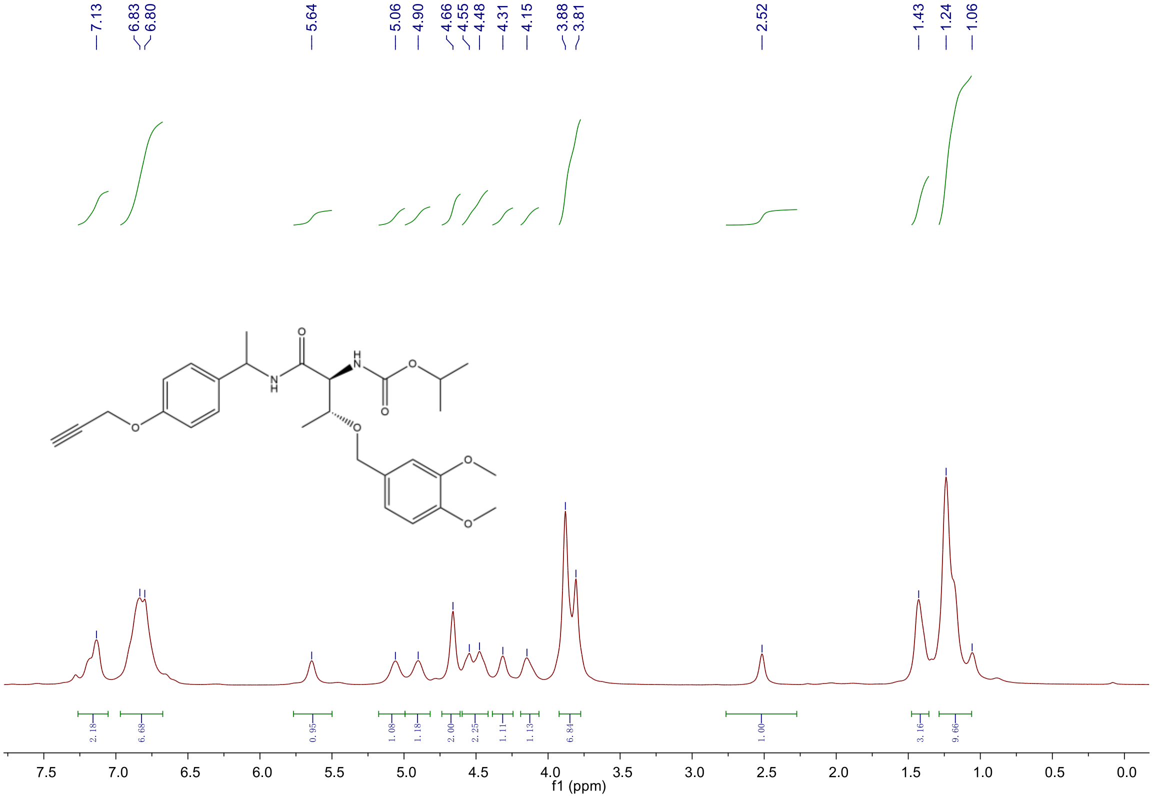

Figure 46 ^{1}HNMR spectrum of **I-20**

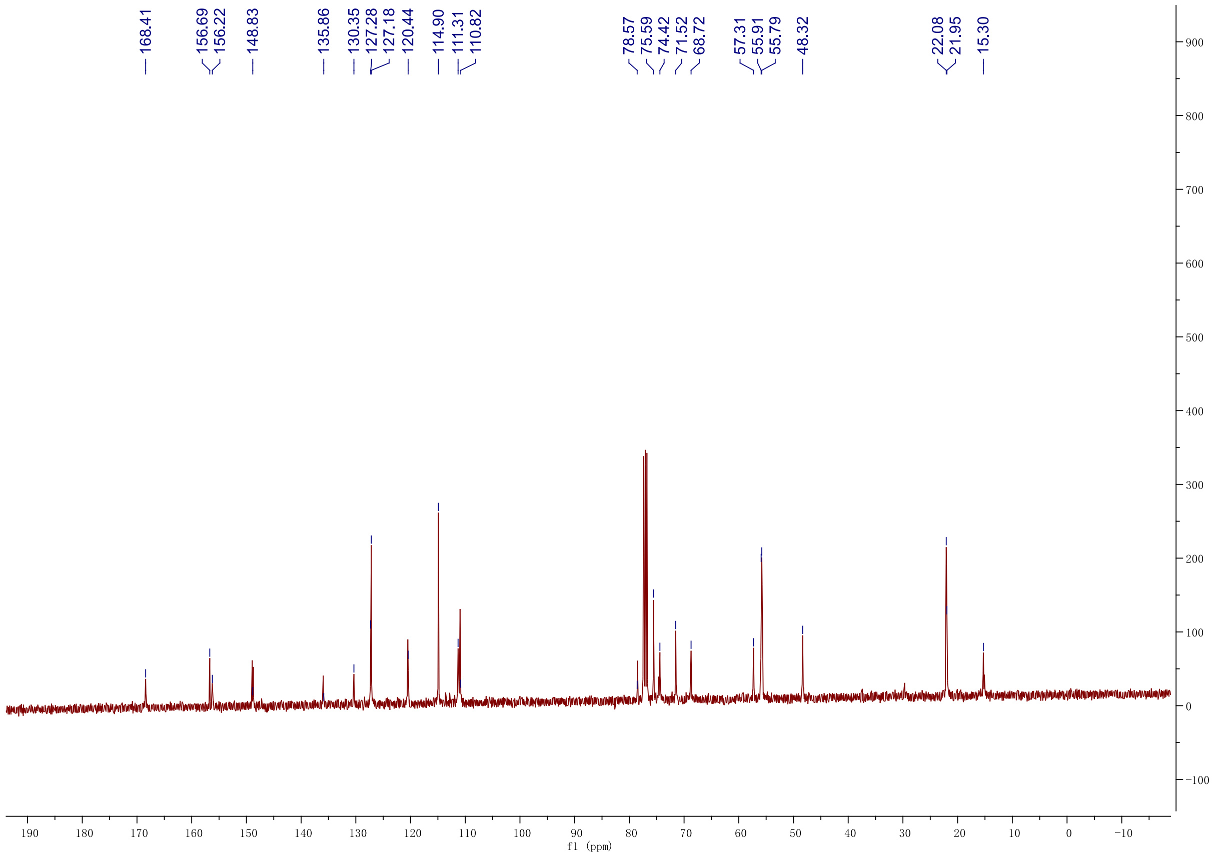

Figure 47 ^{13}C NMR spectrum of **I-20**

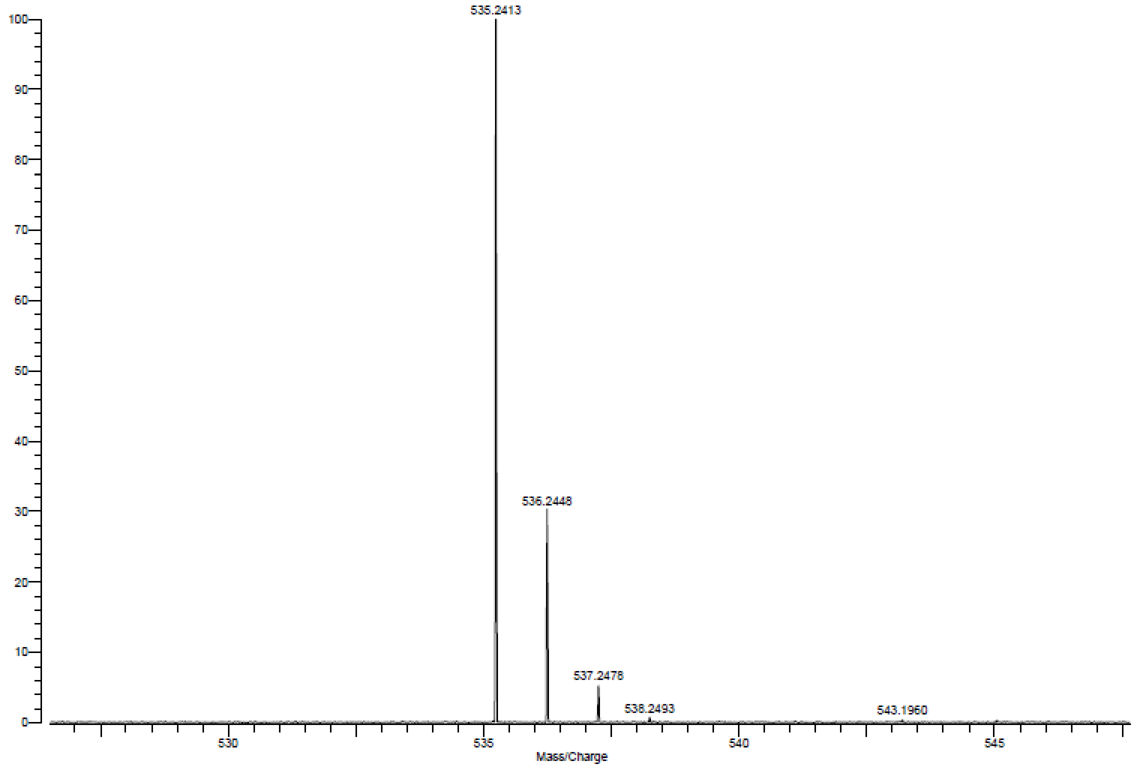

Figure 48 HRMS of **I-20**

I-21

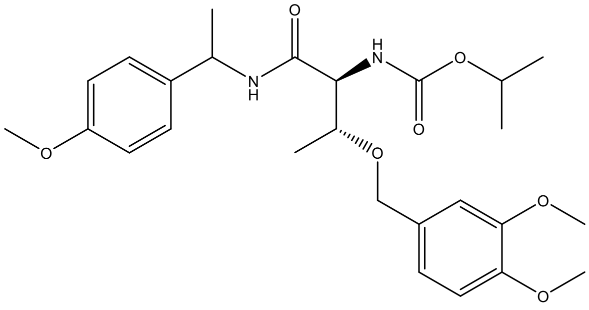

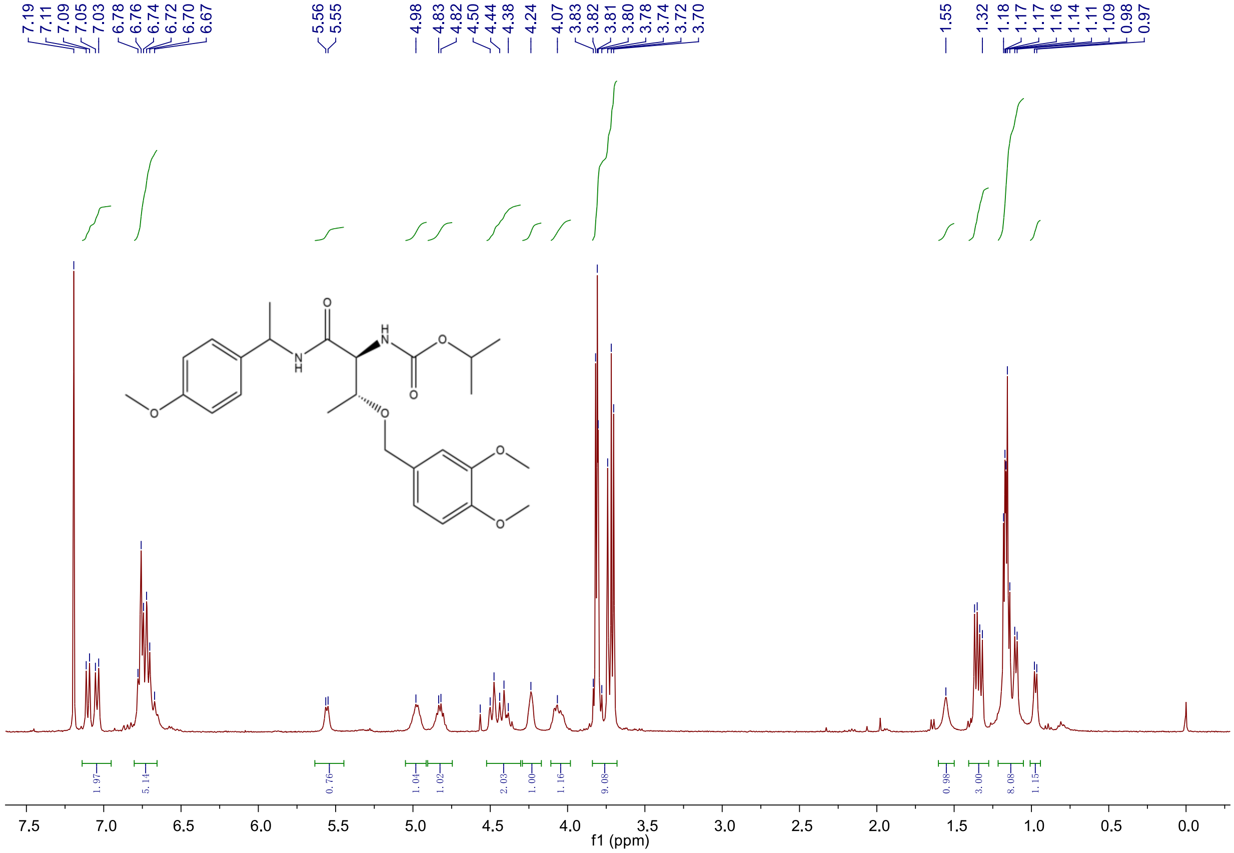

Figure 49 ^{1}HNMR spectrum of **I-21**

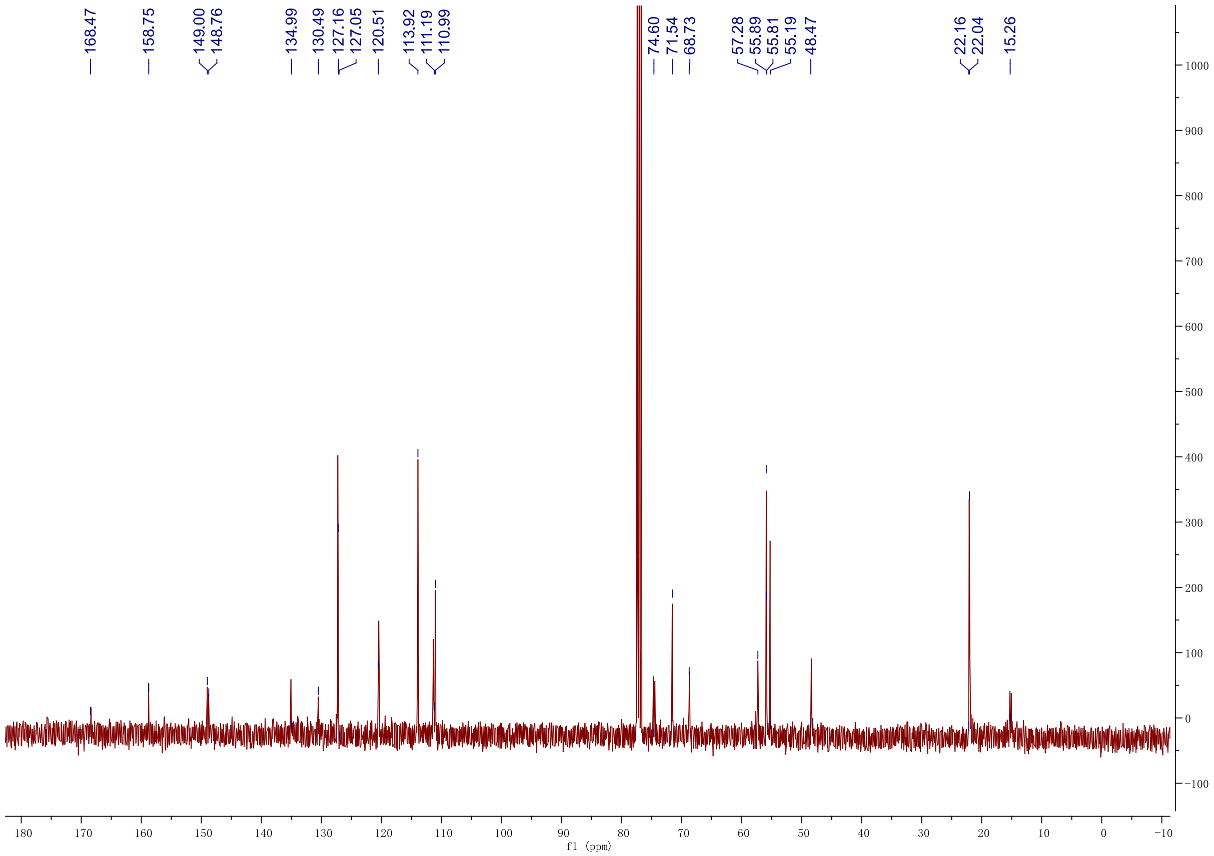

Figure 50 ^{13}C NMR spectrum of **I-21**

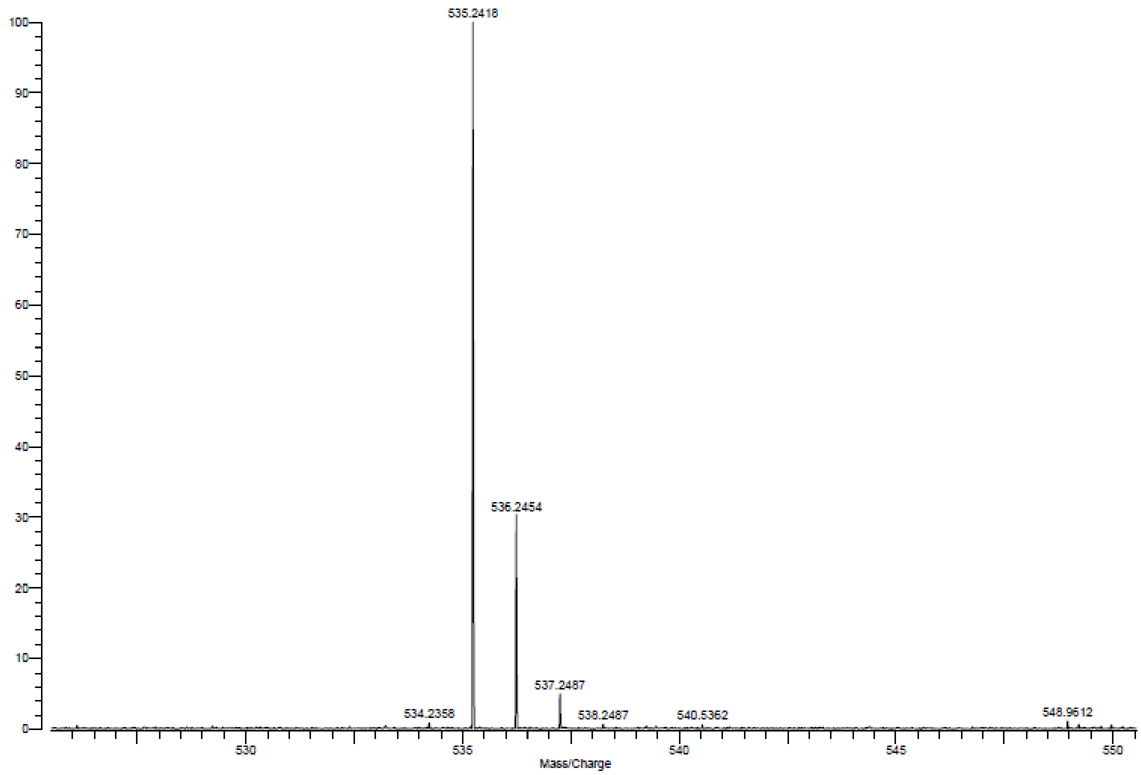

Figure 51 HRMS of **I-21**

I-22

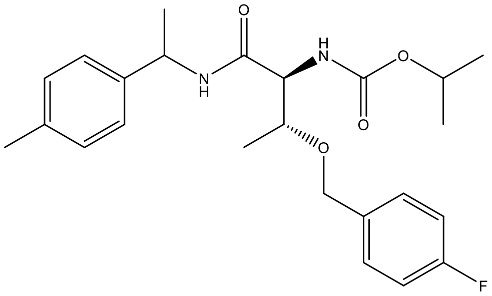

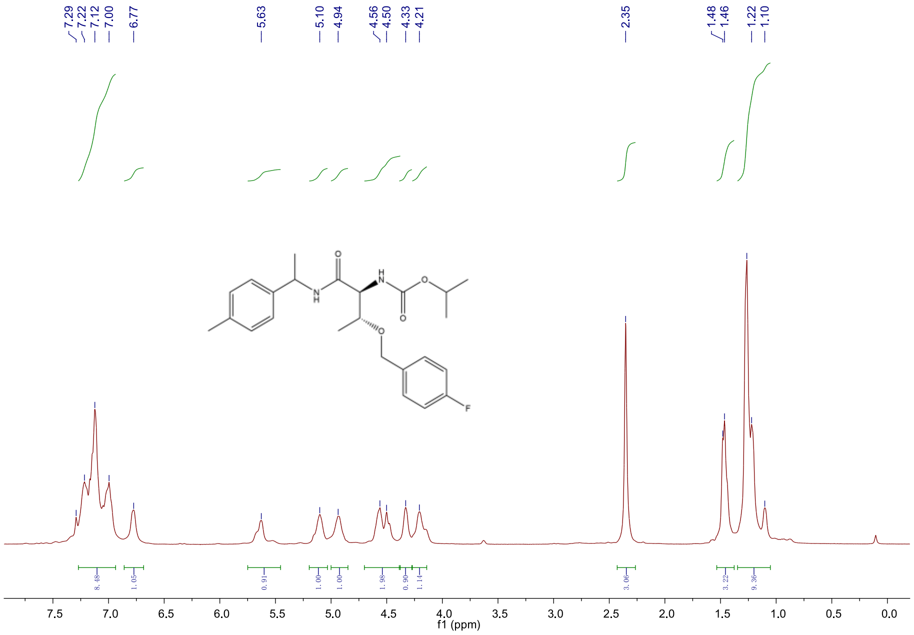

Figure 52 ^{1}HNMR spectrum of **I-22**

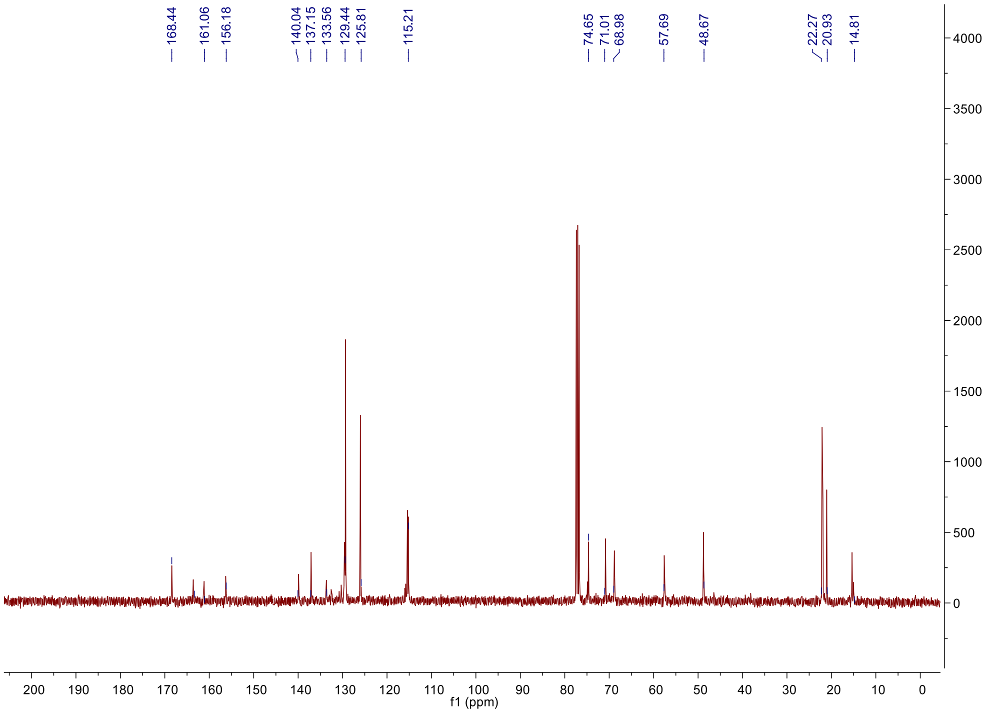

Figure 53 ^{13}C NMR spectrum of **I-22**

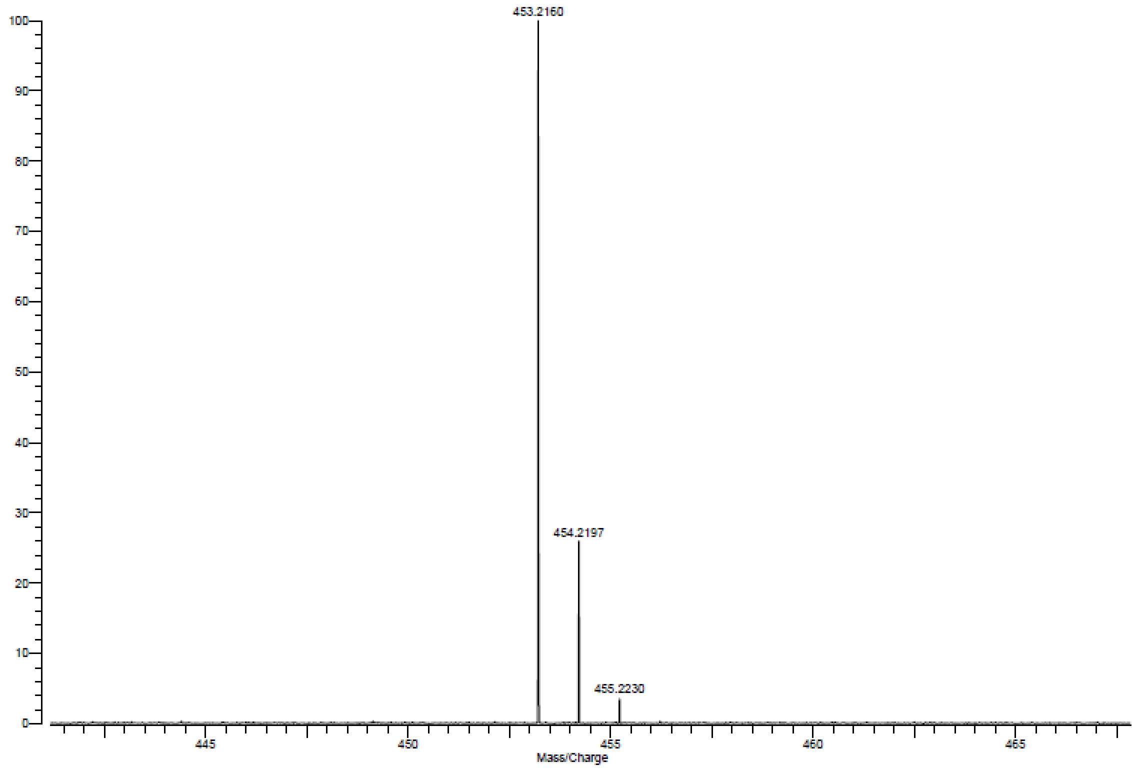

Figure 54 HRMS of **I-22**

I-23

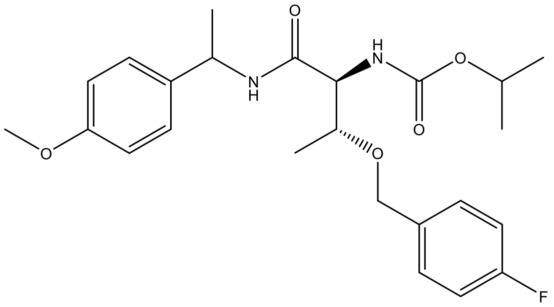

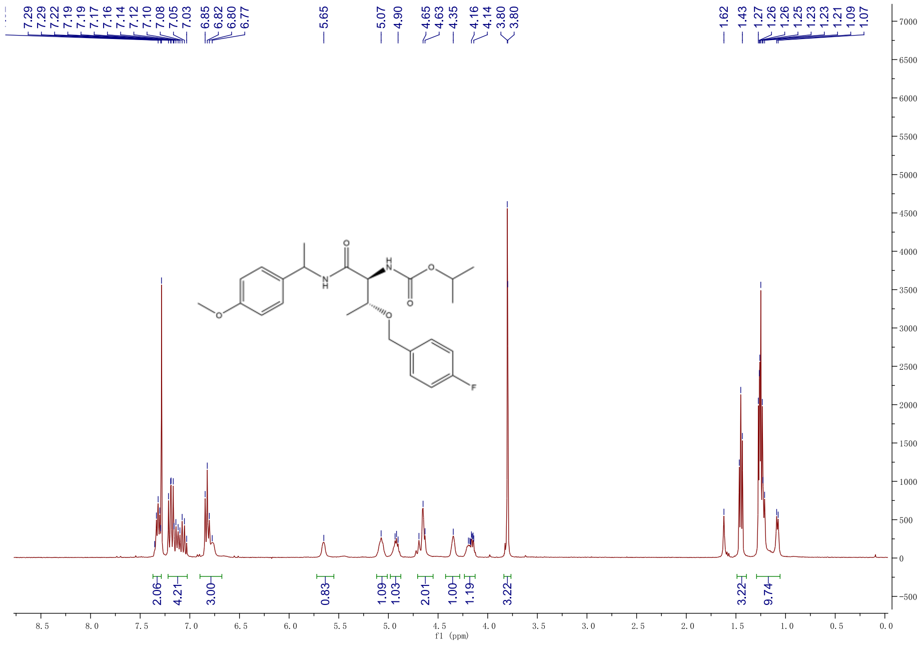

Figure 55 ^{1}HNMR spectrum of **I-23**

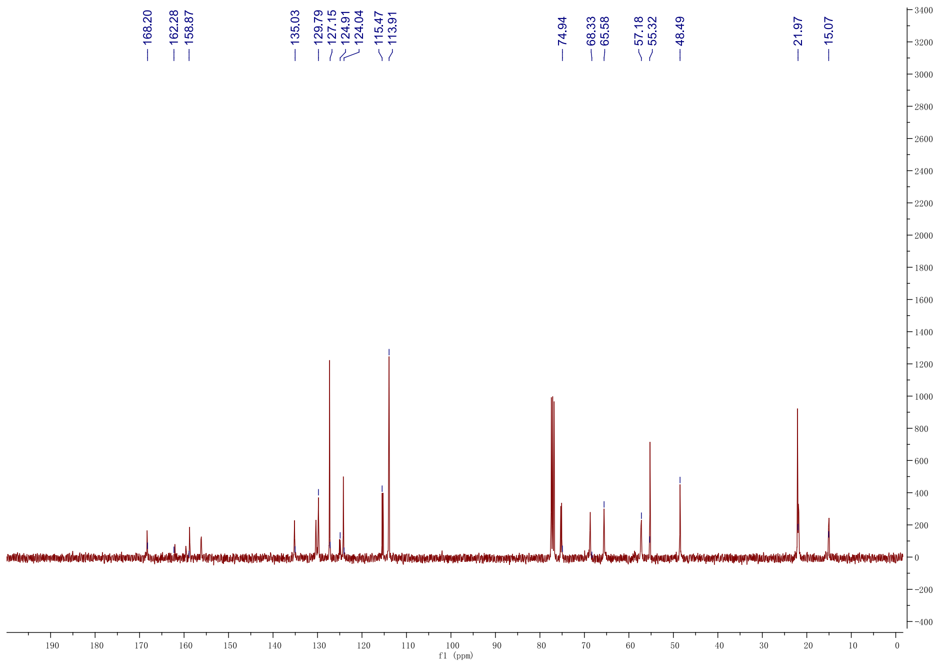

Figure 56 ^{13}C NMR spectrum of **I-23**

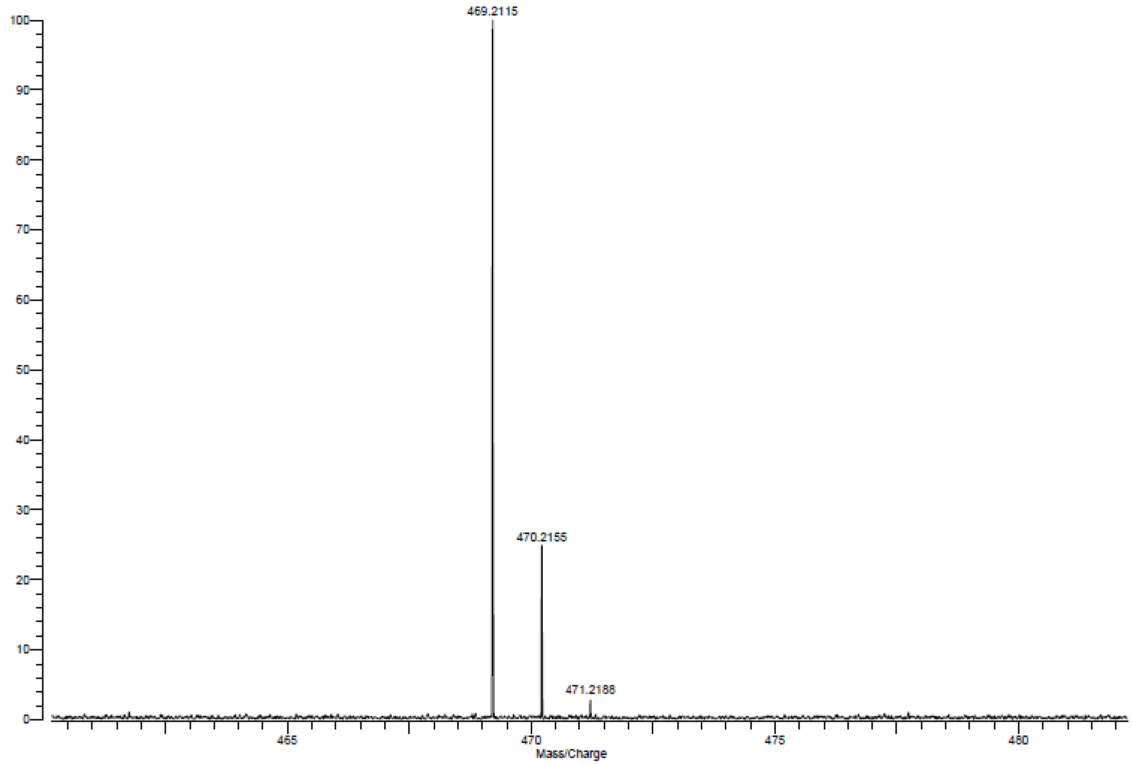

Figure 57 HRMS of **I-23**

I-24

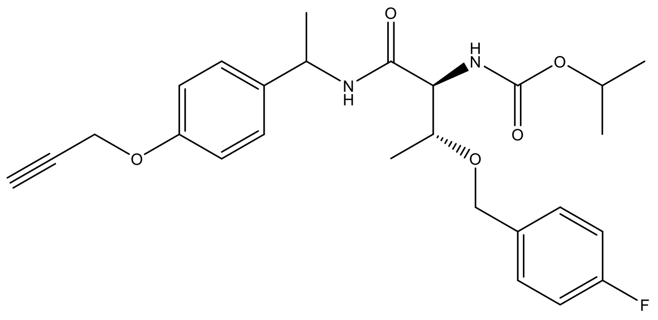

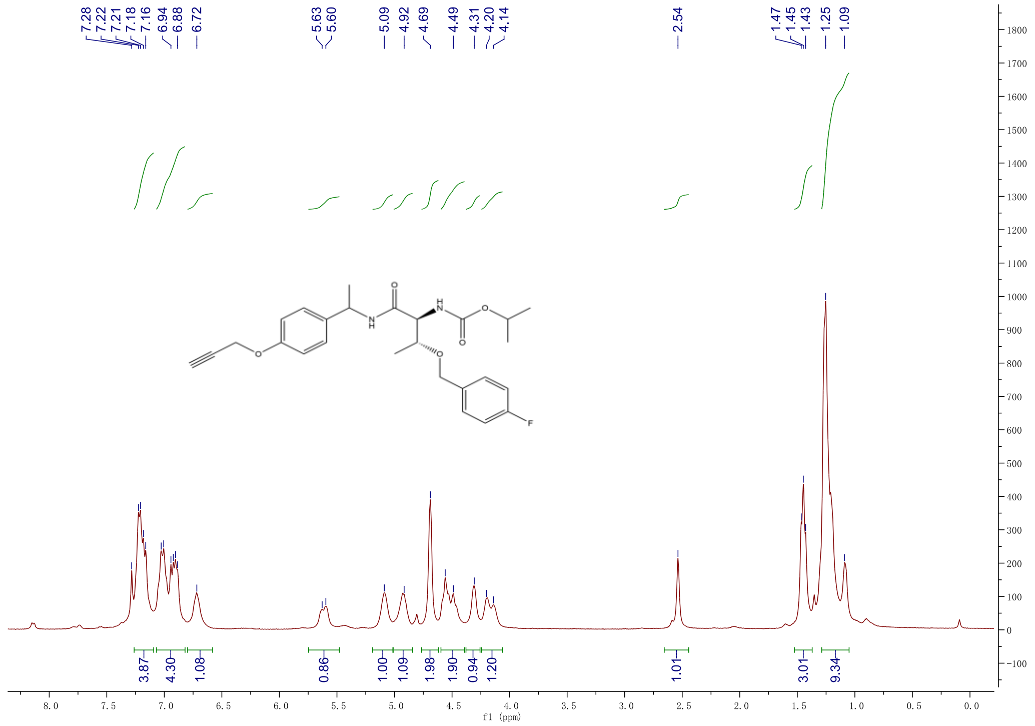

Figure 58 ^{1}HNMR spectrum of **I-24**

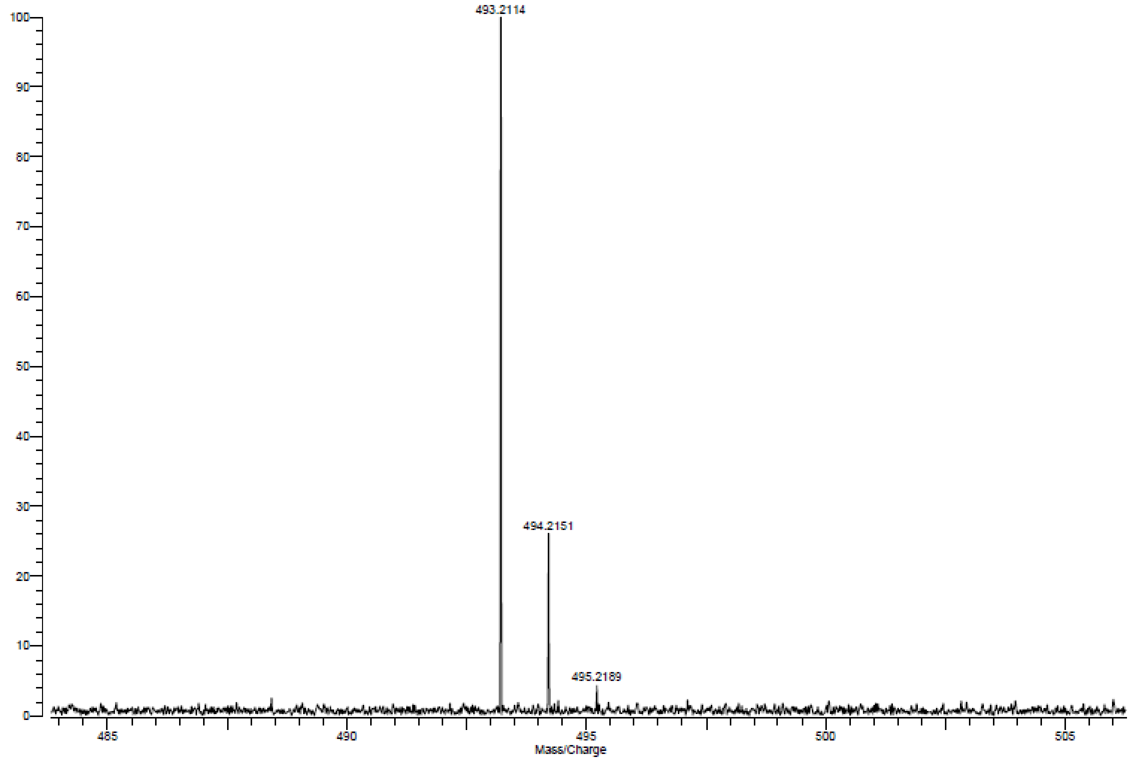

Figure 59 HRMS of **I-24**

I-25

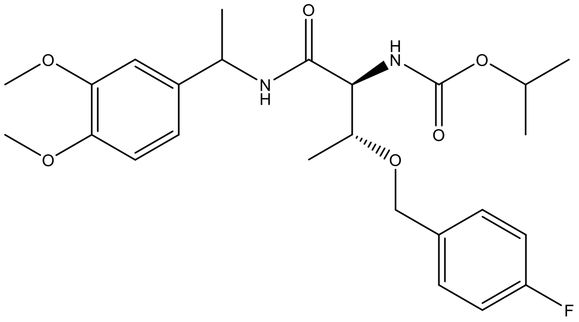

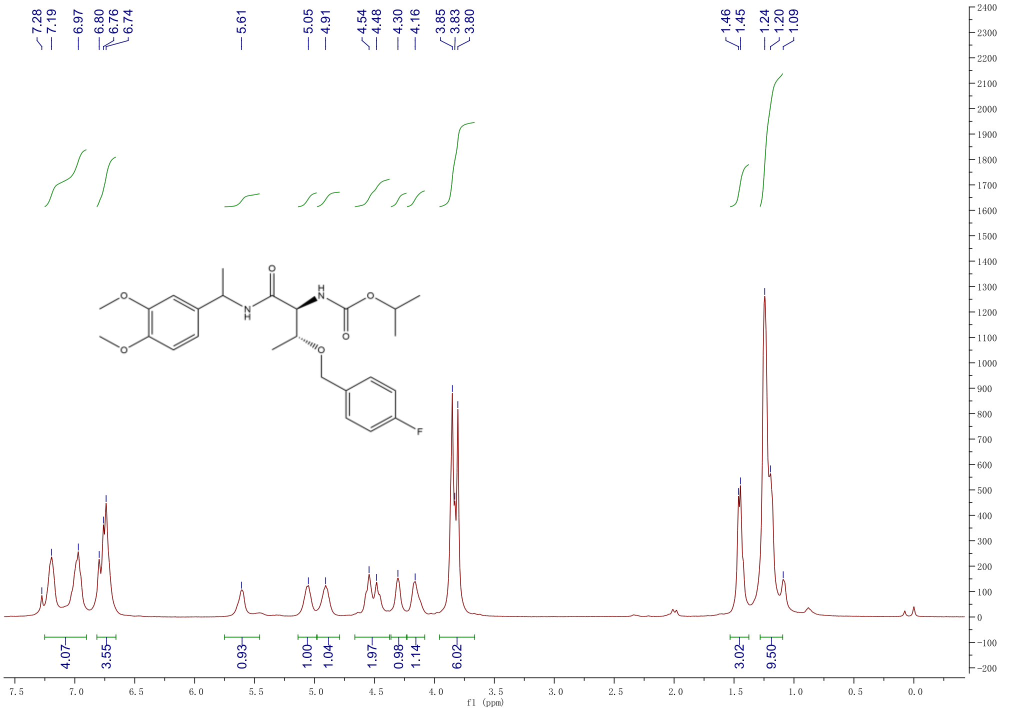

Figure 60 ^{1}HNMR spectrum of **I-25**

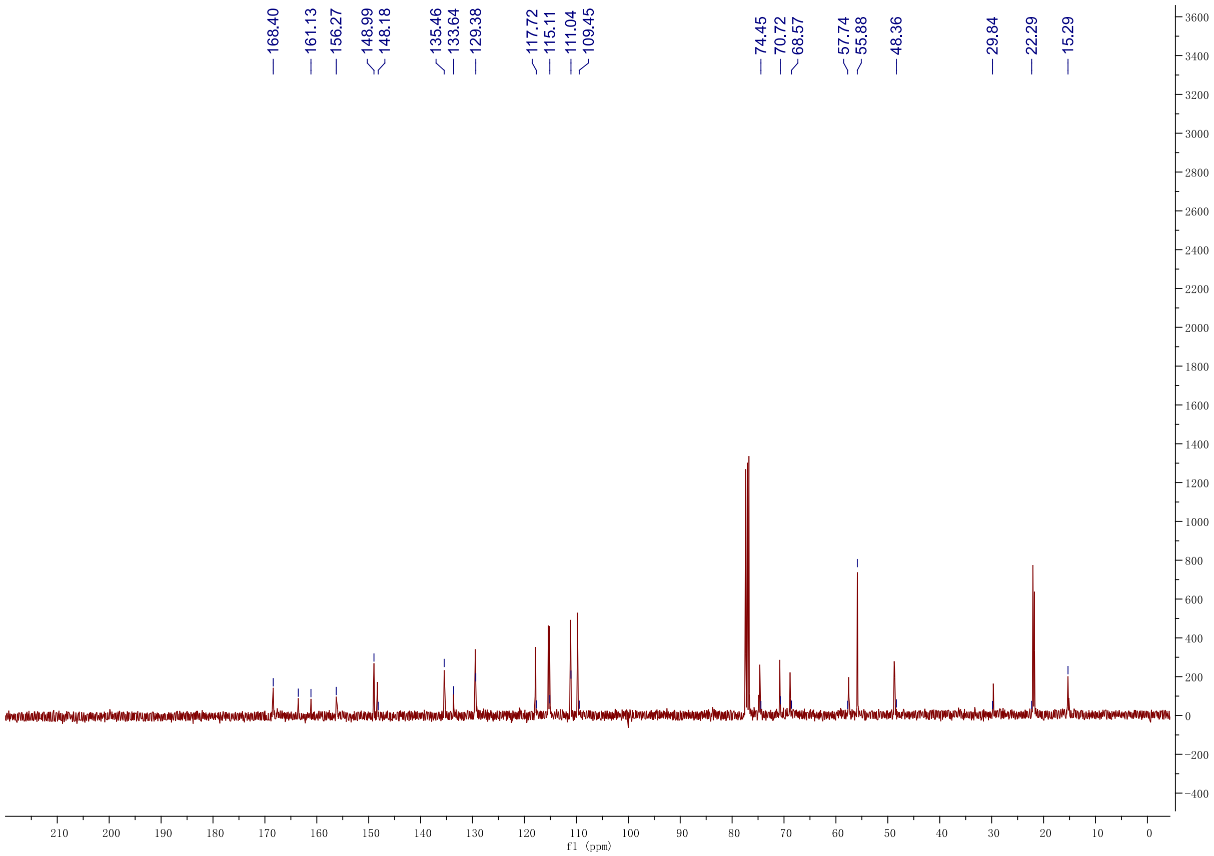

Figure 61 ^{13}C NMR spectrum of **I-25**

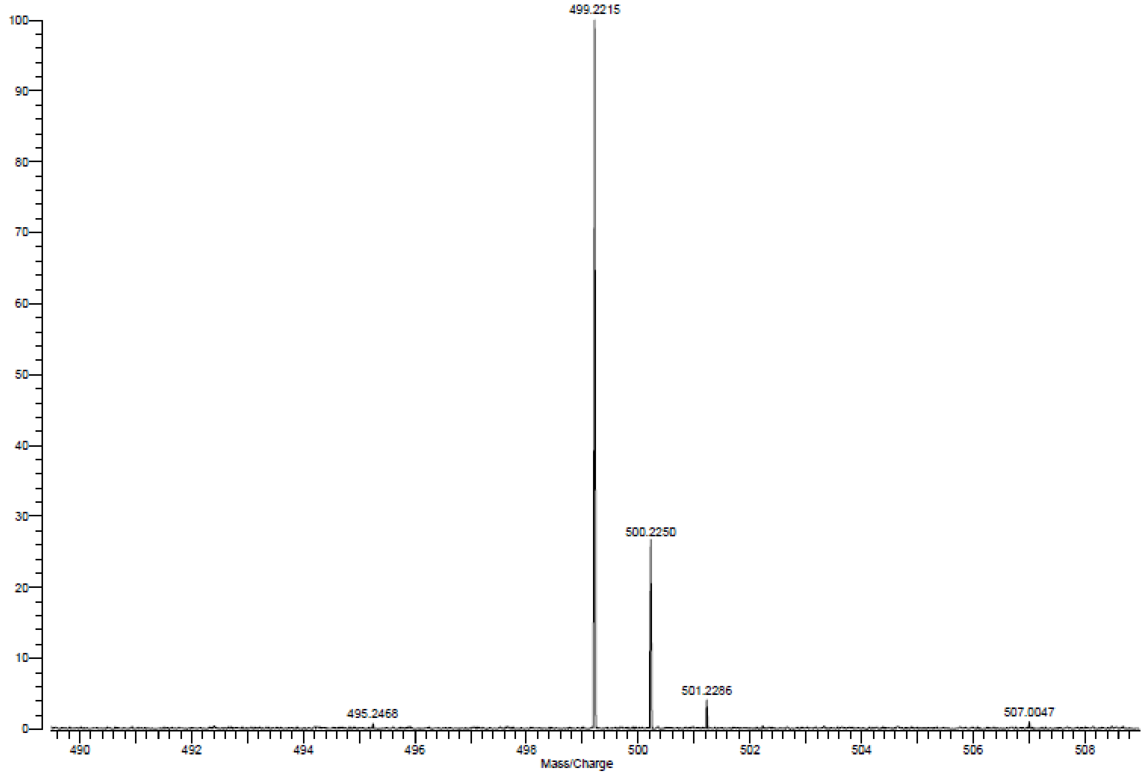

Figure 62 HRMS of **I-25**

I-26

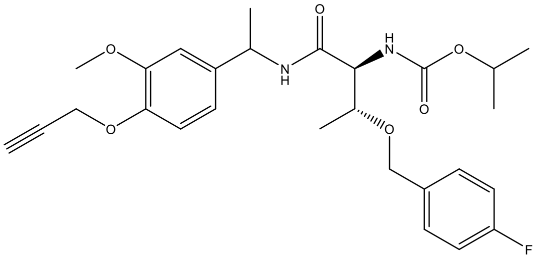

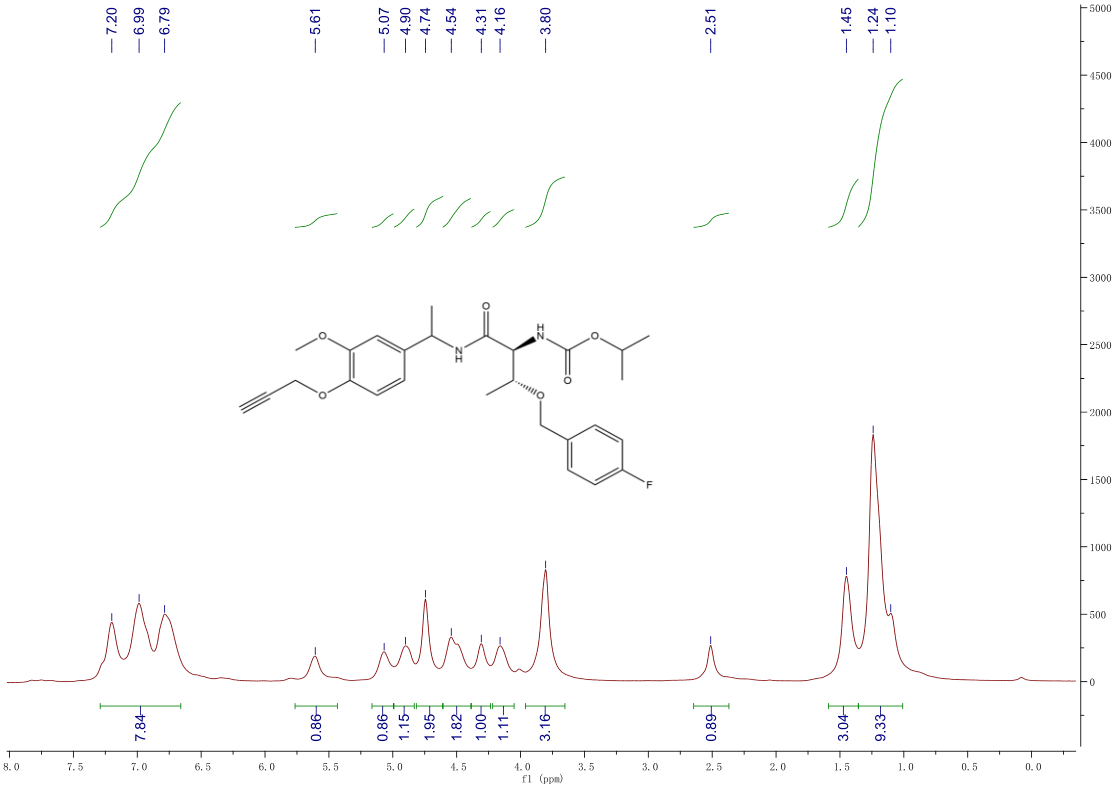

Figure 63 ^{1}HNMR spectrum of **I-26**

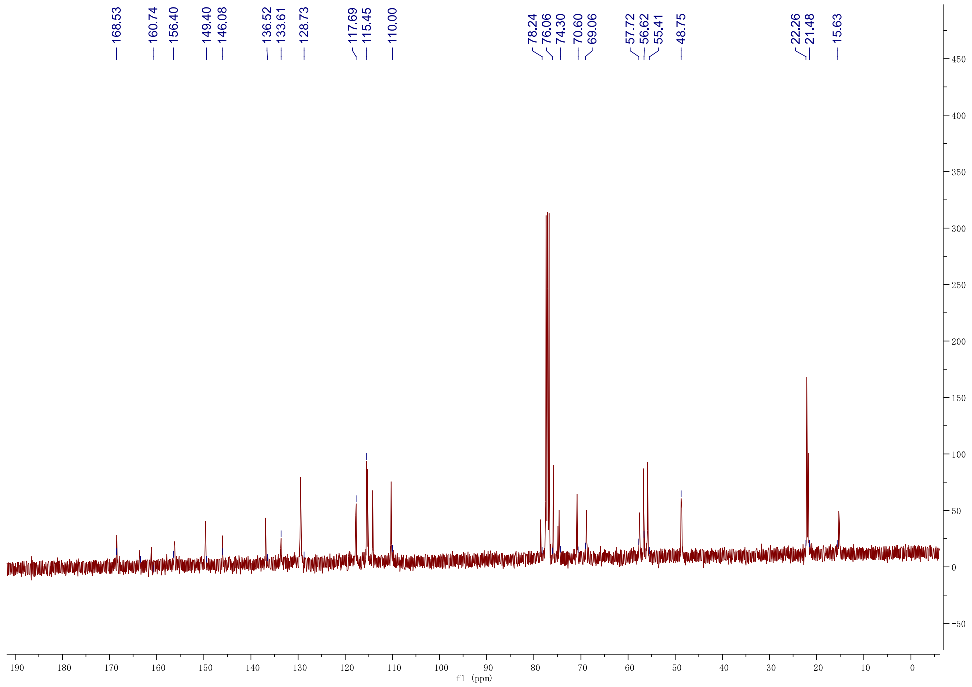

Figure 64 ^{13}C NMR spectrum of **I-26**

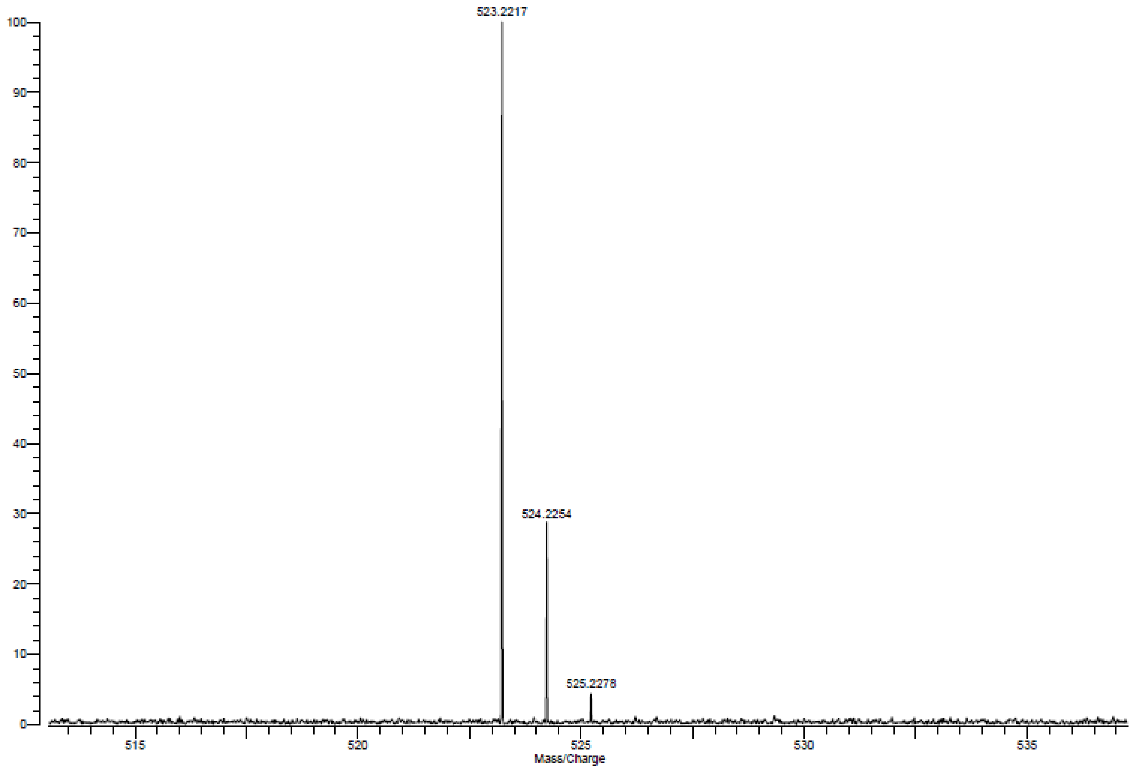

Figure 65 HRMS of **I-26**

I-27

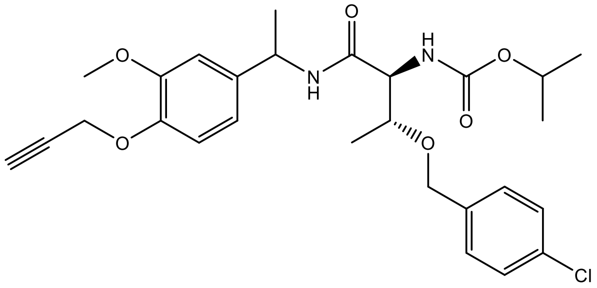

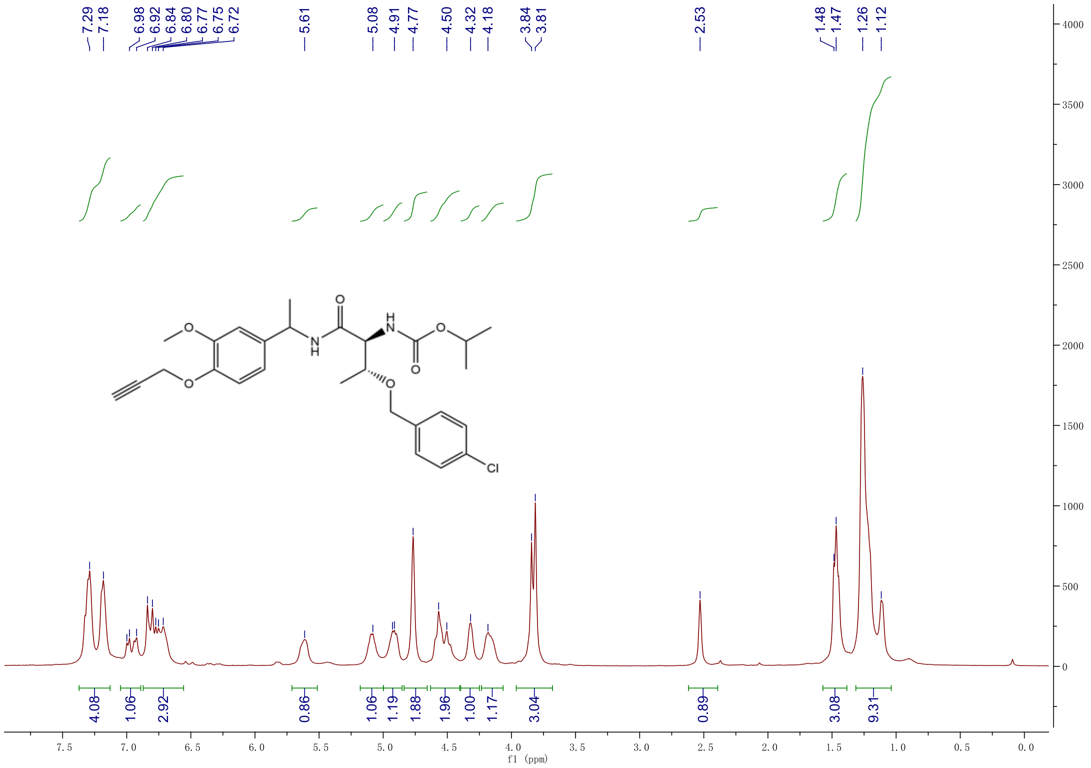

Figure 66 ^{1}HNMR spectrum of **I-27**

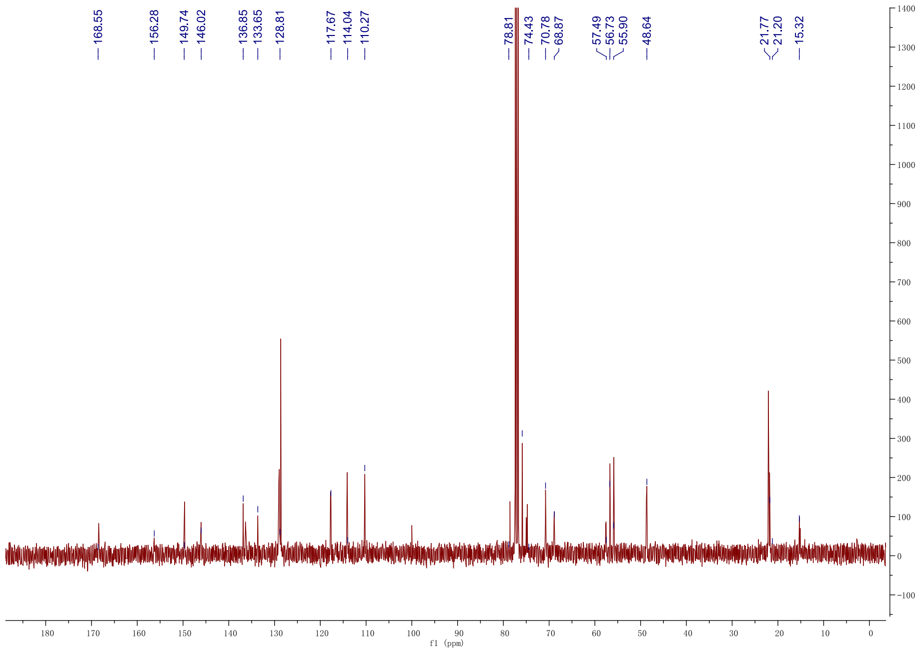

Figure 67 ^{13}C NMR spectrum of **I-27**

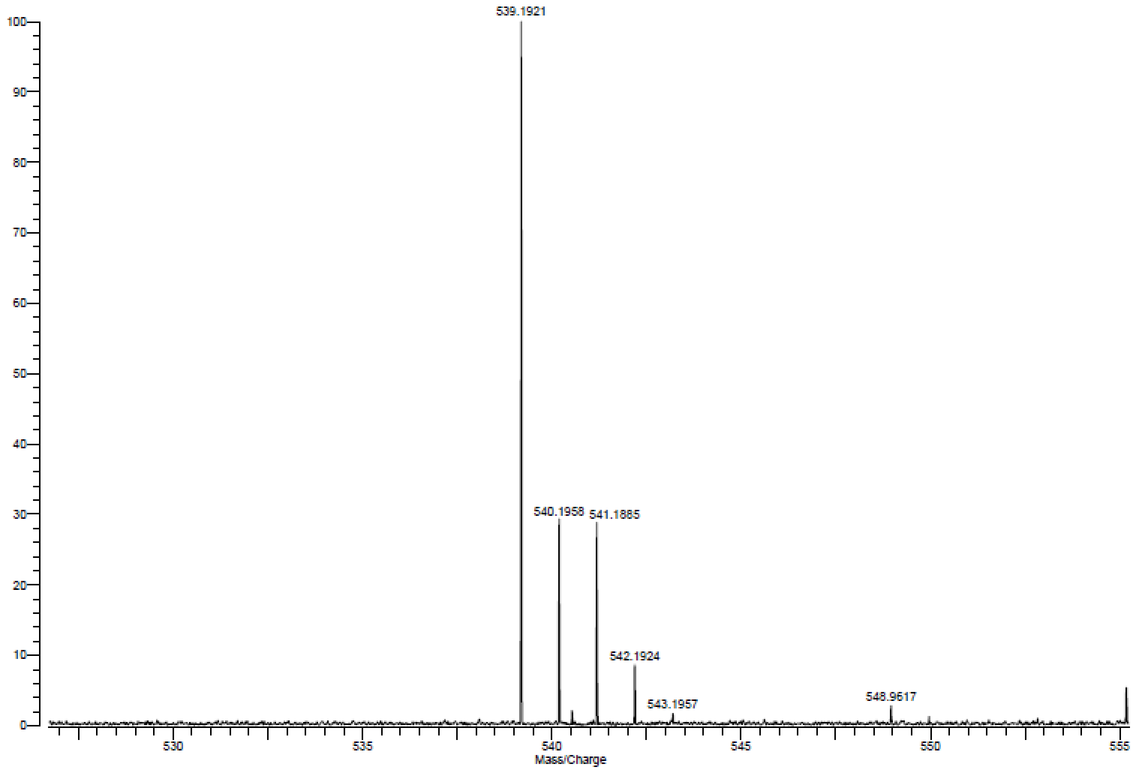

Figure 68 HRMS of **I-27**

I-28

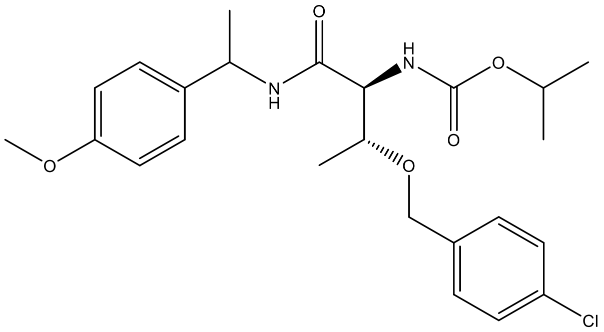

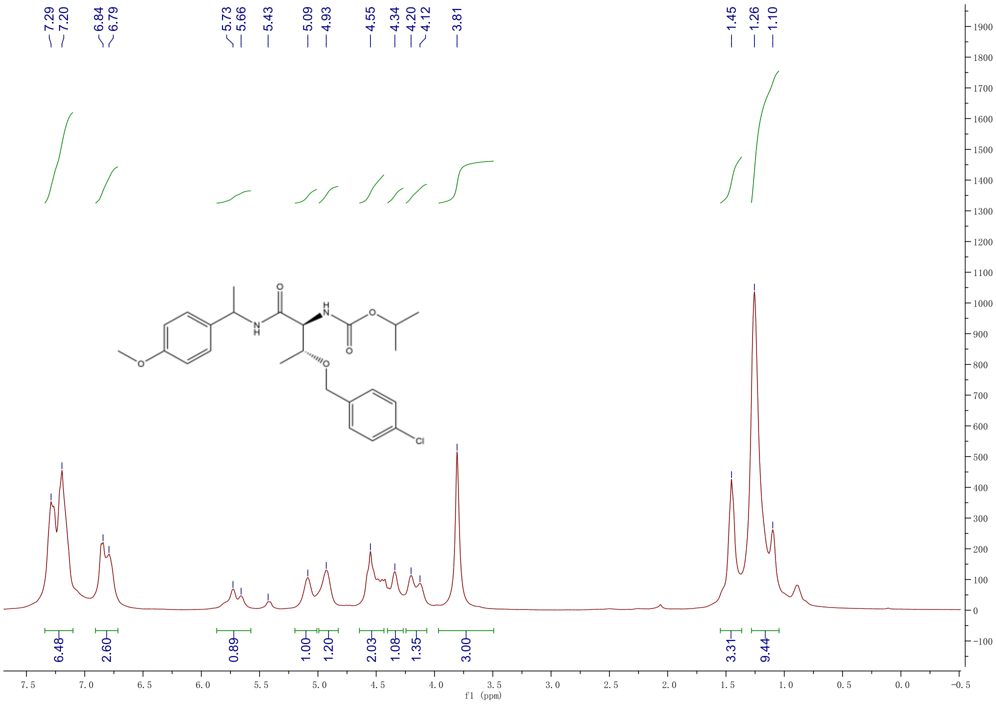

Figure 69 ^{1}HNMR spectrum of **I-28**

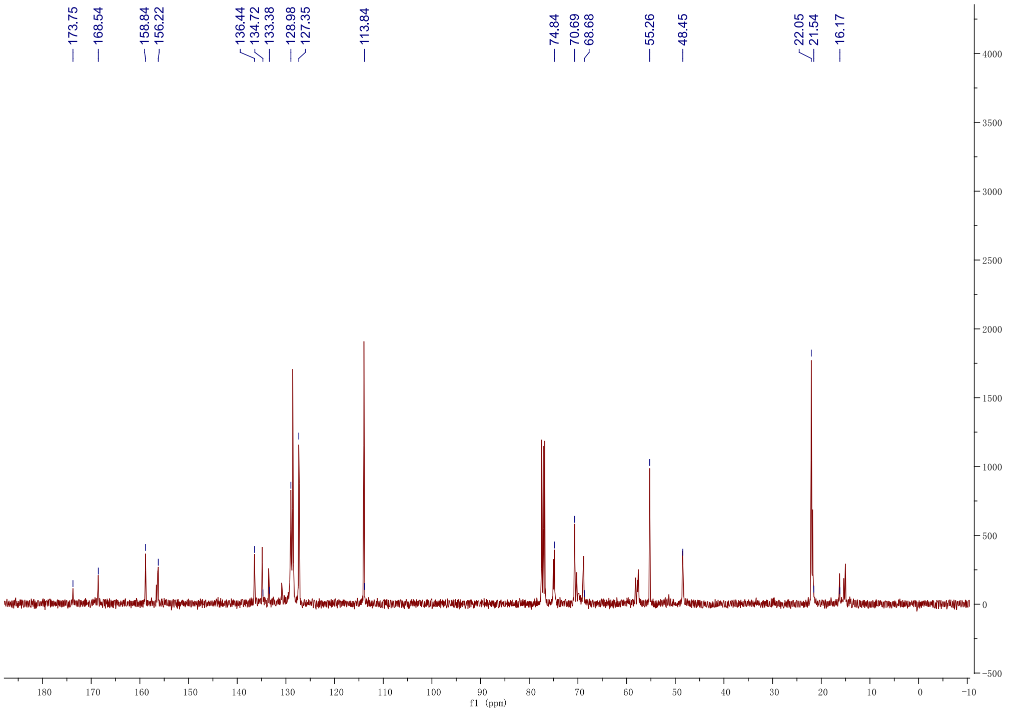

Figure 70 ^{13}C NMR spectrum of **I-28**

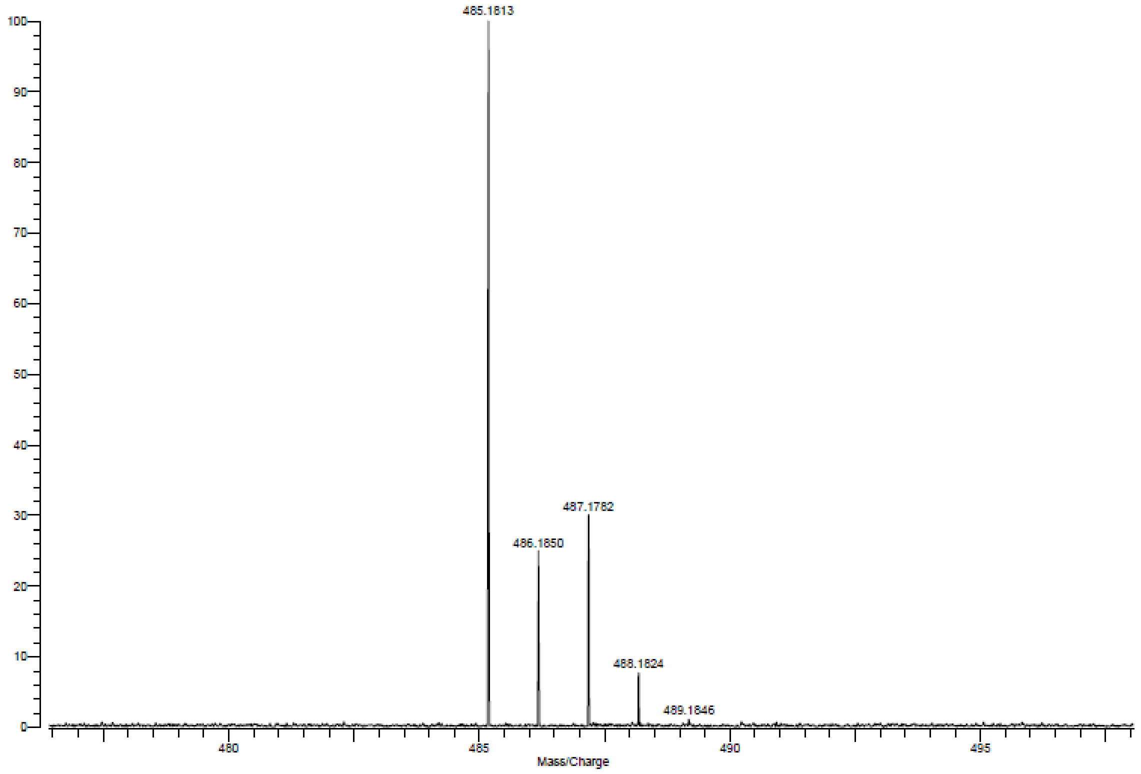

Figure 71 HRMS of **I-28**

I-29

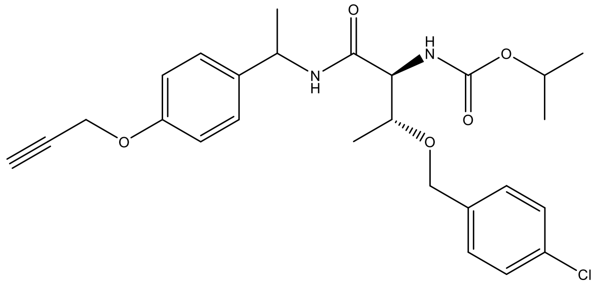

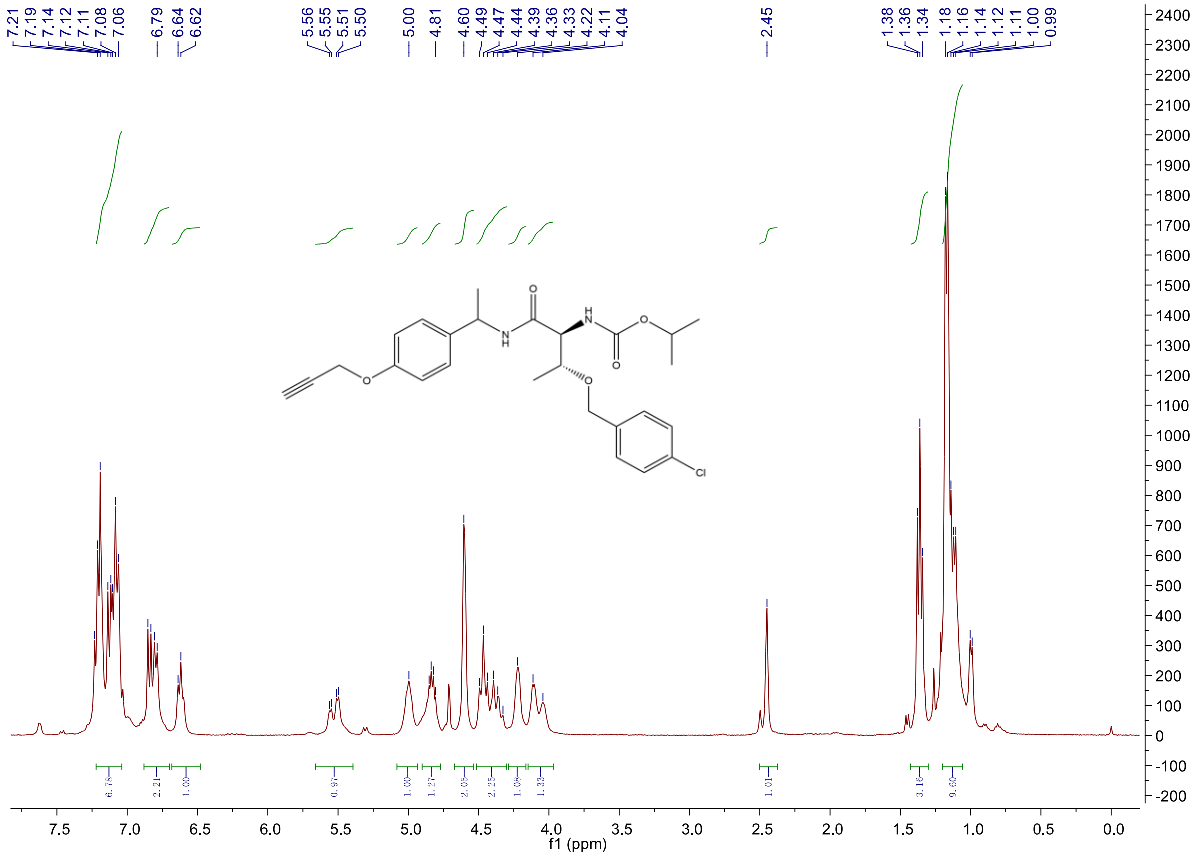

Figure 72 ^{1}HNMR spectrum of **I-29**

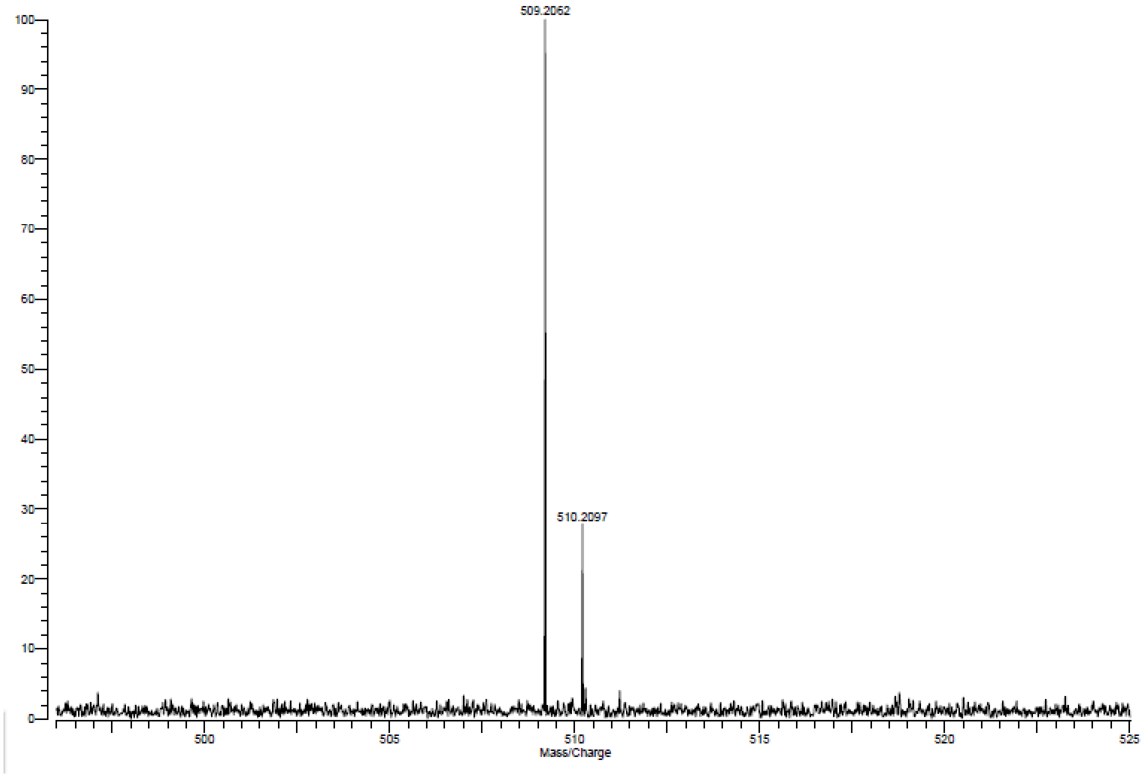

Figure 73 HRMS of **I-29**

I-30

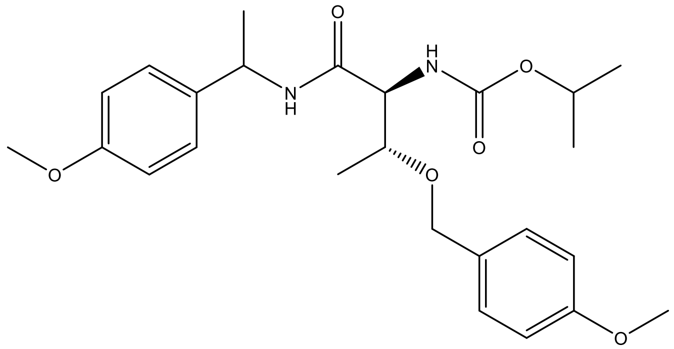

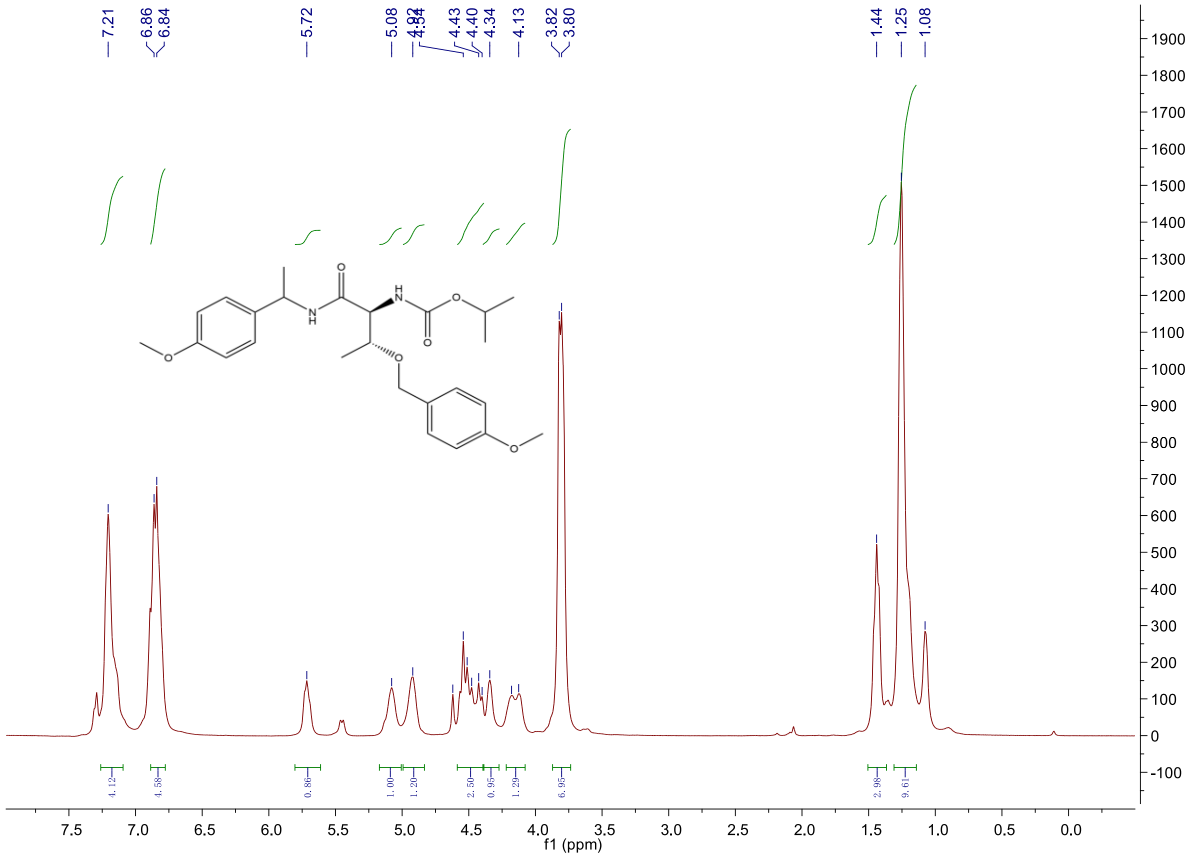

Figure 74 ^{1}HNMR spectrum of **I-30**

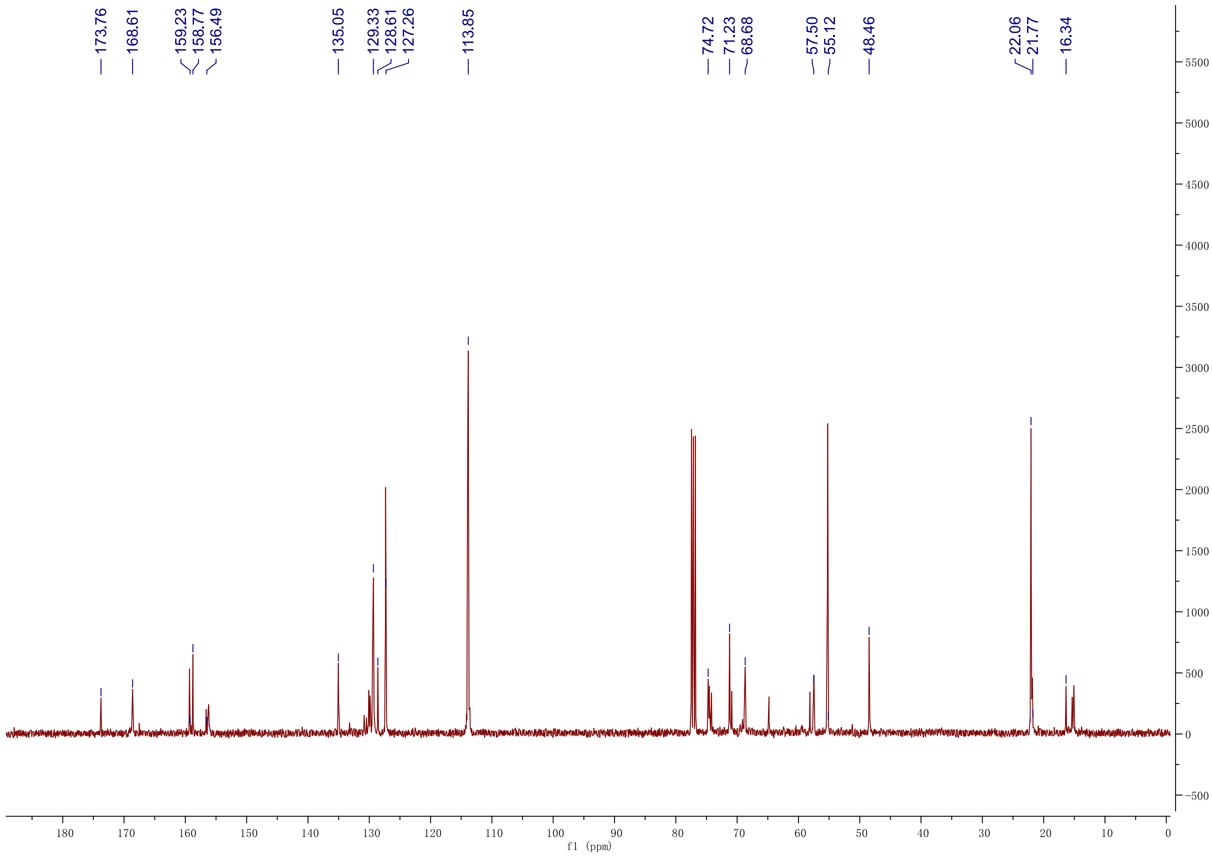

Figure 75 ^{13}C NMR spectrum of **I-30**

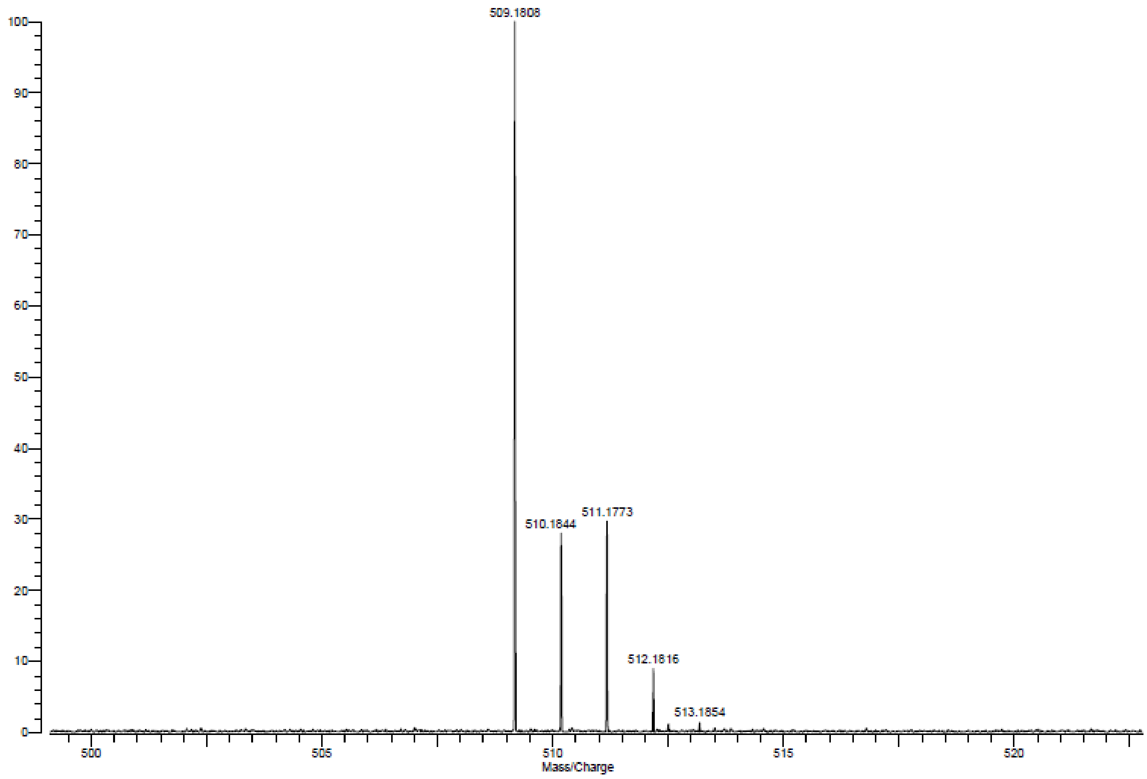

Figure 76 HRMS of **I-30**

I-31

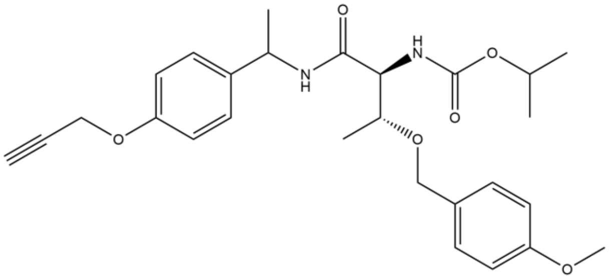

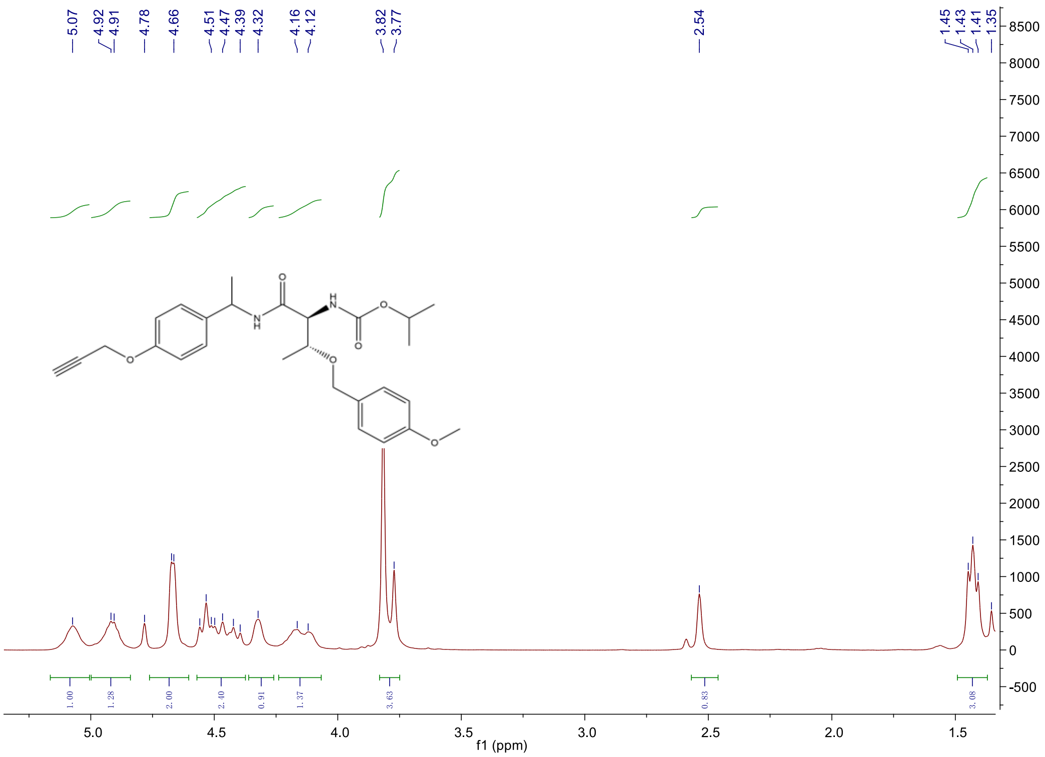

Figure 77 ^{1}HNMR spectrum of **I-31**

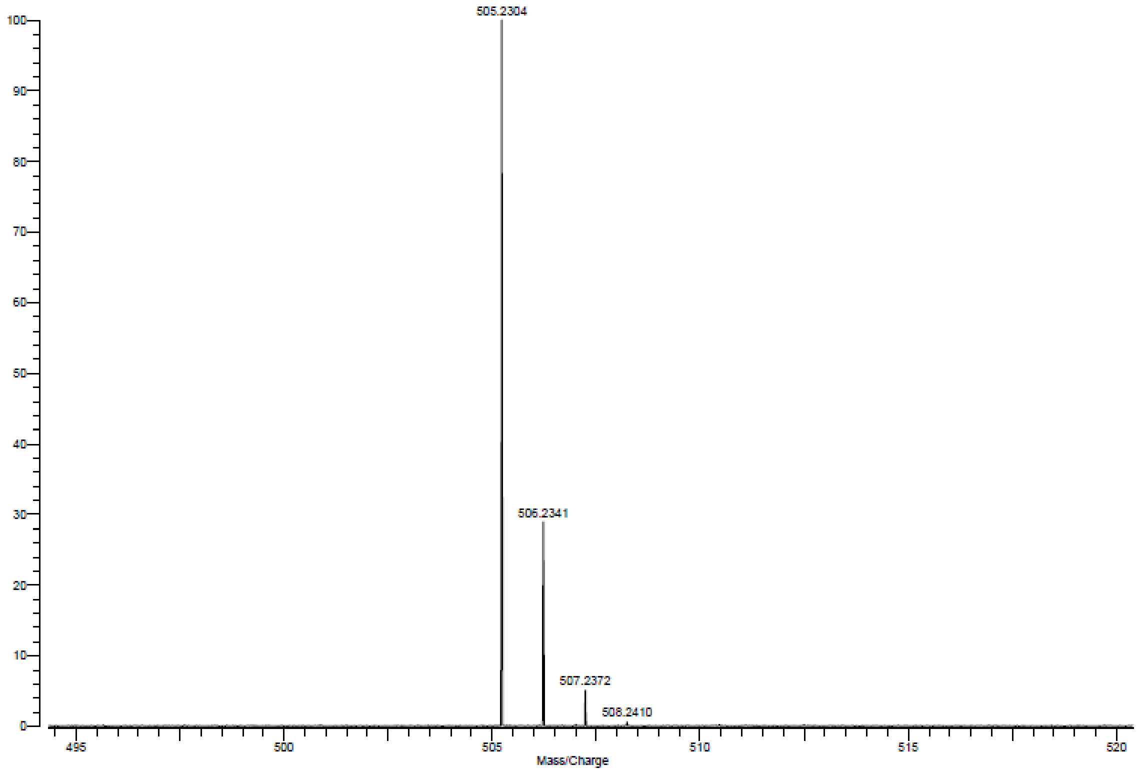

Figure 78 HRMS of **I-31**

I-32

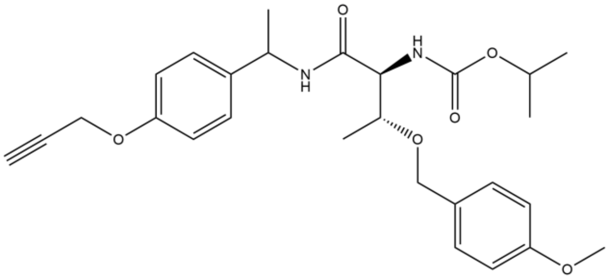

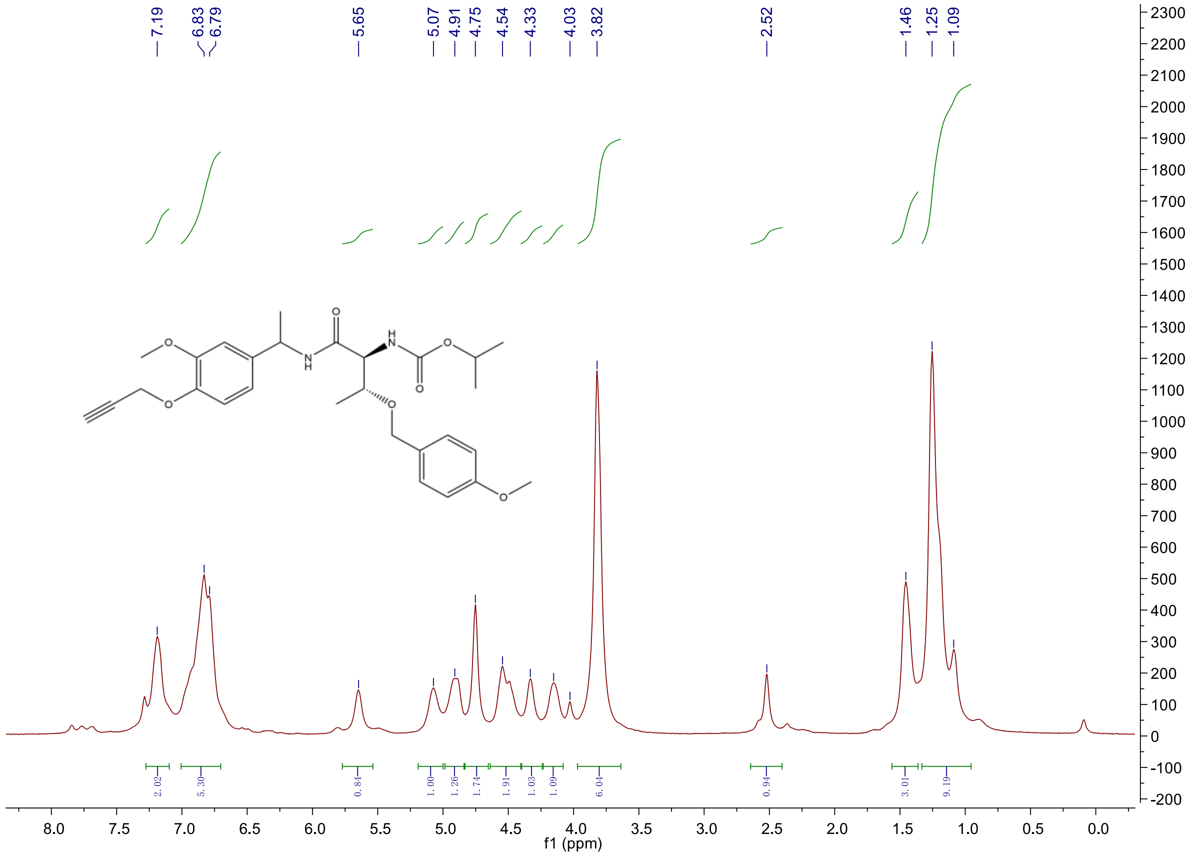

Figure 79 ^{1}HNMR spectrum of **I-32**

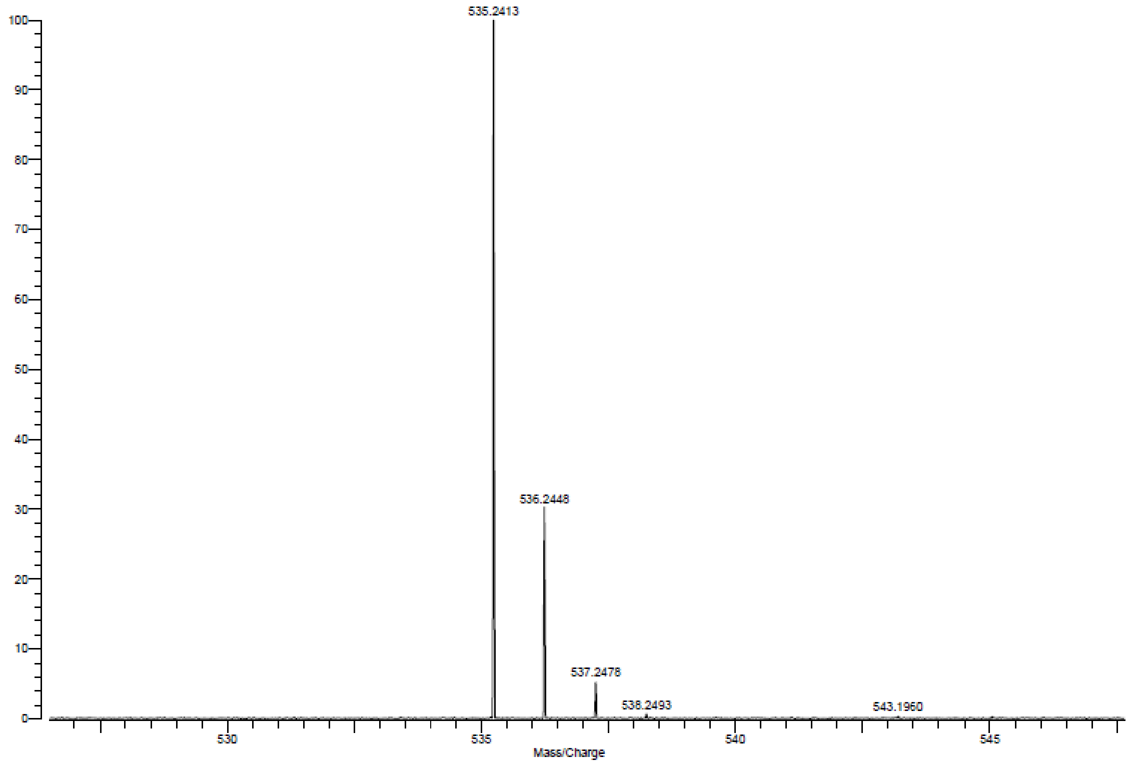

Figure 80 HRMS of **I-32**

I-33

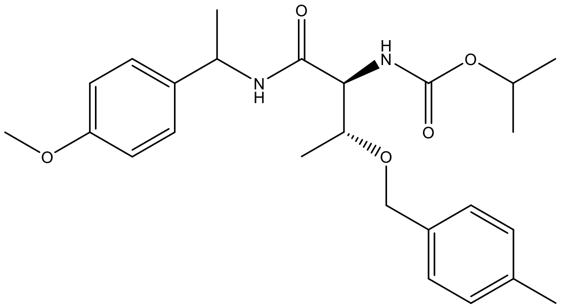

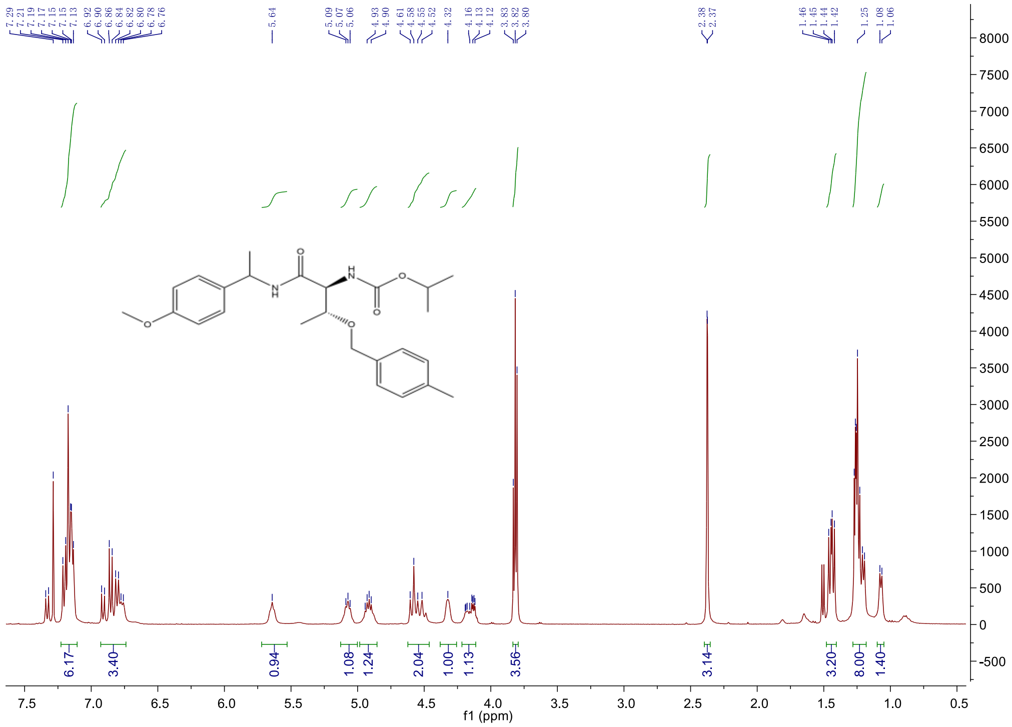

Figure 81 ^{1}HNMR spectrum of **I-33**

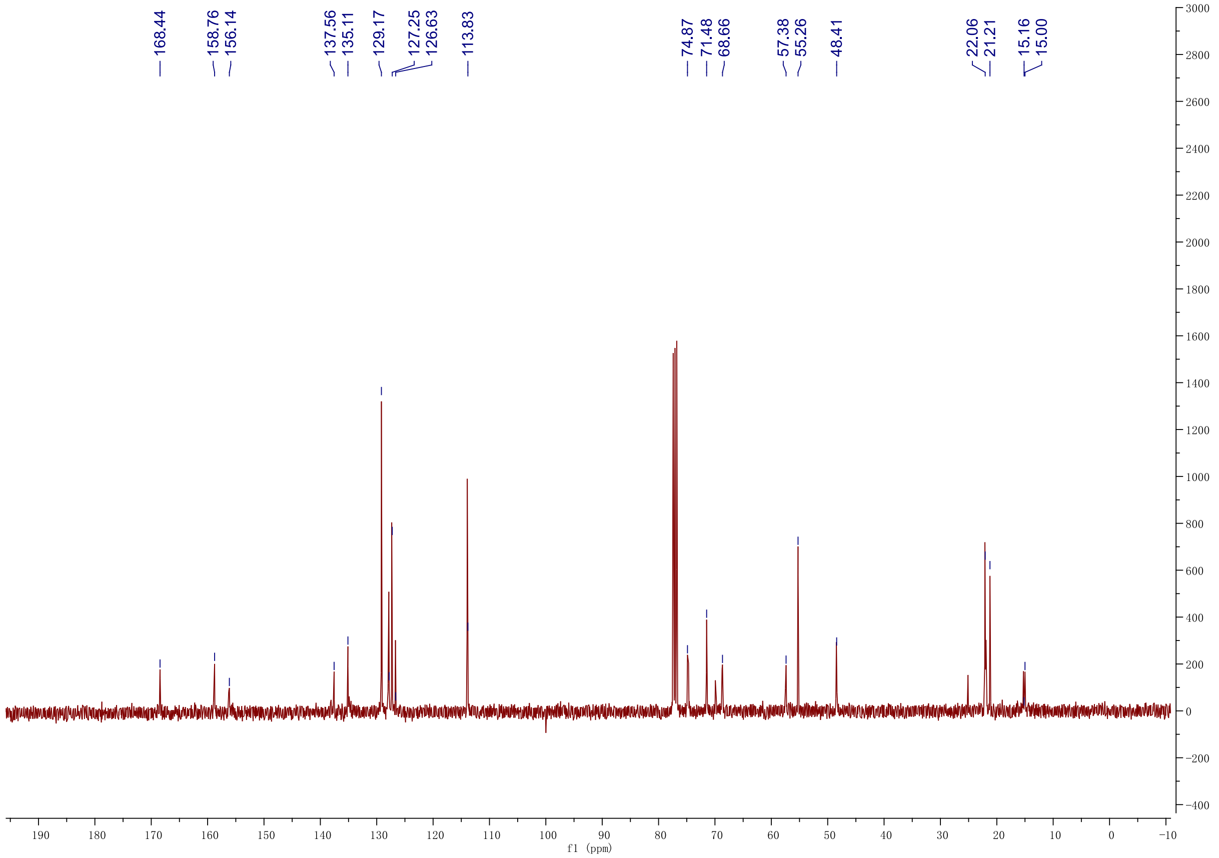

Figure 82 ^{13}C NMR spectrum of **I-33**

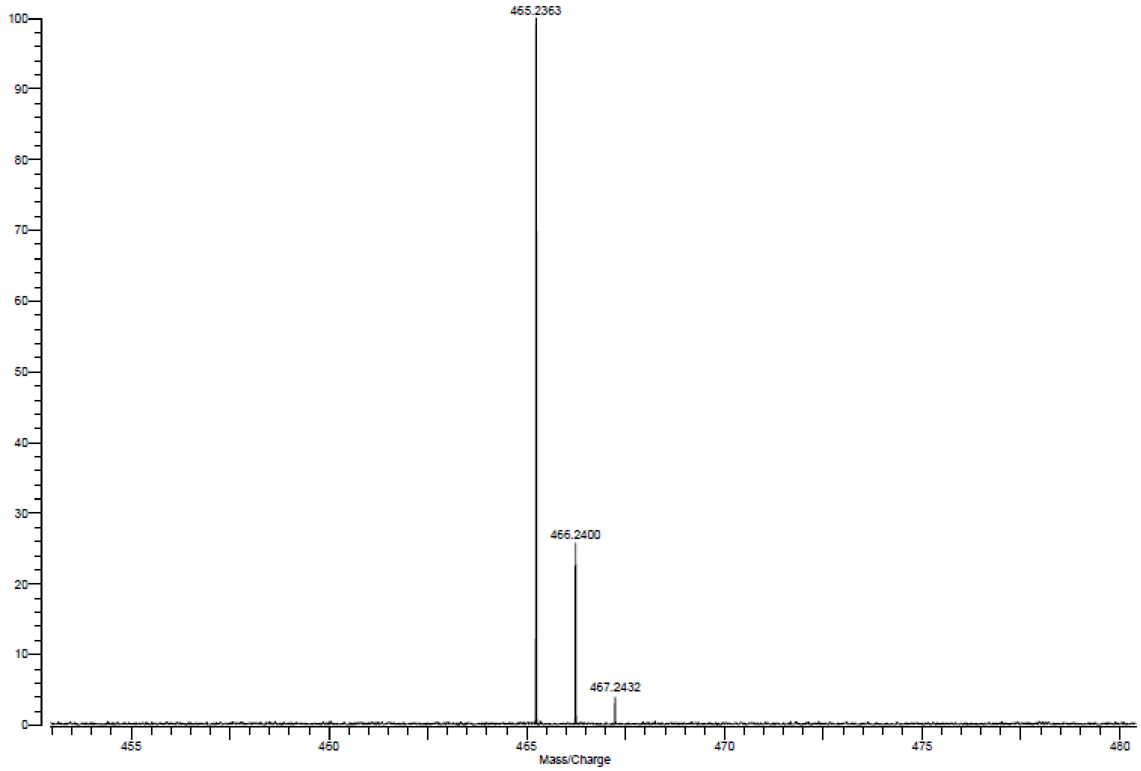

Figure 83 HRMS of **I-33**

I-34

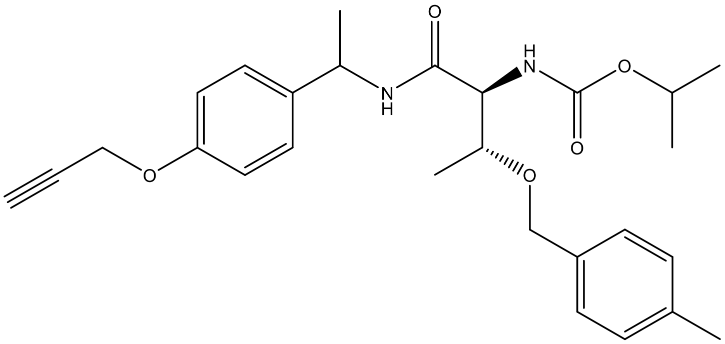

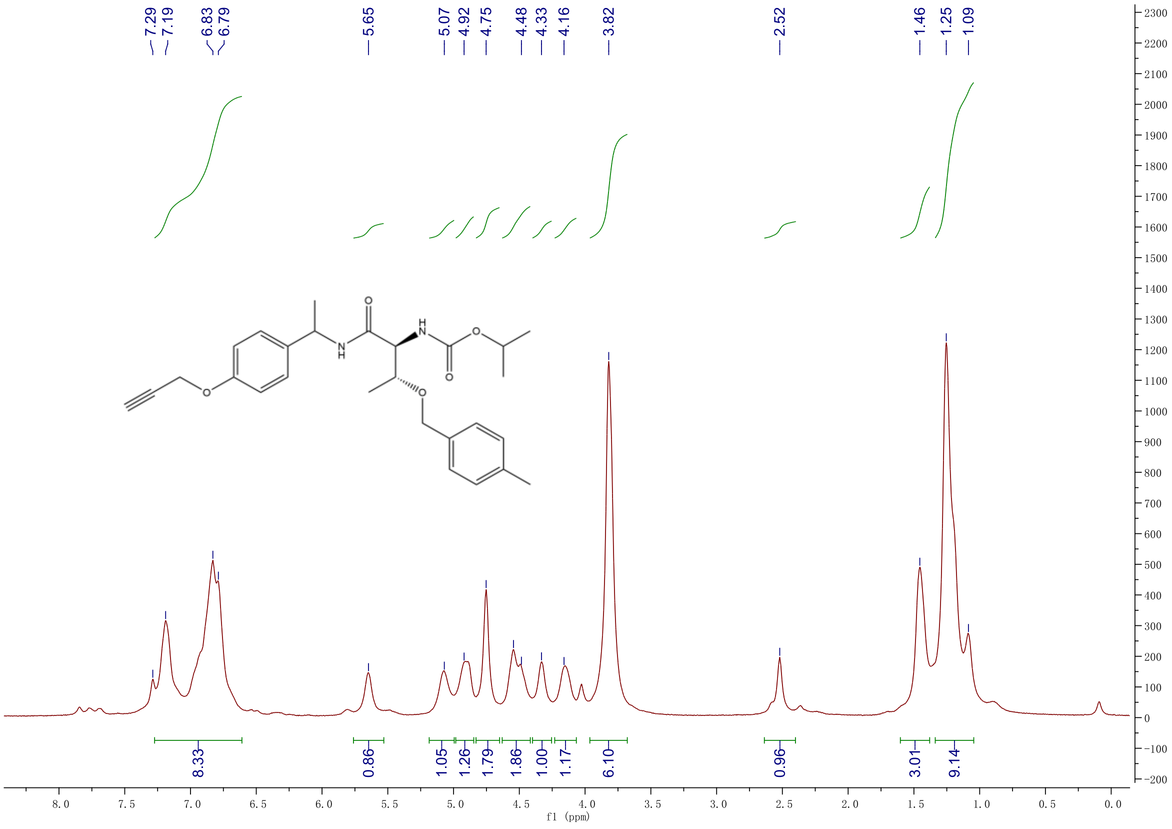

Figure 84 ^{1}HNMR spectrum of **I-34**

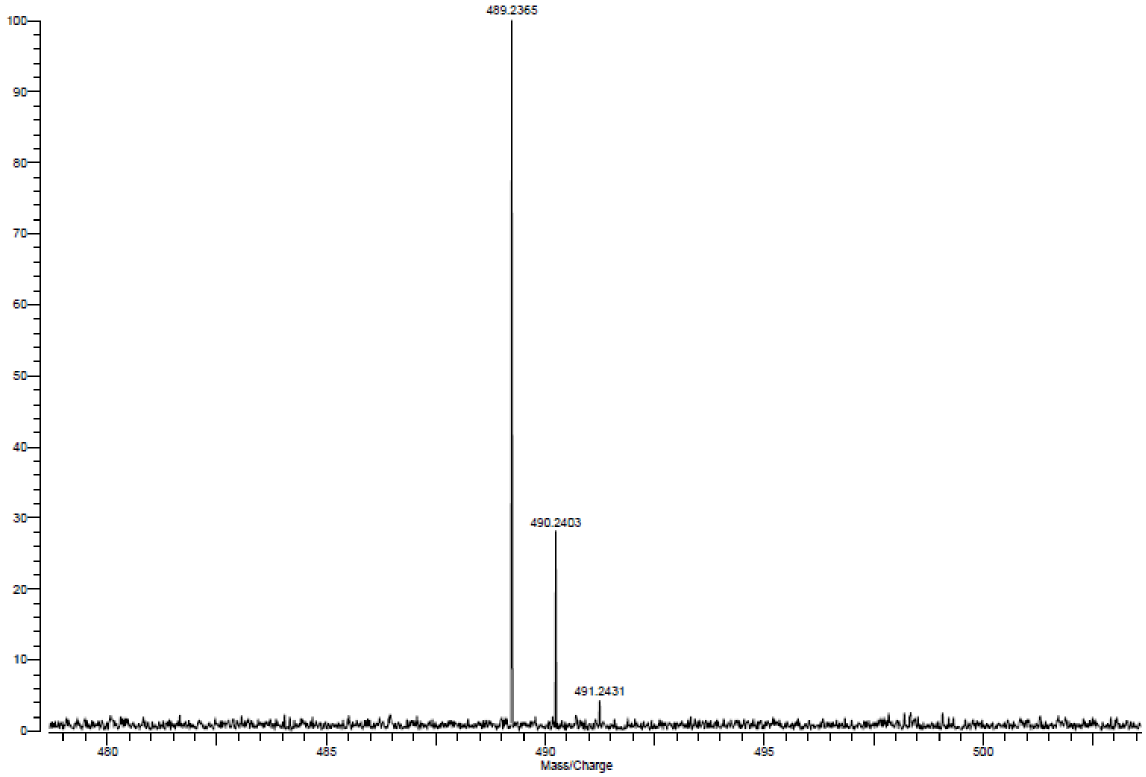

Figure 85 HRMS of **I-34**

I-35

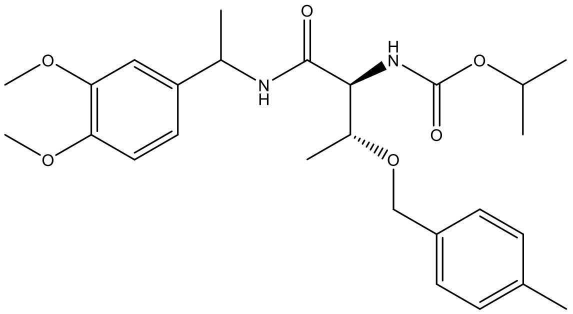

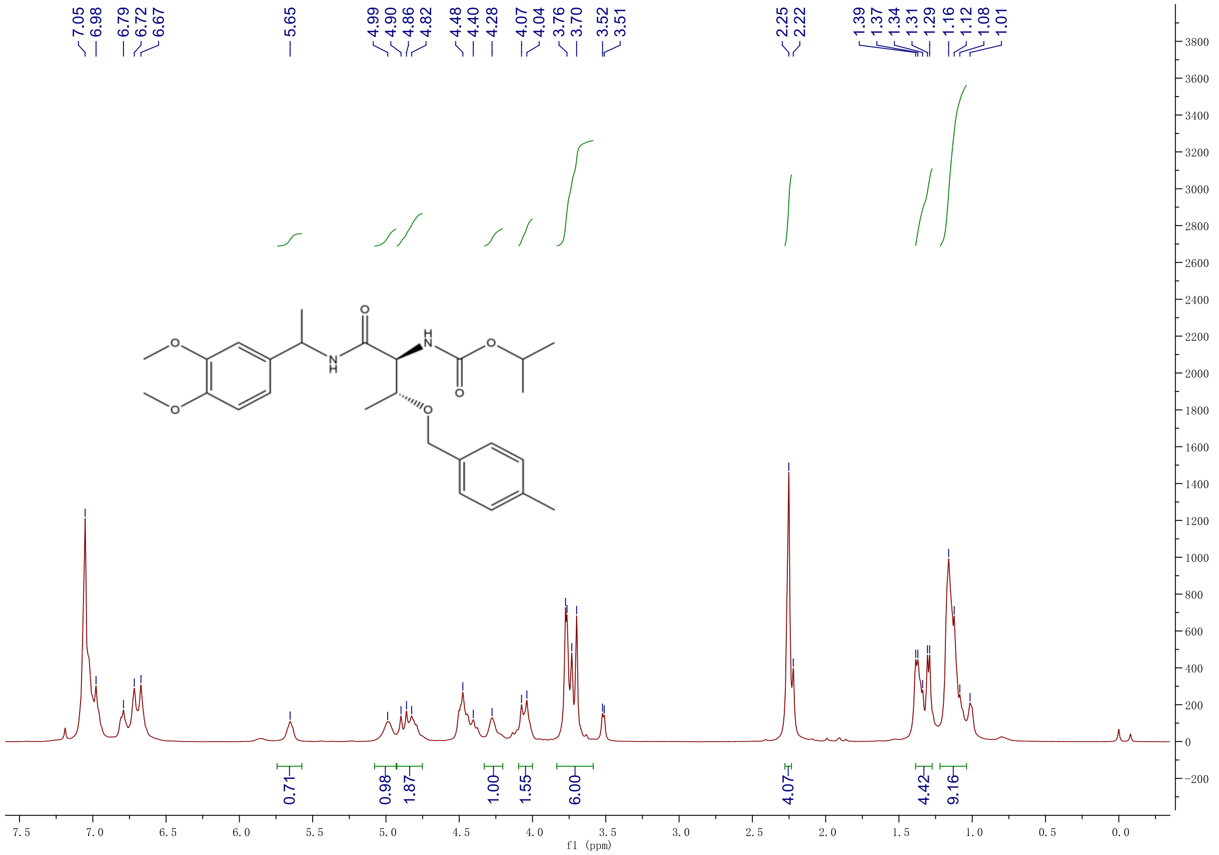

Figure 86 ^{1}HNMR spectrum of **I-35**

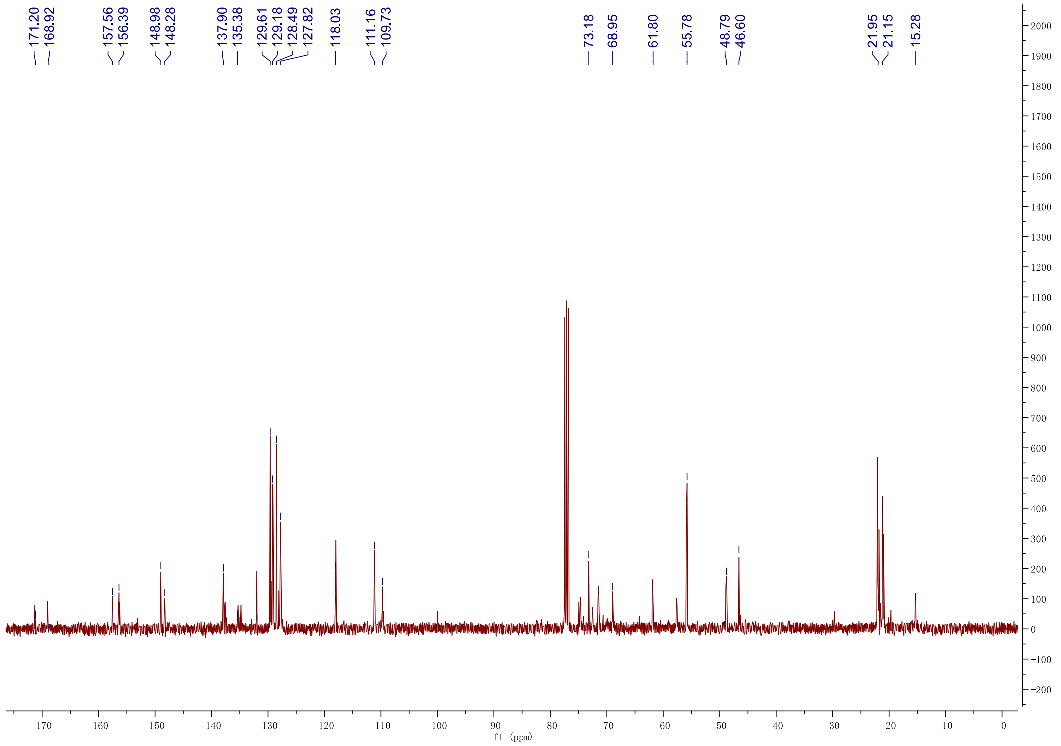

Figure 87 ^{13}C NMR spectrum of **I-35**

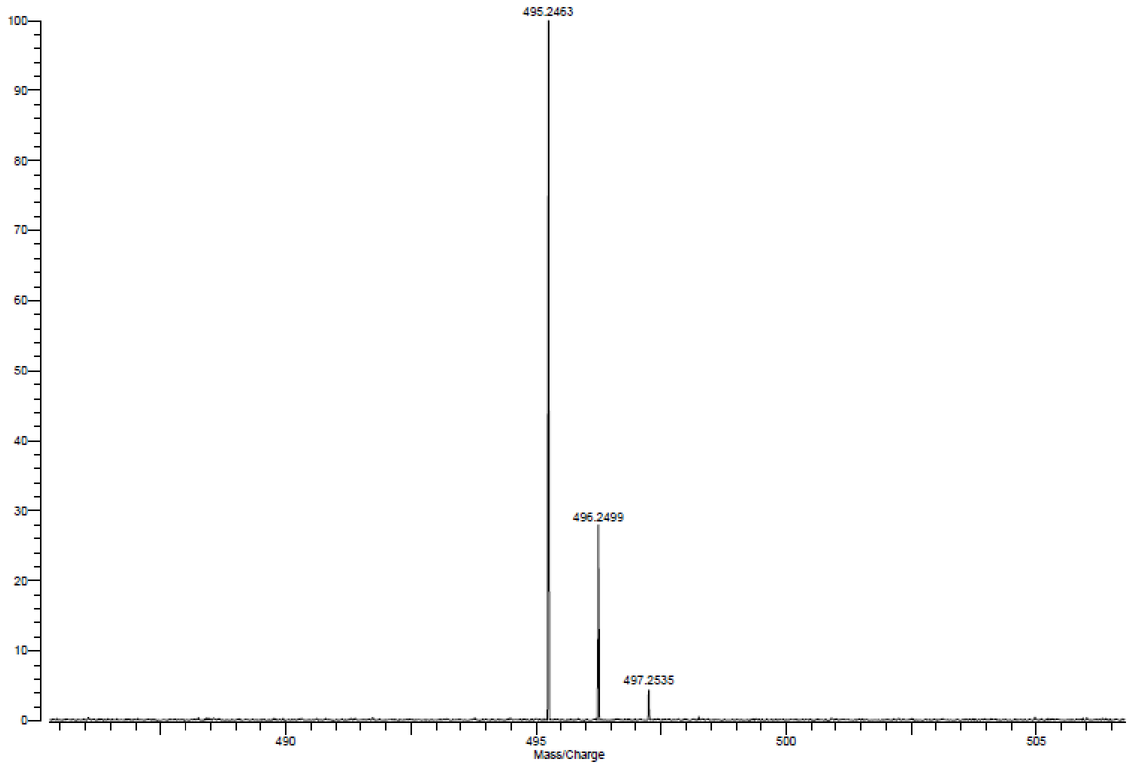

Figure 88 HRMS of **I-35**

I-36

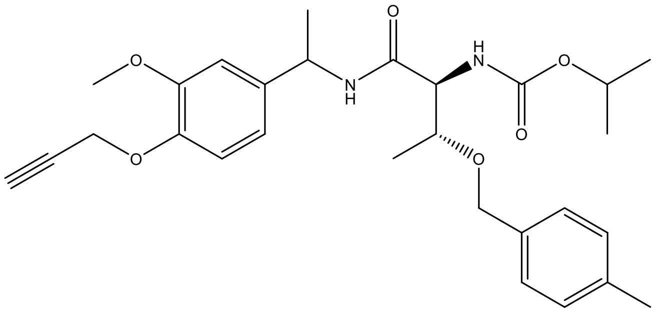

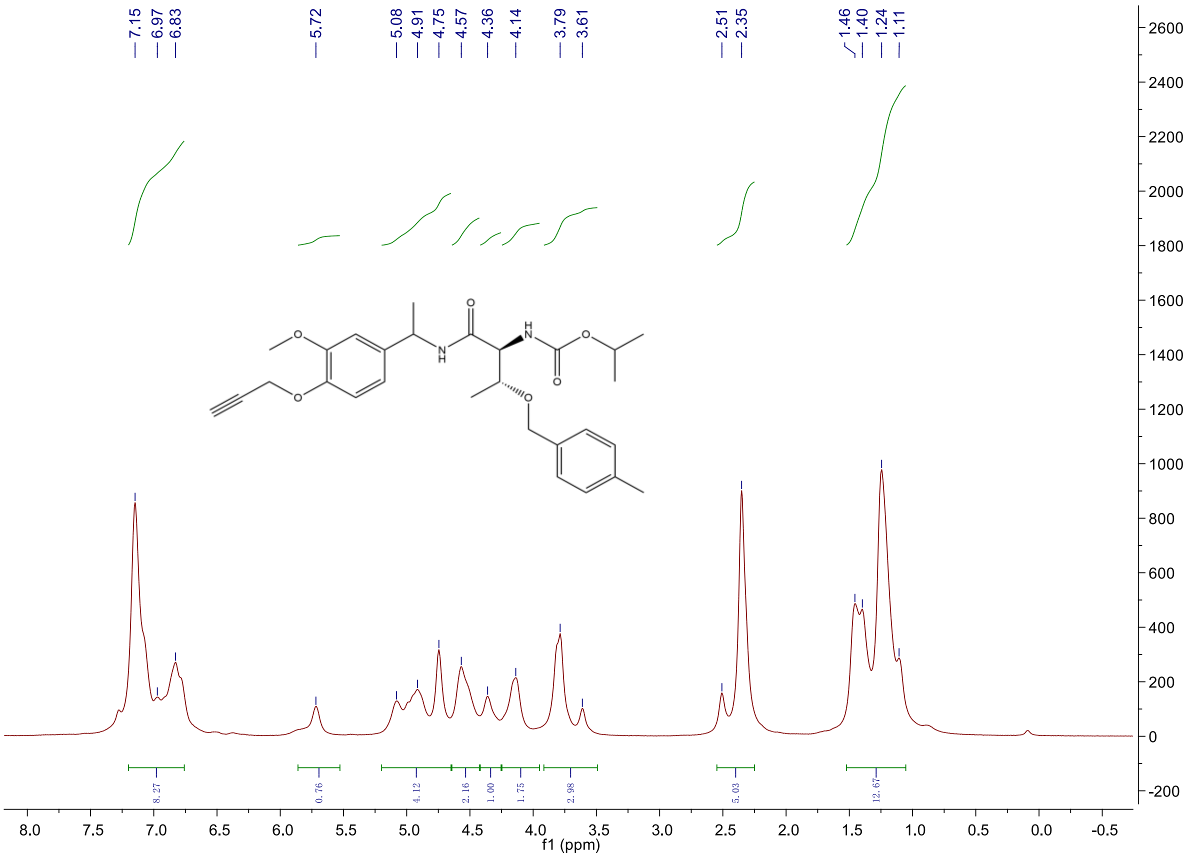

Figure 89 ^{1}HNMR spectrum of **I-36**

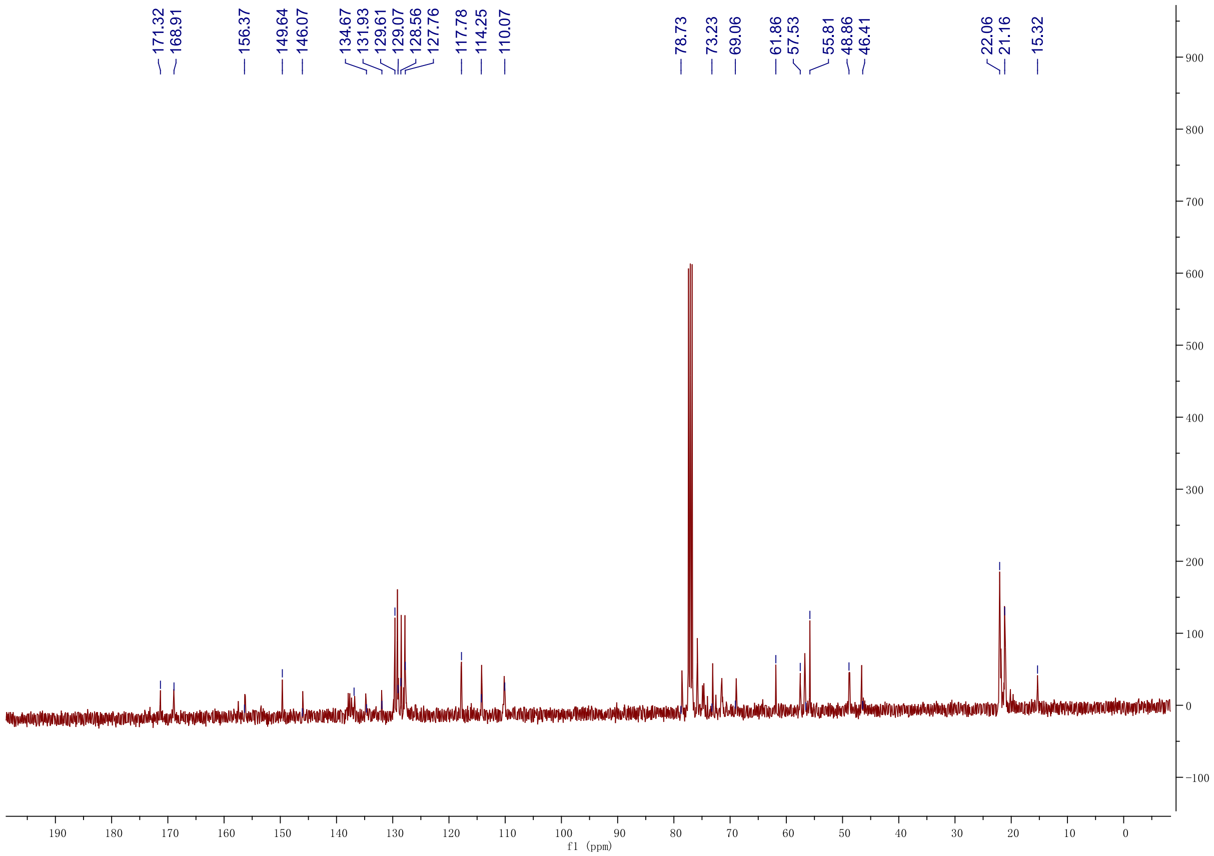

Figure 90 ^{13}C NMR spectrum of **I-36**

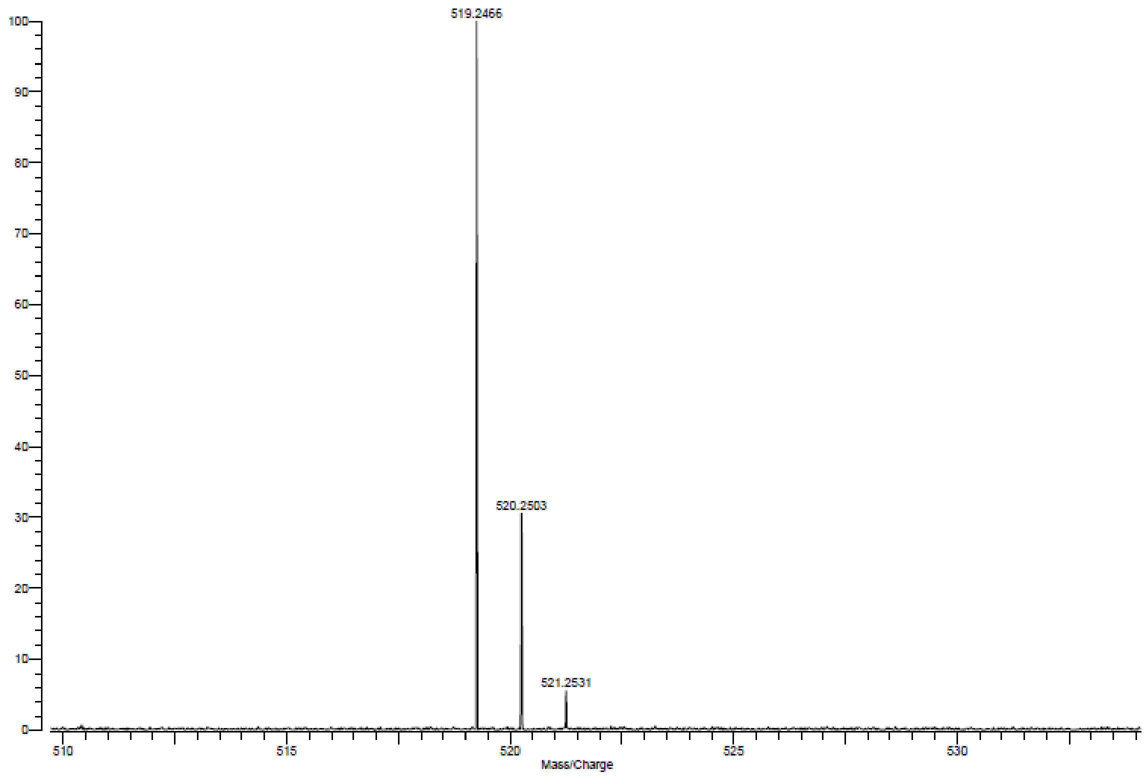

Figure 91 HRMS of **I-36**
